# Beyond Fang's fury: a computational study of the enzyme–membrane interaction and catalytic pathway of the snake venom phospholipase A_2_ toxin†

**DOI:** 10.1039/d4sc06511e

**Published:** 2025-01-02

**Authors:** Juliana Castro-Amorim, Alexandre V. Pinto, Ashis K. Mukherjee, Maria J. Ramos, Pedro A. Fernandes

**Affiliations:** a LAQV/Requimte, Departamento de Química e Bioquímica, Faculdade de Ciências da Universidade do Porto Rua do Campo Alegre, s/n 4169-007 Porto Portugal pafernan@fc.up.pt; b Institute of Advanced Study in Science and Technology Vigyan Path Garchuk, Paschim Boragaon Guwahati-781035 Assam India

## Abstract

Snake venom-secreted phospholipases A_2_ (svPLA_2_s) are critical, highly toxic enzymes present in almost all snake venoms. Upon snakebite envenomation, svPLA_2_s hydrolyze cell membrane phospholipids and induce pathological effects such as paralysis, myonecrosis, inflammation, or pain. Despite its central importance in envenomation, the chemical mechanism of svPLA_2_s is poorly understood, with detrimental consequences for the design of small-molecule snakebite antidotes, which is highly undesirable given the gravity of the epidemiological data that ranks snakebite as the deadliest neglected tropical disease. We study a member of the svPLA_2_ family, the Myotoxin-I, which is part of the venom of the Central American pit viper terciopelo (*Bothrops asper*), a ubiquitous but highly aggressive and dangerous species responsible for the most problematic snakebites in its habitat. Furthermore, PLA_2_ enzymes are a paradigm of interfacial enzymology, as the complex membrane–enzyme interaction is as important as is crucial for its catalytic process. Here, we explore the detailed interaction between svPLA_2_ and a 1 : 1 POPC/POPS membrane, and how enzyme binding affects membrane structure and dynamics. We further investigated the two most widely accepted reaction mechanisms for svPLA_2_s: the ‘single-water mechanism’ and the ‘assisted-water mechanism’, using umbrella sampling simulations at the PBE/MM level of theory. We demonstrate that both pathways are catalytically viable. While both pathways occur in two steps, the single-water mechanism yielded a lower activation free energy barrier (20.14 kcal mol^−1^) for POPC hydrolysis, consistent with experimental and computational values obtained for human PLA_2_. The reaction mechanisms are similar, albeit not identical, and can be generalized to svPLA_2_ from most viper species. Furthermore, our findings demonstrate that the sole small molecule inhibitor currently undergoing clinical trials for snakebite is a perfect transition state analog. Thus, understanding snake venom sPLA_2_ chemistry will help find new, effective small molecule inhibitors with anti-snake venom efficacy.

## Abstract 2

Investigation of the two most widely recognized catalytic pathways for snake venom-secreted phospholipases A_2_ (svPLA_2_s): the ‘single-water mechanism’ and the ‘assisted-water mechanism’, in a membrane model.

## Graphical abstract

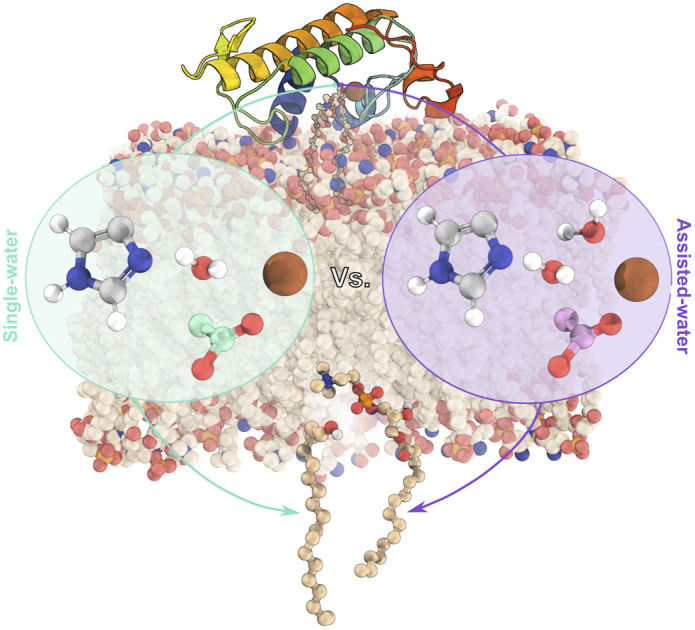

## Introduction

### Epidemiological background

Snakebite envenoming is currently regarded as the most lethal neglected tropical disease (NTD) by the World Health Organization (WHO). With 81–138 thousand fatalities each year and 400 thousand amputations and other permanent disabilities, snakebite is responsible for more deaths than all other NTDs combined.^1,2^ Most snakebite victims are from low- to middle-income regions where medical resources are scarce and health systems are ineffective, mainly in southeast Asia, sub-Saharan Africa, Latin America, and parts of Oceania.^3,4^

Antibody-based antivenom is the only available but centenary therapy that can effectively prevent or reverse the toxic effects of viper venom.^4–8^ However, it is quite expensive and needs to be transported and stored within a cold chain, which can be challenging. Therefore, this therapy is barely accessible to envenomed patients, particularly those from the rural areas of developing countries.^6–9^ Moreover, a significant number of victims (20–82%) experience adverse reactions to these antivenoms, which prompts them to stick with the traditional medicines, postponing the administration of targeted treatment.^10,11^ As a result, the WHO released a work plan in May 2019 to halve envenoming-related mortality and morbidity by 2030.^8,12,13^ This strategic initiative involves various objectives, including researching an effective and accessible treatment based on small-molecule inhibitors that can be administered outside hospital settings and stored in local communities.^12–14^ However, the poor atomic-level understanding of the molecular mechanisms of snake venom toxins limits the development of small-molecule toxin inhibitors.

### The fearsome terciopelo (*Bothrops asper*) and its venomous cocktail

The medically relevant snakes belong almost exclusively to two snake families, the elapids and the viperids.^15^ The latter family is responsible for a substantial portion of snakebite fatalities. Proteomic analysis has revealed the presence of four prominent families of proteins in most viper venoms: Zn^2+^-dependent metalloprotease (41–44%), secreted phospholipase A_2_ (29–45%), serine protease (4–18%) and l-amino acid oxidase (5–9%)^16–18^ (Fig. S1,† Section I).

The venom of each species comprises several svPLA_2_ enzyme isoforms, subdivided into acidic and basic. Due to its superior toxicity *in vivo*, the basic isoforms have been the focus of many studies.^16,19^ Basic PLA_2_ isoforms induce various toxic effects, whereas acidic isoforms have higher catalytic activity but do not produce obvious toxic consequences. The most common pathological effect of viper svPLA_2_ is myotoxicity. Other possible toxic effects include paralysis, anticoagulant action, blistering, edema-inducing activity, inflammation, and pain,^17,20–22^ thus playing a vital role in prey immobilization brought on by envenoming.

sPLA_2_s of elapids and viperids share a significant sequence identity of ≈60% with structural similarity ranging from 60% to 90%. They possess a highly conserved active site, and, expectedly, a similar reaction mechanism.^23–25^ Still, they differ in cell type affinities, with the viper venom attacking mostly myocytes and the elapid venom targeting motor neurons, even though exceptions are abundant.^15,26^ Accordingly, svPLA_2_s are categorized into two main groups: group IA, typically found in the venom of elapids, and group IIA, predominantly found in viperids. While both groups exhibit an overall conserved architecture, group IIA enzymes feature a more elongated C-terminal loop with 6 to 7 additional amino acids compared to group IA, influencing substrate specificity and interactions with phospholipid bilayers.^25^

The specific svPLA_2_ EC 3.1.1.4 (ref. 21, 27 and 28) studied here is found in the venom of a Latin American large pit viper, the *B. asper* (Viperidae family), commonly called “Terciopelo”,^4,28–30^ which is responsible for 50–80% of ophidic envenomations within its habitat.^3,4,19,21,30^ Some well-known researchers, like Picado^31^ and Bolaños^32^ have commented on its reputation as a fearsome, aggressive, agile, and unpredictable species.^33^ Bites from this viper may also result in death, disabilities, abortion, and other permanent sequelae due to the venom myotoxic and hemotoxic effects. In this and other vipers, the integrity of the plasma membrane of skeletal muscle fibers (sarcolemma) may also be disrupted.^20,21,34^ The Myotoxin-I (Mt-I) isoform, a basic Asp_48_ enzyme (svPLA_2_ numbering system), is a general representative of the large svPLA_2_ family of enzymes, and the focus of this paper.

### Snake venom-secreted phospholipase A_2_ (svPLA_2_) Mt-I

svPLA_2_ myotoxins are small (14–15 kDa) globular enzymes that belong to a large family of enzymes catalyzing the hydrolysis of membrane phospholipids at the sn-2 ester bond (Fig. S2†) in a Ca^2+^-dependent fashion.^27,35^ This process yields lysophospholipids and fatty acids, precursors of several signaling molecules involved in biological processes. In addition, this leads to an increase in Ca^2+^ in the muscle cells, followed by the release of K^+^ and ATP.^21,36^ As a result, a cascade of events is initiated, including the loss of mitochondrial function, the efflux of muscle-derived cytosolic components, widespread proteolysis, focal hypercontraction of myofilaments, and other degenerative processes that are yet to be identified.^20,21,26,37,38^

**Fig. 1:**
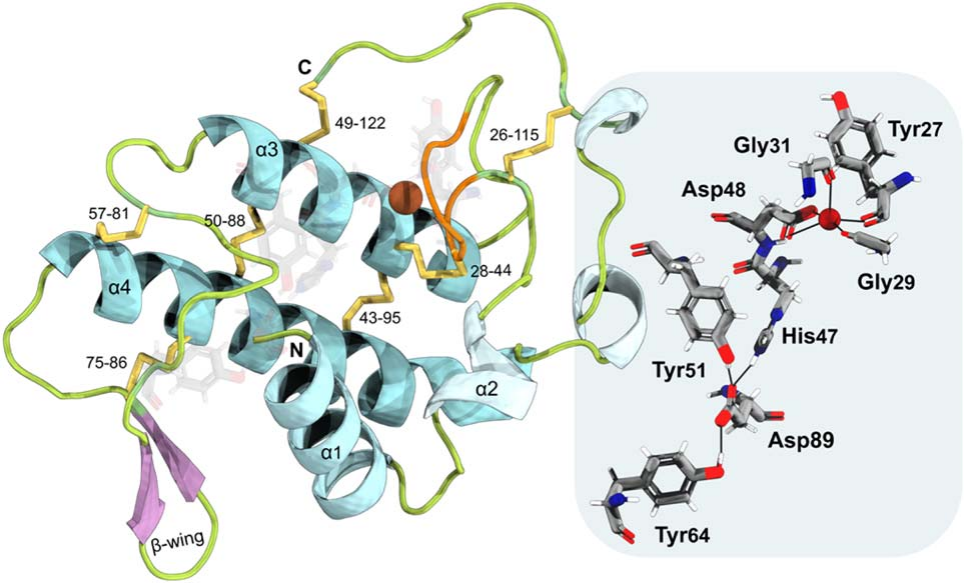
Cartoon representation of the svPLA_2_ tertiary structure. The active site residues (His_47_, Asp_48_, Tyr_51_, Tyr_64_, and Asp_89_) are shown as grey sticks, and the Ca^2+^ ion as a brown sphere. The disulfide bonds are shown in yellow. A close-up view of crucial residues of the catalytic network and Ca^2+^-binding loop is shown on the right.

*B. asper* Mt-I (BaMt-I) consists of 122 amino acid residues with seven disulfide bridges.^16,39^ This enzyme forms homodimers that are not covalently bonded in their native state nor glycosylated.^16,20^ Additionally, it adopts the classical group IIA sPLA_2_ fold with (i) a N-terminal α-helix 1 (α1) followed by a short helix, (ii) a Ca^2+^-binding loop, (iii) two antiparallel disulfide-linked α-helixes 3 and 4 (α3 and α4), where the active site cleft is deeply buried, (iv) two-stranded antiparallel β-sheet (β-wing) and (v) a flexible C-terminal loop (Fig. 1, left).^40–42^

Its catalytic network includes the most structurally conserved feature among sPLA_2_ enzymes, the His_47_/Asp_89_ dyad, which may interact through a low-barrier hydrogen bond involving the His_47_ nitrogen atom (Nε_2_) and the Asp_89_ carboxyl oxygen atom (O_δ1_).^43–45^ It also contains an Asp_48_, a crucial element for the binding of the Ca^2+^ cofactor, while Asp_89_, Tyr_51_, and Tyr_64_ aid in maintaining the proper His_47_ position for the reaction *via* hydrogen bonding.^46^ The Ca^2+^-binding loop is formed by the Tyr_27_, Gly_29_, and Gly_31_ backbone carbonyl groups and by the Asp_48_ β-carboxyl group (Fig. 1, right).^20,27,41^

### Membrane binding as a key to PLA_2_ catalysis

Despite being water-soluble, all PLA_2_s exhibit minimal activity towards non-aggregated substrates available in the bulk solvent. However, this behavior changes dramatically upon binding cell membranes, triggering a critical event in the PLA_2_s reaction cycle, known as “interfacial activation”.^43,47^ Hypothetically, it may facilitate the diffusion of the substrate along the hydrophobic channel for optimal catalytic activity (“substrate model”), lead to a change in the PLA_2_'s conformation making its active site more accessible for substrate interaction (“enzyme model”),^25,47^ or both. The result is a dramatic increase (up to 10 000-fold) in the reaction rate. Despite decades of research, the molecular basis for this rate enhancement is yet to be understood.

The non-covalent adsorption to a membrane is thought to rely on electrostatic and hydrophobic interactions between residues on the enzyme's interfacial binding surface (IBS) and cell membrane components.^48^ For example, at a neutral pH, both human group IIA secretory phospholipase A_2_ (hGIIA sPLA_2_) and the cotton mouth snake (*A. piscivorus piscivorus*) venom sPLA_2_ (App-D_48_ sPLA_2_) exhibit higher activity towards membranes enriched in negatively charged phospholipids than towards zwitterionic membranes.^49,50^ Charge reversal mutagenesis decreases the binding affinity of App-D48 sPLA_2_ and hGIIA sPLA_2_ to negatively charged surfaces. However, charge-compensation mutants, in which positively charged lysine residues were replaced with methionine residues, exhibited a modest increase in catalytic activity at the zwitterionic interface compared to the anionic one.^47,49,50^

The exact mechanism by which svPLA_2_ myotoxins exert their toxic effects has also been the subject of debate for many years. While their catalytic activity is essential for myotoxicity, some PLA_2_s with high catalytic activity exhibit minimal toxicity in living creatures. This observation has led to the hypothesis that the toxicity of these enzymes goes beyond simple catalysis and may involve additional, non-enzymatic, membrane-damaging effects (*i.e.*, packing defects, density, lipid protrusions, *etc.*).^51^ These toxic effects would depend on specific molecular domains other than the catalytic site, where the aforementioned phenomenon would play a substantial role.^52^

### Bridging the gap: exploring PLA_2_ putative reaction mechanisms

The PLA_2_ family of enzymes is known to operate using two potential catalytic mechanisms – the “single-water mechanism” and the “assisted-water mechanism”.^53^ These mechanisms were proposed based on the analysis of X-ray structures of several PLA_2_s derived from different organisms, which were complexed to inhibitors, substrate- and transition state-analogs,^54–58^ alongside mutagenesis studies and the knowledge of chemical mechanisms employed by related enzymes.^35,59–63^ The primary distinction lies in the number of water molecules involved.

**Fig. 2:**
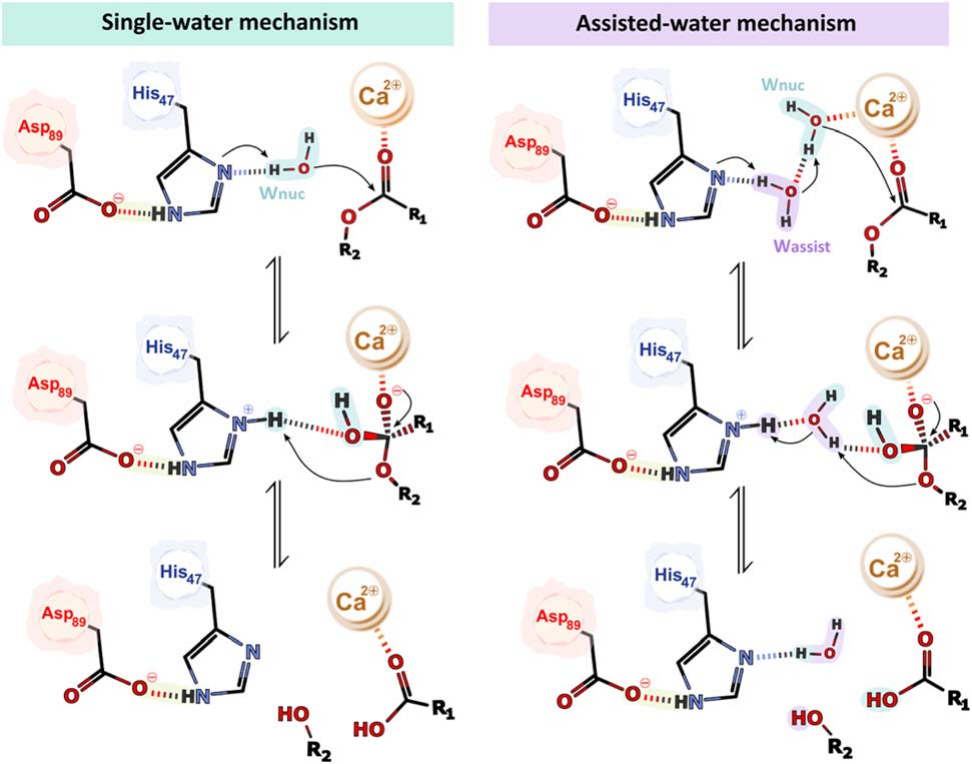
Schematic representation of the single- and the assisted-water mechanism.

The single-water mechanism proposed initially by Verheij in 1980,^59^ involves the presence of a hepta-coordinated Ca^2+^ ion formed by the two carboxylate oxygens of the Asp_48_ sidechain, three backbone carbonyl oxygen atoms of the Ca^2+^-binding loop (Tyr_27_, Gly_29_, and Gly_31_),^43,64,65^ and two water molecules.^64^ Both water molecules are displaced upon substrate binding, specifically by the phospholipid phosphate head and sn-2 carbonyl oxygen.^43,64^ The His_47_ residue, polarized by the His_47_(N^ε^)–(O^γ^)Asp_89_ hydrogen bond, deprotonates a catalytic water molecule, which is not coordinated by the Ca^2+^ ion, *via* the N^δ^ atom. Consequently, the generated hydroxide performs a nucleophilic attack at the sn-2 ester bond of the phospholipid substrate forming a tetrahedral oxyanion intermediate.^43,53,66,67^ The negatively charged tetrahedral intermediate is stabilized by the Ca^2+^ ion and by hydrogen bonding to the backbone amine of Gly_29_.^64^ Ultimately, the tetrahedral intermediate collapses upon deprotonation of His_47_ N^δH+^ by the sn-2 oxygen of the lysophospholipid leaving group (Fig. 2).^43,65,66^ Once the products are released, three water molecules move into the active site, from which two coordinate the Ca^2+^ ion, and the third replenishes the active cycle for nucleophilic attack at the subsequent turnover.^43,53^

In 1998, Yu proposed an alternate mechanism called the “assisted-water mechanism” that involves the participation of two water molecules.^68^ It includes the nucleophilic water (in analogy with the first mechanism) in the Ca^2+^ inner part coordination sphere (W_nuc_), hydrogen-bonded to an “assisting water” in the Ca^2+^ outer part coordination sphere (W_assist_), which, in turn, is hydrogen-bonded to the His_47_ N^δ^ atom.^69^ According to Yu and coworkers, in the first step of the mechanism, the Ca^2+^-bound W_nuc_ deprotonates the bridging W_assist_, which is in turn deprotonated by the His_47_ N^δ^ atom, thereby facilitating the reaction (Fig. 2). Subsequently, the generated Ca^2+^-bound hydroxide nucleophilically attacks the substrate sn-2 carbon atom. This reaction leads to the formation of a tetrahedral intermediate with a Ca^2+^-coordinated oxyanion. During the collapse of the tetrahedral intermediate, His_47_ protonates W_assist_, which, in turn, protonates the departing alkoxy oxygen.^68^

This research aims to investigate and disclose the effects of enzyme–membrane binding in the structures of the enzyme and membrane, in addition to the dynamics and the underlying catalytic mechanisms of svPLA_2_ compared to human synovial sPLA_2_ using QM/MM MD simulations.

Both human synovial PLA_2_ found in arthritic fluids and BaMt-I adopt the group IIA sPLA_2_ fold, with the former being non-toxic and the latter highly toxic, making this difference in bioactivity poorly understood. The study is also crucial for rationally developing transition-state-analog inhibitors with antiophidic properties.

## Methods

Here, we briefly summarize the methodology employed. A very detailed description of all methods employed can be found in the ESI,† Section II.

**Fig. 3:**
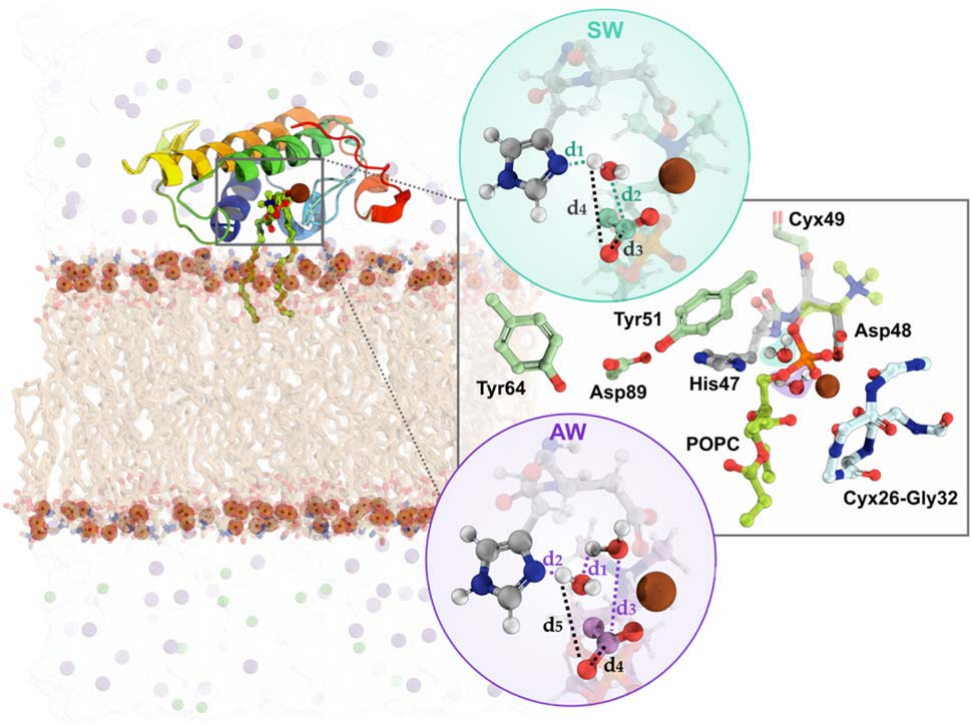
The modeled BaMt-I:POPC:membrane complex in a solvated box; (Left): the protein structure depicted in rainbow cartoon and the POPC substrate in lime green sticks. A light blue surface represents the solvent environment, while green and purple spheres denote chloride (Cl^−^) and sodium (Na^+^) ions. The lipid membrane is depicted in brown sticks, emphasizing the phosphate groups as dark orange spheres. (Right): A close-up view of the QM layer and the reaction coordinates (*ξ*_1_ – CV1 and *ξ*_2_ – CV2) used for the QM/MM MD calculations. Blue and purple circles represent the reaction coordinates used for the single – (SW) and the assisted-water (AW) pathways, respectively. In both cases, CV1 is represented by the same respective colors (blue and purple), and CV2 by black color in dashed lines.

We performed multiple computational methods to thoroughly investigate the catalytic mechanism of svPLA_2_ in a membrane model (Fig. S3, S4 and Table S1†). The process included (i) modeling of svPLA_2_:POPC:membrane complex in which the target protein structure with PDB code 5TFV was positioned at a weakly interacting distance (≈5 Å) over the upper leaflet of a 1 : 1 POPC/POPS bilayer (80 POPC:80 POPS per leaflet) (Fig. 3, left); Despite the target being homodimeric in its crystalline form, it has been demonstrated that PLA_2_s act as monomers when bound to lipid–water interfaces.^47^ Based on this evidence, we used the monomeric form to accurately reflect the enzyme's active state during interfacial activation; (ii) classical molecular dynamics (cMD) simulations using the GROMACS 2021.5 software for a total of 0.5 μs across six replicas (Table S2†); The Amber99SB-ildn force field was used for the protein, while the lipids were described by the Slipids-2020 force field;^70,71^ While the overall behavior among the replicas was relatively similar, the fifth replica was selected for subsequent studies due to its greater stability throughout the trajectory and more catalytically favorable distances; (iii) clustering analysis of the MD trajectories to identify structures with the active site properly preorganized to catalyze the phosphodiester hydrolysis for starting the mechanistic studies (Fig. S5 and S6†); (iv) DFT/MM MD umbrella sampling (US) simulations with the PBE functional employing the Gaussian double-ζ valence polarized (DZVP) basis set. DFT/MM calculations were carried out with the Gaussian plane wave formalism as implemented in CP2K, where an auxiliary plane-wave cutoff of 300 Ry was applied for the valence electrons, while the core electrons were treated using Goedecker–Teter–Hutter (GTH-PBE) pseudopotentials.^72^ The QM unit cell contained a total of 167 atoms with a neutral charge and a singlet spin (Fig. S7†). The reaction coordinates (*ξ*) for each mechanistic hypothesis were employed as collective variables (CVs) and were sampled at the DFT/MM level for 20 ps. For the single water (SW) mechanism, CV1 was defined as (*d*_1_ + *d*_2_) and CV2 as (*d*_3_ + *d*_4_), while for the assisted-water (AW) mechanism, CV1 was (*d*_1_ + *d*_2_ + *d*_3_) and CV2 was (*d*_4_ − *d*_5_), as depicted in Fig. 3, right. CV1 represents the protonation of the catalytic His_47_ and the nucleophilic attack by a water molecule on the carbonyl carbon of the substrate while CV2 represents both the cleavage of the substrate's ester bond and protonation of the departing lysophospholipid group (full details provided in the ESI†).

## Results and discussion

### Mapping BaMt-I:membrane interactions and their impact on the protein and membrane structure and dynamics

All the analysis regarding the svPLA_2_-membrane dynamic behavior can be found on the ESI, Fig. S8–S16,† Section III.

Since sPLA_2_ optimal activity is primarily restricted to the water–lipid interface, the presence of two binding sites, the catalytic domain and the aforementioned IBS, is crucial. The latter is located on a flat external region that surrounds the opening of the hydrophobic channel^25,73^ (Fig. S16†). Despite their conserved architecture and catalytic site, the sPLA_2_s have distinct interfacial binding properties, leading to distinct membrane affinities.^73,74^ Variations in the primary sequence of different PLA_2_s are depicted in Fig. S17.† In general, the IBS comprises a ring of basic and hydrophobic residues, in which the former interact electrostatically with the negatively charged head groups of anionic lipids^25^ and the latter, particularly the bulkier aromatic ones, interact and penetrate the phospholipid bilayer into the hydrophobic tail region, causing local membrane disordering. Fig. S16† reveals that the BaMt-I surface contains an IBS that is, indeed, arranged in a large patch of hydrophobic residues (Leu_2_, Ile_3_, Ala_6_, Leu_10_, Leu_16_, Phe_18_, Tyr_20_, Trp_30_, Met_108_, Ala_109_) flanked by several basic residues (Lys_7_, Lys_58_, Lys_60_, Arg_63_, Lys_105_) and some non-charged hydrophilic residues (Thr_22_, Thr_23_, Thr_61_).

**Fig. 4:**
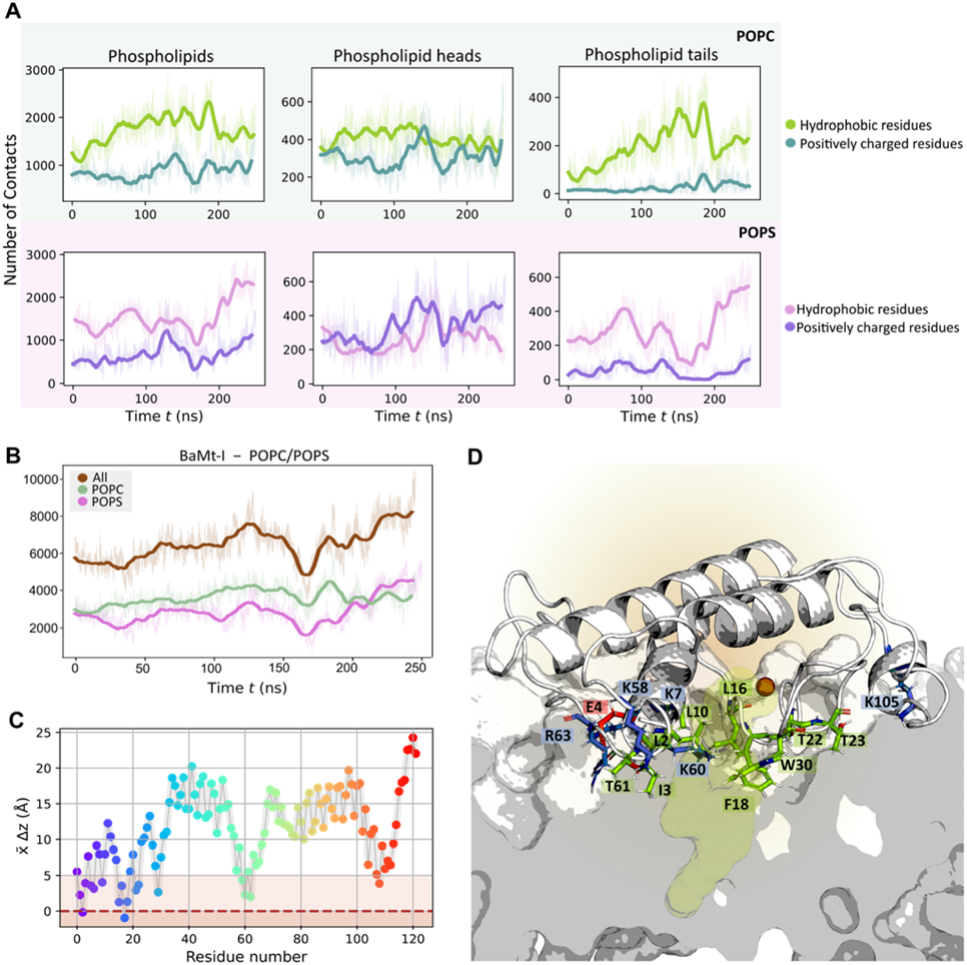
Protein–lipid interatomic contacts and a close-up view of the residues interacting with the membrane. (A) The average number of interatomic contacts with each phospholipid region over time. (B) The average number of interatomic contacts between BaMt-I and POPC (pale green) and POPS (pink) phospholipids over time; a minimum distance cutoff of 6 Å was applied to define a contact. (C) The average penetration depth of the BaMt-I residues into the lipid bilayer. The dark red dashed line represents the average membrane phosphate groups, and the light brown area represents the region occupied by the phospholipid heads (D) Snapshot illustrating the enzyme's position within the lipid bilayer and a closer view of the primary residues that penetrate it. Positively and negatively charged residues are depicted in blue and red sticks, respectively, hydrophobic anchors and non-charged hydrophilic residues in green.

The number of atomic contacts between BaMt-I and the mixed POPC/POPS bilayer along with an illustration of the residues interacting with the membrane is presented in Fig. 4A and B, respectively. On average, BaMt-I residues established a more significant number of contacts with the zwitterionic POPC lipids (3602 ± 561) than anionic POPS lipids (2887 ± 807). However, at approximately 170 ns, there was a noticeable increase in interactions between hydrophobic residues and the anionic phospholipids. In contrast to the hydrophobic residues, basic residues preferentially interacted with the headgroups rather than the tails, with which very few contacts were established (Fig. 4A).

The evolution of the total BaMt-I:membrane contacts throughout the simulation suggested a progressive insertion of BaMt-I into the membrane core. To assess the extent of penetration of BaMt-I into the lipid bilayer, we monitored the *z*-coordinate of the center of mass (COM) of each residue (Fig. 4C) and averaged along the whole trajectory. A residue was considered as inserted into the membrane if the *z*-distance of its COM was <5 Å of the average *z*-coordinate of the phosphate groups.

Fig. 4D highlights those residues that interacted and got partially inserted into the membrane – hydrophobic (Leu_2_, Ile_3_, Ala_6_, Leu_10_, Leu_16_, Phe_18_ and Trp_30_), basic (Lys_7_, Lys_58_, Lys_60_, Arg_63_ and Lys_105_), and non-charged hydrophilic residues (Thr_22_, Thr_23_ and Thr_61_).

Based on these findings, it might be possible that Lys_7_, Arg_63_, and Lys_105_ are the leading promoters of protein insertion into the 1 : 1 POPC/POPS bilayer. These residues likely aid in pulling the protein into the anionic lipid interface through electrostatic interactions. Furthermore, hydrophobic residues like Ile_3_, Phe_18_, Leu_10_, and Leu_16_ probably contribute to the enzyme's anchoring.^75,76^ Most of the residues align with those suggested by Salvador *et al.*^20^ and are believed to be essential for BaMt-I binding to the membrane.

**Fig. 5:**
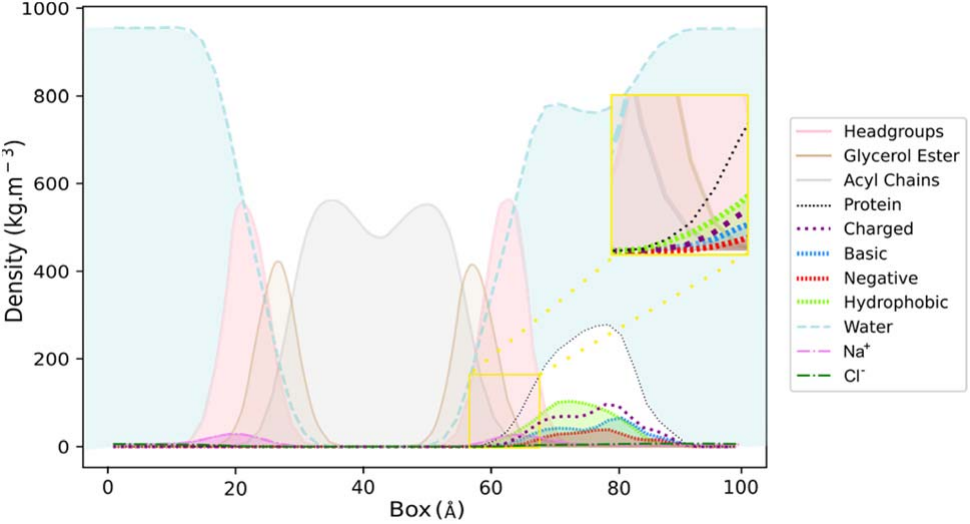
Partial density profile across the BaMt-I:membrane complex for membrane headgroups (pink), glycerol ester (tan) and acyl chains (grey), and protein (black dashed lines) with their hydrophobic (green), basic (marine blue) and negative (red) residues. The aqueous environment (blue), and counterions Na^+^ (magenta) and Cl^−^ (dark green) are also represented. A close-up view of the area of the protein that penetrated both the glycerol ester, and the acyl chains of the membrane is presented in a yellow box.

The visual inspection of the trajectory showed that BaMt-I promptly reached the membrane interface but did not penetrate extensively, embedding approximately 11 Å into the membrane, as indicated by the partial density profile (Fig. 5). In alignment with the previous results, Fig. 5 shows an evident predominance of hydrophobic residues at the IBS, compared to basic or acidic residues. The hydrophobic residues did not penetrate deeply into the membrane hydrophobic core, except for Ile_3_, Leu_16_, Leu_10_, and Phe_18_. Throughout the simulation, BaMt-I underwent rotational adjustments, characterized by increased penetration in the N-terminus region and an elevation of the C-terminal area concerning the membrane interface.

**Fig. 6:**
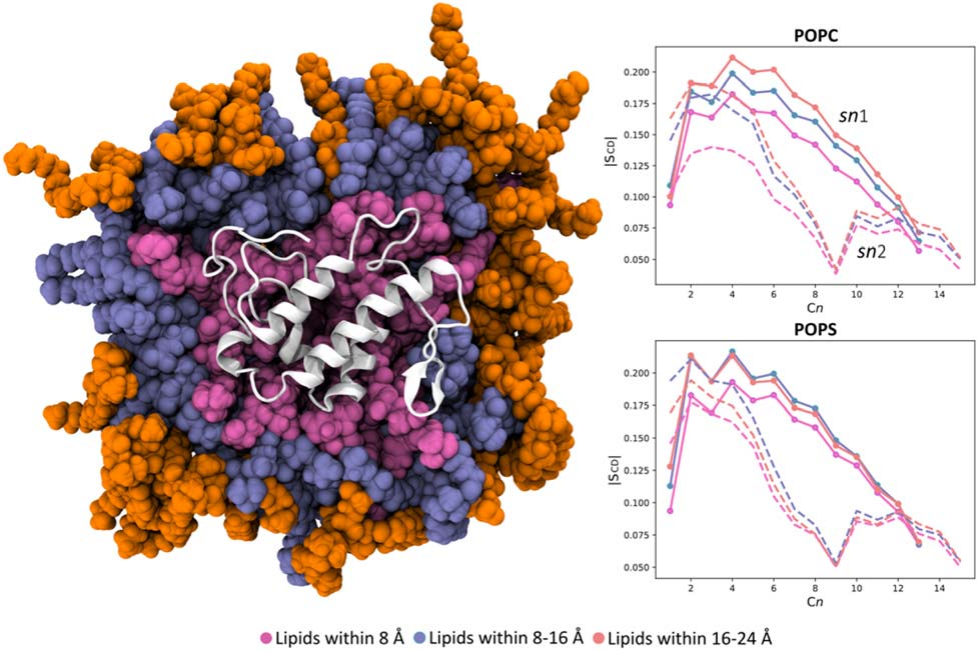
Definition of regions in the membrane surrounding BaMt-I. Three regions—8 (pink), 8–16 (iceblue), and 16–24 Å (orange)—were defined, each of which denotes a distance layer from the enzyme (white cartoon). Deuterium order parameters for carbon atoms in the sn-1 (line) and sn-2 (dashed line) hydrocarbon chains of POPC (top) and POPS (bottom) lipids were calculated in the defined regions.

Finally, we evaluated also the destabilizing impact of the enzyme on the molecular organization of the lipid bilayer by assessing the order parameter profiles of the phospholipids within three distinct regions (Fig. 6). It is a measure of the degree of rigidity or fluidity, *i.e.*, order or disorder, in lipid acyl chains within a membrane, quantifying their orientation relative to the membrane's normal axis. These regions were located at 8, 16, and 24 Å from the BaMt-I IBS. The deuterium order parameters (−*S*_CD_) for carbons of the sn-1 and sn-2 acyl chains of POPC and POPS were calculated within these regions. Results revealed that the acyl chain carbon's order parameters of both POPC and POPS in the vicinity of the enzyme were lower (lower −*S*_CD_ values) than the most distant ones (16 and 24 Å). These observations indicate a more disordered packing of the lipids in the presence of the enzyme. The observed results are justified by the substantial amount of the already demonstrated hydrophobic residues on the enzyme's IBS and the interaction of basic residues with the membrane. According to Salvador *et al.*,^20^ some of the residues that penetrate the lipid bilayer belong to a “myotoxic cluster” and are believed to be membrane-docking/disrupting sites. Their presence favors dispersion interactions with the bilayer non-polar hydrocarbon tails, which might change their local properties and further impact the neighboring areas.

Notably, BaMt-I IBS is characterized by the predominance of hydrophobic residues. Leu_2_, Ile_3_, Ala_6_, and Leu_10_ are part of the amphipathic N-terminal α-helix (residues 2-13), which participates in the substrate binding pocket^25,77–79^ and plays a crucial role in the orientation and binding mode to the membrane.^50,77–82^ These residues, along with Leu_16_, Phe_18_ and Trp_30_, stabilize the enzyme in the bilayer environment by hydrophobically anchoring it.

Studies have shown that mutations in bulky aromatic residues at the IBS significantly impact the affinity for zwitterionic membranes.^75,81,83,84^ This was the case for the acidic PLA_2_ from *N. naja atra*, where introducing mutations at Trp and Phe residues on the IBS led to a significant decrease in both zwitterionic membrane penetration and enzymatic activity (up to 50-fold decrease).^85^ In contrast, hGIIA PLA_2_, which lacks these bulky residues, displays low binding affinity towards zwitterionic membranes, favoring the binding to anionic membranes. However, when both Val_3_ and Val_31_ were mutated to a Trp residue, the hydrolytic activity towards zwitterionic interfaces improved.^86^ In the case of BaMt-I, Phe_18_ and Trp_30_ could have an essential role in the interfacial binding process in addition to substrate recognition.^25,87^

Unlike many other PLA_2_ enzymes (*e.g.*, human and porcine pancreatic PLA_2_),^88^ BaMt-I does not possess clusters of positive residues surrounding the IBS. Lys_7_, Arg_63_, and Lys_105_, also common in Lys_49_-PLA_2_-like proteins, have been proposed to form hydrogen bonds and salt bridges with the carbonyl oxygens of anionic lipids.^89^ Lys_60_ is thought to contribute to interfacial adsorption and electrostatic interactions with the substrate phosphate. Lys_7_ is highly conserved among PLA_2_s, and its mutation to glutamate in both the basic App-D_48_ sPLA_2_ myotoxin and in the hGIIA sPLA_2_, significantly decreases the adsorption and catalysis on anionic lipid bilayers.^50,81,89^ This residue and a patch of additional lysine residues at the end of the N-terminal segment—which are absent from BaMt-I—are thought to be responsible for PLA_2_'s anionic membrane selectivity.^73,78^ Moreover, Lys_105_Ala mutants in BthTX-I, a PLA_2_-like enzyme from the pit viper *B. jararacussu*, exhibited decreased membrane damaging activity yet did not eradicate it. These enzymes, along with Myotoxin II (*B. asper*) and crotoxin (*C. durissus terrificus*),^90^ preferentially bind to anionic phospholipids, emphasizing the role of electrostatic interactions in their interfacial binding affinity. Experimental data suggested that *ca.* 20% negatively charged phospholipids are needed for these enzymes to bind the membrane with high affinity due to charge–charge attractions.^83,91^

Therefore, the hydrophobic/basic nature of BatMt-I IBS may result in an enhanced ability to interact with both zwitterionic and anionic phospholipids, relative to the enzymes mentioned above, *i.e.* a higher affinity towards membranes.

### Structural insights into the pre-reactive state of svPLA_2_

The clustering of the whole molecular dynamics trajectory, based on reactive distances, revealed three main clusters of conformations primarily dictated by the distances between His_47_ N^δ^–C_popc,_ and Tyr_51_ OH–Asp_89_ O^δ1^.

**Fig. 7:**
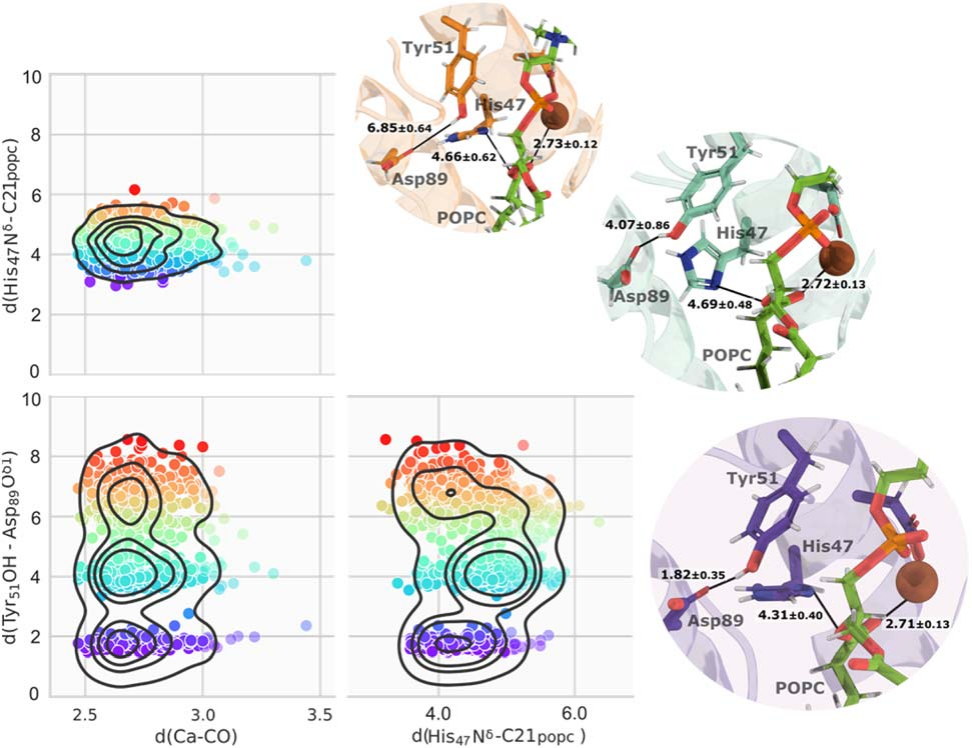
The three main clusters identified after filtering the Ca^2+^–CO_popc_, His_47_ N^δ^–C_popc,_ and Tyr_51_ OH–Asp_89_ O^δ1^ distances, represented by a color gradient. As the color changes from purple to red, it indicates that the structure becomes less ideal. Close-up views of representative structures from the three main clusters and respective distances are also shown. The tonality of each representative structure aligns with its corresponding cluster.

These distances exhibited considerable variations during the simulations (Fig. S9†). As illustrated in Fig. 7, only a minor portion of the trajectory contained distance-suitable conformations that agreed with the expected reactive-prone conformations proposed in the literature. This was caused by the distance between Tyr_51_ OH and Asp_89_ O^δ1^, which was large for most of the simulation (Fig. S9†). Notably, this dynamic interplay had a significant impact on the availability of productive conformations within the trajectory, resulting in only approximately 20% of the entire trajectory, represented in purple as shown in Fig. 7.

We re-clustered the conformations retrieved from the initial clustering process. A dominant cluster encompassed almost 75% of the retrieved microstates from the first clustering process with an average RMSd of 0.97 Å to the crystallographic structure, while the second cluster contained about 13% of the structures.

Upon examining the structures of each cluster representative, it was evident that the structural elements can be almost perfectly superimposed with minor variations around catalytic residues (Fig. S6†). The chosen structure, which contained two water molecules in the active site interacting with the Ca^2+^ ion and the His_47_ N^δ^ atom, was used for both mechanistic hypotheses.

**Fig. 8:**
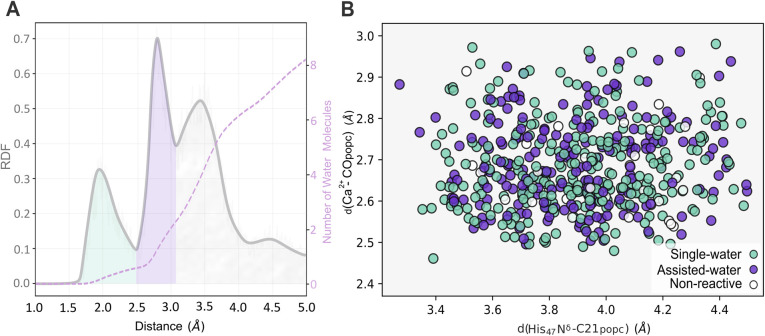
The RDF depicts the spatial distribution of water molecules relative to the midpoint of the Ca^2+^–His_47_ N^δ^–C21_popc_ plane. The dashed pink line represents the cumulative quantity of water molecules (A). Distribution of productive conformations for the single- and the assisted-water mechanisms in function of crucial distances between His_47_ N^δ^–C_popc_ and the Ca^2+^–C21_popc_ (B).

Three different solvation spheres are evident in the radial distribution function (RDF) of water molecules around the midpoint of the Ca^2+^–His_47_ N^δ^–C21_popc_ plane (Fig. 8A). The first and second solvation spheres, spanning approximately from 1.65 Å to 2.50 Å and 2.50 Å to 3.10 Å, exhibit well-defined peaks and accommodate an average of 0.8 and 2.1 water molecules, respectively. The third solvation sphere (≈3.10 Å to 4.20 Å), displays a broader distribution and contains an average of 6.0 water molecules. This third solvation sphere encompasses water molecules near the Asp_89_ O^δ1^ and His_47_ H^ε2^ atoms.

This study also explored the potential existence and dominance of productive conformations regarding the single- and assisted-water mechanisms. Thus, the productive conformations retrieved from the latter clustering step were further evaluated by examining the number of water molecules within the active center concerning the His_47_ N^δ^–C_popc_ and the Ca^2+^–CO_popc_ distances (Fig. 8B). Among the examined frames, 54% and 40% were suitable for the single- and the assisted-water mechanisms, respectively, while 6% were disregarded as non-productive conformations. These findings suggest that the energy penalty associated with the conformational arrangements required for catalysis is similar, making both pathways accessible.

Despite undergoing a complex clustering process to ensure conformity with literature and recent mechanistic studies on the PLA_2_ enzyme,^48^ the starting structure for the mechanistic study is thought to have minimal impact on the calculated free energy profile. This is because the QM/MM MD simulations mitigate any dependence on the starting structure chosen during the clustering analysis process. However, by selecting a structure with optimized catalytic distances with a low RMSD to the crystallographic reference, it is possible to carry out the mechanistic calculations with faster convergence results.

### Exploring svPLA_2_ possible reaction pathways

**Fig. 9:**
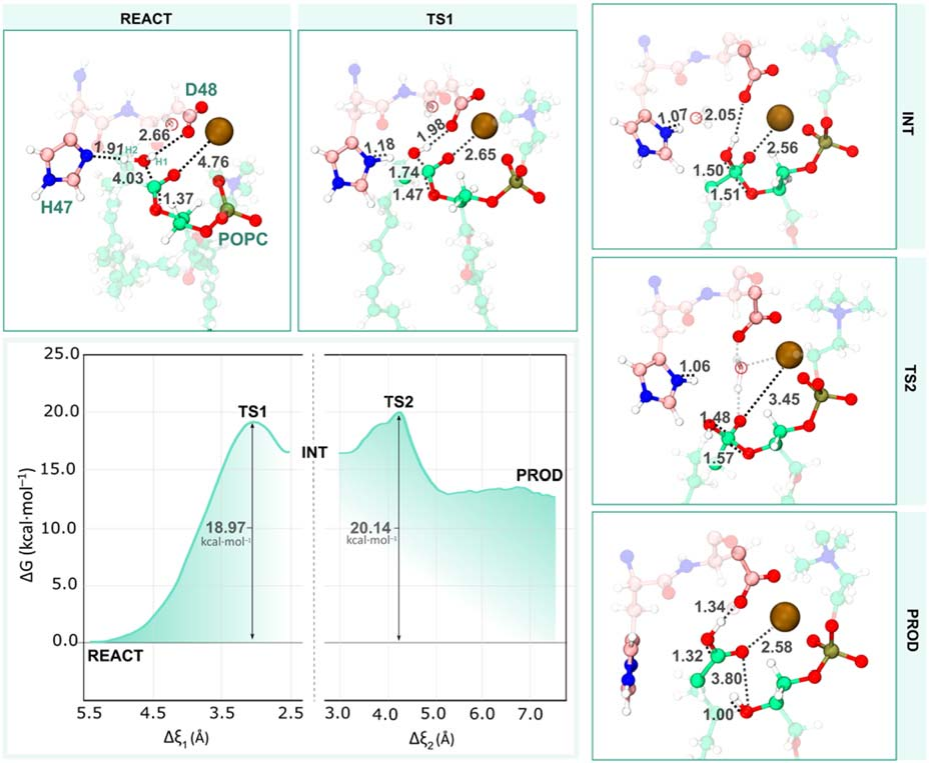
The free energy profile shows the single-water mechanism. The first peak represents the first transition state (TS1), where the nucleophilic attack takes place, and the second peak represents the second transition state (TS2) with partial hydrolysis of the sn-2 ester bond. Only the residues of the QM layer directly involved in the reaction are shown, while the MM layer is omitted for clarity. All distances are given in Å.

In the reactant state of the single-water mechanism (Fig. 9 – REACT), the nucleophilic water (W_nuc_) was well-positioned with respect to the His_47_ N^δ^ and the C_popc_ atoms, with distances of 1.72 and 4.03 Å, respectively, suggesting a favorable geometry for the first catalytic step. Notably, a hydrogen bond was formed between W_nuc_ and the O^δ2^ atom of the Asp_48_ residue. It has been shown that mutations at the Asp_48_ to Glu or Lys compromise the enzyme's activity by affecting its binding affinity for both Ca^2+^ and the phospholipid substrate within the active site.^60,63^ Despite not being coordinated with the Ca^2+^ ion (*d*_Wnuc–Ca_^2+^ ≈ 4 Å), the W_nuc,_ forms a short hydrogen-bond with the His_47_ N^δ^ atom and the negatively charged carboxyl group of the Asp_48_ O^δ^ base, resulting in a polarized state.

The umbrella sampling simulations along the reaction coordinates identified a stepwise pathway characterized by two distinct transition states (TS1 and TS2). At *ξ* = +3.00 Å, the first transition state (TS1) was formed. Here, the polarized W_nuc_ was deprotonated by the basic His_47_ residue, generating a nucleophilic hydroxide ion (HO^−^) hydrogen-bonded to Asp_48_ O^δ2^, which, in turn, initiated the nucleophilic attack on the phospholipid substrate's ester bond (C_popc_). These events occurred synchronously, as evidenced by distances between H2_wat_–His_47_ N^δ^ (*d*_1_) and O_Wat_–C_popc_ (*d*_2_) in TS1, which decreased to 1.18 and 1.74 Å, respectively. This further indicates that the catalytic water was fully deprotonated and that the newly formed hydroxide ion species, while not fully stabilized, was already partially bonded to C_popc_ (Fig. 9 – TS1).

Along the TS1 decay, the O_Wat_–C_popc_ distance decreased from 1.74 to 1.50 Å. As a result, a tetrahedral oxyanion intermediate was formed at *ξ* = +2.54 Å, where the oxygen on the oxyanion hole, stabilized by the Ca^2+^ cofactor, became negatively charged and C_popc_ adopted a sp^3^ hybridization (Fig. 9 – INT). This first step yielded a free energy barrier of 18.97 kcal mol^−1^, which aligns with the experimental data for hydrolase-related reaction mechanisms.^48,85,92^

As the reaction progressed to the TS2 at *ξ* = +4.23, the active site underwent a significant hydrogen-bond network rearrangement (Fig. 9 – TS2). The initial hydrogen bond between the OH^−^ at the sn-2 ester bond and the Asp_48_ O^δ2^ atom is disrupted due to the entrance of a water molecule that bridges this interaction. This water molecule also forms a hydrogen bond with the carbonyl oxygen of the substrate while simultaneously coordinating with the Ca^2+^ ion (depicted by light gray dashed lines). These events caused the carbonyl oxygen of the substrate (CO_popc_) to reorient away from the Ca^2+^ ion, up to an average distance of 3.45 Å, which may have contributed to an increase in the barrier. The free energy barrier associated with TS2 was 20.14 kcal mol^−1^ concerning the initial reactant state.

Finally, the sn-2 ester bond of the substrate quickly broke, accompanied by a notable increase in the C_popc_–O_popc_ (*d*_3_) distance, which rose from 1.57 to 3.80 Å. Synchronously, the resulting negatively charged oxygen on the lysophospholipid group deprotonated the positive His_47_ N^δ^H. The newly formed fatty acid was also observed to be transiently deprotonated by the neighboring Asp_48_ until the final product was obtained (Fig. 9 – PROD). These results further confirm that Asp_48_ plays a significant role in catalysis. Specifically, the hydrogen bonds formed between Asp_48_ and the catalytic water (Asp_48_ O^δ2^–W_nuc_), as well as with the fatty acid group (Asp_48_ O^δ1^–COO^−^_popc_), prove its role in activating the nucleophile and stabilizing the products, which were stabilized by several active site residues. The phosphate group of the lysophospholipid product got stabilized through interactions with the Ca^2+^ ion, Lys_60_, and Gly_31_ from the Ca^2+^-binding loop. The free energy associated with the enzyme-bound products was 13.52 kcal mol^−1^, showing an endergonic character.

Representative snapshots of each stationary state and respective interatomic distances are presented in Fig. 9.

**Fig. 10:**
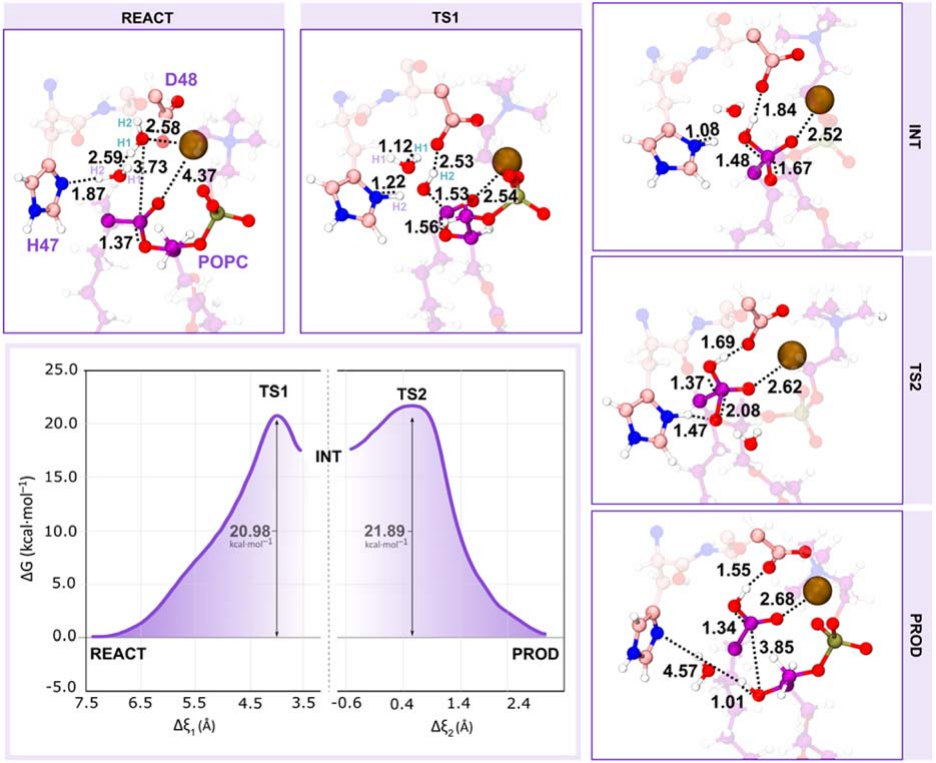
The free energy profile shows the assisted-water mechanism. The first peak represents the first transition state (TS1), where the nucleophilic attack takes place, and the second peak represents the second transition state (TS2) with partial hydrolysis of the sn-2 ester bond. Only the residues of the QM layer that are directly involved in the reaction are shown, while the MM layer is omitted for clarity. All distances are given in Å.

Concerning the alternative assisted-water mechanism, in the reactant state (Fig. 10 – REACT), the W_nuc_ H–W_assist_ O, W_assist_ H–His_47_ N^δ^ and W_nuc_ O–C_popc_ average distances were 2.59, 1.87, and 3.73 Å, respectively, favoring both the proton transfers and the nucleophilic attack. Furthermore, consistent with earlier literature and in contrast to the previous observations in the single-water pathway, the catalytic water (W_nuc_) coordinates with the Ca^2+^ ion.

The coordination of the W_nuc_ to the Ca^2+^ ion completes the characteristic Ca^2+^ coordination sphere observed in sPLA_2_s, which may lead to the movement of the CO_popc_ from the cofactor to approximately 4.37 Å.

We carried out umbrella sampling simulations, and the results indicate that the assisted-water mechanism also follows a stepwise reaction, featuring two transition states (TS1 and TS2) at *ξ* = +4.10 Å and +0.45 Å, respectively. The reaction is initiated by the deprotonation of the W_nuc_ by W_assist_, generating a nucleophilic HO^−^ and a highly unstable H_3_O^+^. Simultaneously, the catalytic His_47_ residue deprotonates the bridging water molecule (W_assist_), and the previously produced HO^−^, which is again hydrogen bonded to His_47_ N^δ^ and Asp_48_ O^δ2^, attacks the substrate sn-2 bond. This resulted in the formation of the TS1 (Fig. 10 – TS1), in which the O_Wnuc_–C_popc_ shortened to 1.53 Å and the C_popc_–O_popc_ distance increased to 1.56 Å. Synchronously, as the CO_popc_ becomes negatively charged after the HO^−^ nucleophile, the distance between the CO_popc_ and the Ca^2+^ ion decreased from 4.37 to 2.54 Å. The free energy barrier associated with this step was 20.98 kcal mol^−1^. As a result, a tetrahedral oxyanion intermediate was formed (Fig. 10 – INT), with the sp^3^ hybridization of the C_popc_ bond and the stabilization of the CO_popc_ negative charge by the Ca^2+^ ion. At TS2 (Fig. 10 – TS2), the C_popc_–O_popc_ bond stretched up to 2.08 Å as the His_47_ suffered an orientation shift to transfer its proton to the leaving lysophospholipid group (O_popc_). The active role of the W_assist_ concluded after the deprotonation of W_nuc_, resembling the second step of the single-water pathway. The free energy barrier of this step is the highest of the reaction cycle, 21.89 kcal mol^−1^ above the reactants, becoming the rate-limiting transition state.

Ultimately, the substrate sn-2 ester bond broke along the path from the TS2 to the products, resulting in a distance of 3.85 Å and the collapse of the tetrahedral structure. As in the single-water mechanism, the newly formed fatty acid product also interacted with the neighboring Asp_48_, evidencing the catalytic role of the latter (Fig. 10 – PROD). The second step exhibits a pronounced free energy decrease at PROD, reaching 0.1 kcal mol^−1^. Furthermore, some active site residues played a stabilizing role regarding the products. The phosphate group of the lysophospholipid product got stabilized through interactions with the Ca^2+^ ion, and residues Lys_60_, Trp_30_, and Gly_31_ from the Ca^2+^-binding loop. Additionally, the Ca^2+^ ion, Asp_48_, and Gly_29_ stabilized the fatty acid product. Except for the Gly_29_ and Trp_30_ residues, the same interactions were observed in the single-water pathway.

Fig. S18–S24† illustrate the convergence of the free energy profiles and Fig. S25 and S26† depict changes in atomic distances over time, along with the corresponding standard deviations.

The results have shown that both the single- and the assisted-water mechanisms proceeded in a stepwise fashion. The former displayed a lower activation energy of 20.14 kcal mol^−1^ compared to the 21.89 kcal mol^−1^ necessary for the latter. The obtained free energy barriers are close when considering the inherent MUE associated with computational methods (≈3 kcal mol^−1^). A comparison of the activation free energies associated with the breaking and formation of the same bond type leads to a significant error cancellation, driving the comparison into the area of meaningfulness. Additionally, the calculated energies correspond well to those obtained from computational studies.^48^ Although both reactions exhibited endergonic character (Δ*G*° = 13.52 kcal mol^−1^ and 0.1 kcal mol^−1^), the assisted-water pathway displayed a thermodynamically and kinetically more favorable product state.

While product formation occurs in both reaction cycles, the enzyme's complete structural and functional reconstitution is not achieved. Prior research indicates that the full regeneration of PLA_2_ enzymes typically requires the influx of three water molecules into the active site following the release of products,^25,43^ likely equalizing the free energy at the end of the cycle. Among these, two coordinate the Ca^2+^ ion while the third replenishes the active cycle for subsequent nucleophilic attack. In this case, the absence of such a water exchange process could contribute to the observed endergonic character. Furthermore, slight conformational changes within the active site, coupled with the aforementioned observation, likely hinder the precise realignment of the active site for subsequent turnover.

Overall, the interplay between the lower activation energy obtained for the single-water pathway and the less endergonic nature of the assisted-water pathway compensate for each other's thermodynamic limitations.

### Using the QM/MM transition states to design potent svPLA_2_ transition-state-analog inhibitors

Understanding how PLA_2_ enzymes behave at the atomic level is critical to designing next-generation drugs that can effectively block the enzyme's activity, complementing or even outperforming antibody-based therapies. By designing transition state analogs, molecules that mimic the short-lived, high-energy transition state of the reaction, it is possible to develop highly potent and specific svPLA_2_ inhibitors with minimal off-target effects. Varespladib, a PLA_2_s inhibitor and the only small-molecule antidote reaching clinical trials against snakebite has proven effective against multiple catalytic PLA_2_s from snake venom and non-catalytic PLA_2_-like toxins from several medically important snakes.^93–95^*In vitro* and *in vivo* studies have also been carried out on BaMt-I, showing that Varespladib neutralizes its cytotoxic and myotoxic effects.^95^ Studies suggest that Varespladib can interfere with the interfacial activation process by interfering with or reducing the ability of the enzyme to disrupt the integrity of the plasma membrane in muscle cells.^95^

**Fig. 11:**
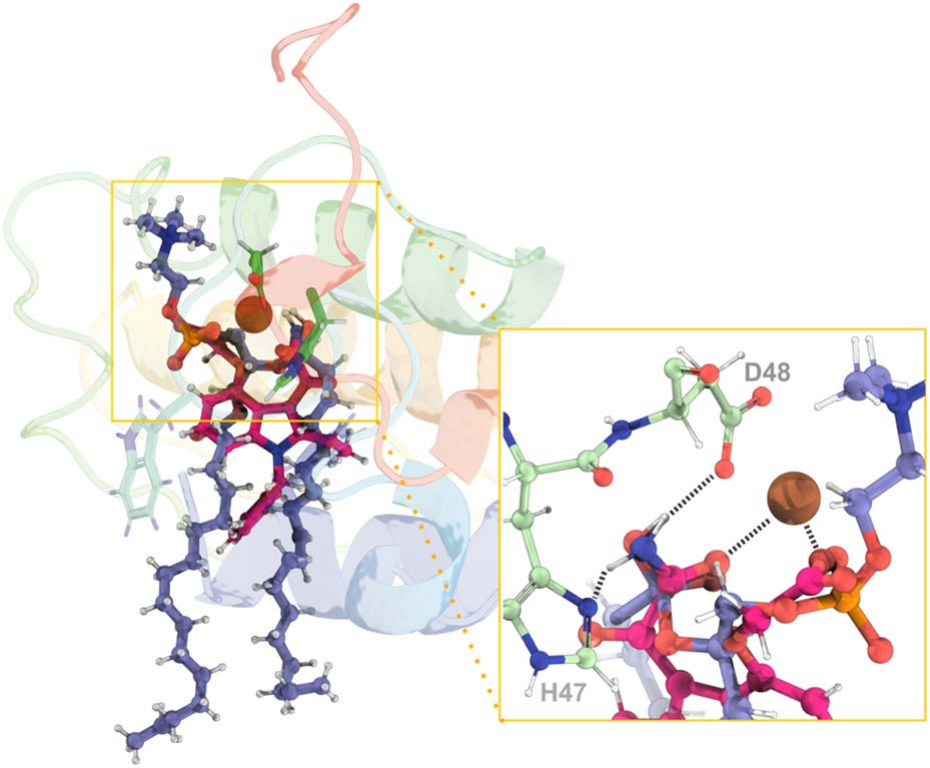
(Left) Superimposition of the assisted-water rate-limiting transition state structure obtained through the QM/MM MD calculations reported here (purple sticks) and the structure of the hGIIA PLA_2_ inhibitor, Varespladib (pink sticks) from the structure with the PDB ID 6PWH. (Right) A close-up view of the reaction region from another angle with the hydrogen bonds evidenced by dashed black lines demonstrates a high degree of similarity between the two ligands. Given the remarkable similarity in the position and structure of the single-water transition state, it was not included for clarity.

By superimposing the obtained rate-limiting transition state geometries (TS2 from both catalytic pathways) with Varespladib from the co-crystallized X-ray structure of Mt-II isolated from *Bothrops moojeni* (PDB code: 6PWH)^96^ (Fig. 11), we observed that the inhibitor mimics the crucial interactions found at the transition states reported here. Particularly, the carboxylate group of the drug mimics the negatively charged oxygen of the substrate phosphate group and the carboxamide group emulates the hydroxide ion (TS2, Fig. 9 and 10). In addition, the amine moiety of the carboxamide forms hydrogen bonds with the catalytic His_47_ and Asp_48_, and the Gly_29_ of the Ca^2+^-binding loop, mimicking the catalytic water molecule. We also observed π-stacking interactions between Trp_30_ and the benzyl group.

The fact that varespladib so accurately mimics the obtained rate-limiting transition states demonstrates that it is an effective transition-state analog. This supports the approach of guiding drug discovery on the accurate structures of rate-limiting transition states identified by QM/MM calculations. Therefore, the results presented here can be employed to enhance existing drugs' efficacy and selectivity or develop entirely new ones.

## Conclusions

In this work, we performed molecular dynamics simulations of the svPLA_2_ toxin, BaMt-I, bound to a POPC substrate in a 1 : 1 POPC/POPS mixture membrane bilayer. Analysis of the bound complex showed an abundance of hydrophobic residues in its interfacial binding surface, which distinguishes it from other PLA_2_ enzymes that are characterized by a stronger cationic nature (*e.g.* hGIIA PLA_2_, porcine PLA_2_, AppD48). The latter selectively bind anionic interfaces. Additionally, simulation results have confirmed that BaMt-I mainly interacted peripherally with the membrane, with low penetration depth. Following the existing literature, the key residues responsible for facilitating penetration were identified as Phe_18_, Ile_3_, and Trp_30_. On the other hand, basic residues, such as Lys_7_ and Lys_105_, were found to play a significant role in forming electrostatic and hydrogen bonds with the membrane. These results further suggest that BaMt-I may interact with zwitterionic and anionic interfaces. This hypothesis is corroborated by the surface charge distribution on its interfacial binding surface, the observed binding interactions, and the contacts established between BaMt-I and both types of phospholipids during simulation.

Furthermore, BaMt-I disrupted the lipid packing within the membrane, while the membrane environment, in turn, prompted conformational changes in the protein. Thus, the direct hydrolysis of phospholipids seems to be, indeed, a prominent mechanism driving toxicity, although membrane destabilization may occur as a secondary effect of enzyme activity through its interaction and light penetration. Moreover, the combined action of multiple svPLA_2_ enzymes may also play a role in destabilizing the phospholipid bilayer. After binding to the lipid bilayer, these enzymes accumulate at the interface, releasing hydrophobic reaction products, *i.e.*, lysophospholipids and fatty acids. These products can activate additional PLA_2_ enzymes and alter the physical properties of the membrane, destabilizing it.

We have investigated two distinct reaction mechanisms: the single-water pathway, which proceeded in two steps with a nucleophilic water molecule uncoordinated to the Ca^2+^ cofactor, and the assisted-water pathway, which also followed two steps, however, with a metal-coordinating nucleophilic water molecule. Although both pathways were mechanistically viable, with a small barrier gap (≈2 kcal mol^−1^), and catalytically-competent, it seems that the single-water pathway is more likely to occur, as evidenced by the analysis of reactive conformations and the lower activation-free energy barrier. However, the assisted-water pathway is thermodynamically more favorable. While the single water yielded a more endergonic product than the assisted-water, the final free energy will likely be equivalent after the reaction cycle is completed. Nonetheless, while it is suggested that the reaction mechanism may be conserved across species due to the significant structural similarity among PLA_2_ enzymes, further investigation is necessary to validate this generalization, particularly concerning PLA_2_s from elapids. Additionally, the subtle differences between the two proposed mechanistic pathways suggest that these mechanisms could potentially converge in other snake species.

Furthermore, mimicking rate-limiting transition states has emerged as an attractive approach for the design of next-generation inhibitory drugs. The evident similarity that emerged from this study between the coordination mode of the clinical trial candidate, Varespladib, and the QM/MM rate-limiting transition states demonstrated and reinforced the potential of using this powerful strategy in drug discovery.

## Data availability

The data supporting this article have been included as part of the ESI.†

## Author contributions

J. C. A.: conceptualization, methodology, software, validation, formal analysis, investigation, visualization, writing – original draft, writing – review & editing. A. V. P.: methodology, software, writing – review & editing. A. K. M.: writing – review & editing. M. J. R.: conceptualization, writing – review & editing, supervision, funding acquisition. P. A. F.: project administration, conceptualization, writing – review & editing, supervision, funding acquisition.

## Conflicts of interest

There are no conflicts to declare.

## Supplementary Material

SC-016-D4SC06511E-s001

SC-016-D4SC06511E-s002

## References

[cit1] Chippaux J. P. (2017). J. Venomous Anim. Toxins Incl. Trop. Dis..

[cit2] Kasturiratne A., Wickremasinghe A. R., de Silva N., Gunawardena N. K., Pathmeswaran A., Premaratna R., Savioli L., Lalloo D. G., de Silva H. J. (2008). PLoS Med..

[cit3] Gutierrez J. M. (2014). J. Venomous Anim. Toxins Incl. Trop. Dis..

[cit4] Otero-Patino R. (2009). Toxicon.

[cit5] Xie C., Albulescu L. O., Bittenbinder M. A., Somsen G. W., Vonk F. J., Casewell N. R., Kool J. (2020). Biomedicines.

[cit6] da Costa Neves-Ferreira A. G. , Valente R. H., Perales J. and Domont G. B., in Handbook of Venoms and Toxins of Reptiles, ed. S. P. Mackessy, Taylor & Francis/CRC Press, 2009, pp. 259–284

[cit7] Kalita B., Mackessy S. P., Mukherjee A. K. (2018). Expert Rev. Proteomics.

[cit8] Albulescu L. O., Xie C., Ainsworth S., Alsolaiss J., Crittenden E., Dawson C. A., Softley R., Bartlett K. E., Harrison R. A., Kool J., Casewell N. R. (2020). Nat. Commun..

[cit9] Liu C. C., Wu C. J., Hsiao Y. C., Yang Y. H., Liu K. L., Huang G. J., Hsieh C. H., Chen C. K., Liaw G. W. (2021). J. Proteomics.

[cit10] Otero-Patino R., Segura A., Herrera M., Angulo Y., Leon G., Gutierrez J. M., Barona J., Estrada S., Pereanez A., Quintana J. C., Vargas L. J., Gomez J. P., Diaz A., Suarez A. M., Fernandez J., Ramirez P., Fabra P., Perea M., Fernandez D., Arroyo Y., Betancur D., Pupo L., Cordoba E. A., Ramirez C. E., Arrieta A. B., Rivero A., Mosquera D. C., Conrado N. L., Ortiz R. (2012). Toxicon.

[cit11] Puzari U., Fernandes P. A., Mukherjee A. K. (2022). J. Ethnopharmacol..

[cit12] Williams D. J., Faiz M. A., Abela-Ridder B., Ainsworth S., Bulfone T. C., Nickerson A. D., Habib A. G., Junghanss T., Fan H. W., Turner M., Harrison R. A., Warrell D. A. (2019). PLoS Neglected Trop. Dis..

[cit13] Preciado L. M., Pereañez J. A., Comer J. (2020). Toxins.

[cit14] Puzari U., Fernandes P. A., Mukherjee A. K. (2021). J. Med. Chem..

[cit15] Oliveira A. L., Viegas M. F., da Silva S. L., Soares A. M., Ramos M. J., Fernandes P. A. (2022). Nat. Rev. Chem.

[cit16] Angulo Y., Lomonte B. (2009). Toxicon.

[cit17] Alape-Giron A., Sanz L., Escolano J., Flores-Diaz M., Madrigal M., Sasa M., Calvete J. J. (2008). J.
Proteome Res..

[cit18] Fernandez J., Gutierrez J. M., Angulo Y., Sanz L., Juarez P., Calvete J. J., Lomonte B. (2010). Biochimie.

[cit19] Gutierrez J. M., Lomonte B. (1995). Toxicon.

[cit20] Salvador G. H., Dos Santos J. I., Lomonte B., Fontes M. R. (2017). Biochimie.

[cit21] Fernandez J., Caccin P., Koster G., Lomonte B., Gutierrez J. M., Montecucco C., Postle A. D. (2013). FEBS J..

[cit22] Mukherjee A. K. (2014). Biochimie.

[cit23] Fernandes C. A., Borges R. J., Lomonte B., Fontes M. R. (2014). Biochim. Biophys. Acta.

[cit24] de Oliveira A. L. N., Lacerda M. T., Ramos M. J., Fernandes P. A. (2024). Toxins.

[cit25] Castro-Amorim J., Novo de Oliveira A., Da Silva S. L., Soares A. M., Mukherjee A. K., Ramos M. J., Fernandes P. A. (2023). J. Med. Chem..

[cit26] Gutierrez J. M., Ownby C. L. (2003). Toxicon.

[cit27] Bitar L., Jundi D., Rima M., Al Alam J., Sabatier J.-M., Fajloun Z. (2021). Venom. Toxins.

[cit28] Jimenez-Charris E., Montoya-Gomez A., Torres J. K., Gomez-Diaz M., Bolivar-Garcia W. (2022). Biochimie.

[cit29] Sasa M., Wasko D. K., Lamar W. W. (2009). Toxicon.

[cit30] Mora-Obando D., Salazar-Valenzuela D., Pla D., Lomonte B., Guerrero-Vargas J. A., Ayerbe S., Gibbs H. L., Calvete J. J. (2020). J. Proteomics.

[cit31] Picado Twight C. , Serpientes venenosas de Costa Rica: sus venenos seroterapia anti-ofídica, CR Alsina, 1931

[cit32] Bolaños R. , Editorial Universidad de Costa Rica, 1984

[cit33] Scott N. , Costa Rican Natural History, The University of Chicago Press, Chicago and London, 1983, pp. 383–384

[cit34] Mora-Obando D., Fernandez J., Montecucco C., Gutierrez J. M., Lomonte B. (2014). PLoS One.

[cit35] Sekar K., Yu B. Z., Rogers J., Lutton J., Liu X., Chen X., Tsai M. D., Jain M. K., Sundaralingam M. (1997). Biochemistry.

[cit36] Caccin P., Pellegatti P., Fernandez J., Vono M., Cintra-Francischinelli M., Lomonte B., Gutierrez J. M., Di Virgilio F., Montecucco C. (2013). Biochem. Biophys. Res. Commun..

[cit37] Doley R., King G. F., Mukherjee A. K. (2004). Arch. Biochem. Biophys..

[cit38] Saikia D., Bordoloi N. K., Chattopadhyay P., Choklingam S., Ghosh S. S., Mukherjee A. K. (2012). Biochim. Biophys. Acta.

[cit39] Burke J. E., Dennis E. A. (2009). J. Lipid Res..

[cit40] Peggion C., Tonello F. (2021). Toxins.

[cit41] Kini R. M. (2003). Toxicon.

[cit42] Burke J. E., Dennis E. A. (2009). Cardiovasc. Drugs Ther..

[cit43] Scott D. L., White S. P., Otwinowski Z., Yuan W., Gelb M. H., Sigler P. B. (1990). Science.

[cit44] Kim R. R., Chen Z., Mann T. J., Bastard K., K F. S., Church W. B. (2020). Molecules.

[cit45] Kang T. S., Georgieva D., Genov N., Murakami M. T., Sinha M., Kumar R. P., Kaur P., Kumar S., Dey S., Sharma S., Vrielink A., Betzel C., Takeda S., Arni R. K., Singh T. P., Kini R. M. (2011). FEBS J..

[cit46] Borges R. J., Salvador G. H. M., Campanelli H. B., Pimenta D. C., de Oliveira Neto M., Uson I., Fontes M. R. M. (2021). Int. J. Biol. Macromol..

[cit47] Berg O. G., Gelb M. H., Tsai M.-D., Jain M. K. (2001). Chem. Rev..

[cit48] Pinto A. V., Ferreira P., Cunha A. V., Havenith R. W. A., Magalhaes A. L., Ramos M. J., Fernandes P. A. (2024). Chem. Sci..

[cit49] Snitko Y., Koduri R. S., Han S. K., Othman R., Baker S. F., Molini B. J., Wilton D. C., Gelb M. H., Cho W. (1997). Biochemistry.

[cit50] Han S. K., Yoon E. T., Scott D. L., Sigler P. B., Cho W. (1997). J. Biol. Chem..

[cit51] Saikia D., Thakur R., Mukherjee A. K. (2011). Toxicon.

[cit52] Mora-Obando D., Diaz C., Angulo Y., Gutierrez J. M., Lomonte B. (2014). PeerJ.

[cit53] Zambelli V. O., Picolo G., Fernandes C. A. H., Fontes M. R. M., Cury Y. (2017). Toxins.

[cit54] Thunnissen M. M., Ab E., Kalk K. H., Drenth J., Dijkstra B. W., Kuipers O. P., Dijkman R., de Haas G. H., Verheij H. M. (1990). Nature.

[cit55] Sekar K., Eswaramoorthy S., Jain M. K., Sundaralingam M. (1997). Biochemistry.

[cit56] White S. P., Scott D. L., Otwinowski Z., Gelb M. H., Sigler P. B. (1990). Science.

[cit57] Scott D. L., White S. P., Browning J. L., Rosa J. J., Gelb M. H., Sigler P. B. (1991). Science.

[cit58] Scott D. L., Otwinowski Z., Gelb M. H., Sigler P. B. (1990). Science.

[cit59] Verheij H. M., Volwerk J. J., Jansen E. H., Puyk W. C., Dijkstra B. W., Drenth J., de Haas G. H. (1980). Biochemistry.

[cit60] Van Den Bergh C. J., Slotboom A. J., Verheij H. M., De Haas G. H. (1988). Eur. J. Biochem..

[cit61] Maraganore J. M., Merutka G., Cho W., Welches W., Kezdy F. J., Heinrikson R. L. (1984). J. Biol. Chem..

[cit62] Diaz-Oreiro C., Gutierrez J. M. (1997). Toxicon.

[cit63] Li Y., Yu B. Z., Zhu H., Jain M. K., Tsai M. D. (1994). Biochemistry.

[cit64] Betzel C. , Singh T. P., Georgieva D. and Genov N., in Handbook of Metalloproteins, ed. A. Messerschmidt, R. Huber, T. Poulas, K. Wieghardt, M. Cygler and W. Bode, Wiley, 2004

[cit65] Verheij H., Slotboom A., De Haas G. (1981). Rev. Physiol. Biochem..

[cit66] Peters A. R., Dekker N., van den Berg L., Boelens R., Kaptein R., Slotboom A. J., de Haas G. H. (1992). Biochemistry.

[cit67] Dennis E. A., Cao J., Hsu Y. H., Magrioti V., Kokotos G. (2011). Chem. Rev..

[cit68] Yu B.-Z., Rogers J., Nicol G. R., Theopold K. H., Seshadri K., Vishweshwara S., Jain M. K. (1998). Biochemistry.

[cit69] Epstein T. M., Yu B. Z., Pan Y. H., Tutton S. P., Maliwal B. P., Jain M. K., Bahnson B. J. (2001). Biochemistry.

[cit70] Jambeck J. P., Lyubartsev A. P. (2013). J. Chem. Theory Comput..

[cit71] Jambeck J. P., Lyubartsev A. P. (2012). J. Phys. Chem. B.

[cit72] Krack M. (2005). Theor. Chem. Acc..

[cit73] Lee B. I., Dua R., Cho W. (1999). Biochemistry.

[cit74] Gudmand M., Rocha S., Hatzakis N. S., Peneva K., Müllen K., Stamou D., Uji H., Hofkens J., Bjørnholm T., Heimburg T. (2010). Biophys. J..

[cit75] Lomize A. L., Pogozheva I. D., Lomize M. A., Mosberg H. I. (2007). BMC Struct. Biol..

[cit76] Pogorelov T. V., Vermaas J. V., Arcario M. J., Tajkhorshid E. (2014). J. Phys. Chem. B.

[cit77] Qin S., Pande A. H., Nemec K. N., Tatulian S. A. (2004). J. Mol. Biol..

[cit78] Yu B. Z., Rogers J., Tsai M. D., Pidgeon C., Jain M. K. (1999). Biochemistry.

[cit79] Yang C. C., Chang L. S. (1988). Toxicon.

[cit80] Qin S., Pande A. H., Nemec K. N., He X., Tatulian S. A. (2005). J. Biol. Chem..

[cit81] Stahelin R. V., Cho W. (2001). Biochemistry.

[cit82] Liu X., Zhu H., Huang B., Rogers J., Yu B. Z., Kumar A., Jain M. K., Sundaralingam M., Tsai M. D. (1995). Biochemistry.

[cit83] Bezzine S., Bollinger J. G., Singer A. G., Veatch S. L., Keller S. L., Gelb M. H. (2002). J. Biol. Chem..

[cit84] Baker S. F., Othman R., Wilton D. C. (1998). Biochemistry.

[cit85] Sousa S. F., Calixto A. R., Ferreira P., Ramos M. J., Lim C., Fernandes P. A. (2020). ACS Catal..

[cit86] Beers S. A., Buckland A. G., Giles N., Gelb M. H., Wilton D. C. (2003). Biochemistry.

[cit87] Sumandea M., Das S., Sumandea C., Cho W. (1999). Biochemistry.

[cit88] Diraviyam K., Murray D. (2006). Biochemistry.

[cit89] Snitko Y., Han S. K., Lee B. I., Cho W. (1999). Biochemistry.

[cit90] Diaz C., Leon G., Rucavado A., Rojas N., Schroit A. J., Gutierrez J. M. (2001). Arch. Biochem. Biophys..

[cit91] Buckland A. G., Wilton D. C. (2000). Biochim. Biophys. Acta.

[cit92] Lucas K. K., Dennis E. A. (2005). Prostaglandins Other Lipid Mediators.

[cit93] Wang Y., Zhang J., Zhang D., Xiao H., Xiong S., Huang C. (2018). Molecules.

[cit94] Salvador G. H. M., Borges R. J., Lomonte B., Lewin M. R., Fontes M. R. M. (2021). Biochim. Biophys. Acta Gen. Subj..

[cit95] Bryan-Quiros W., Fernandez J., Gutierrez J. M., Lewin M. R., Lomonte B. (2019). Toxicon.

[cit96] Salvador G. H. M., Gomes A. A. S., Bryan-Quiros W., Fernandez J., Lewin M. R., Gutierrez J. M., Lomonte B., Fontes M. R. M. (2019). Sci. Rep..

