# Supplementary material for: Beyond Fang's fury: a computational study of the enzyme–membrane interaction and catalytic pathway of the snake venom phospholipase A2 toxin
Source: Chem Sci. 2025 Jan 2;16(4):1974–85. doi: 10.1039/d4sc06511e (PMC11694569; doi:10.1039/d4sc06511e)
Supplement: SC-016-D4SC06511E-s002 [file SC-016-D4SC06511E-s002.pdf]

Supplementary Information (SI) for Chemical Science.
This journal is © The Royal Society of Chemistry 2024

Supporting Information

Beyond Fang's Fury: A Computational Study of the Enzyme-Membrane Interaction and Catalytic Pathway of the Snake Venom Phospholipase A_2 Toxin

Juliana Castro-Amorim, [a] Alexandre V. Pinto, [a] Ashis K. Mukherjee, [b] Maria J. Ramos [a] and Pedro A. Fernandes, *[a]

[a] LAQV, REQUIMTE, Departamento de Química e Bioquímica, Faculdade de Ciências, Universidade do Porto, Rua do Campo Alegre, s/n, 4169-007 Porto, Portugal

[b] Institute of Advanced Study in Science and Technology, Vigyan Path Garchuk, Paschim Boragaon, Guwahati-781035, Assam, India

*e-mail: pafernan@fc.up.pt

Section I – Introduction

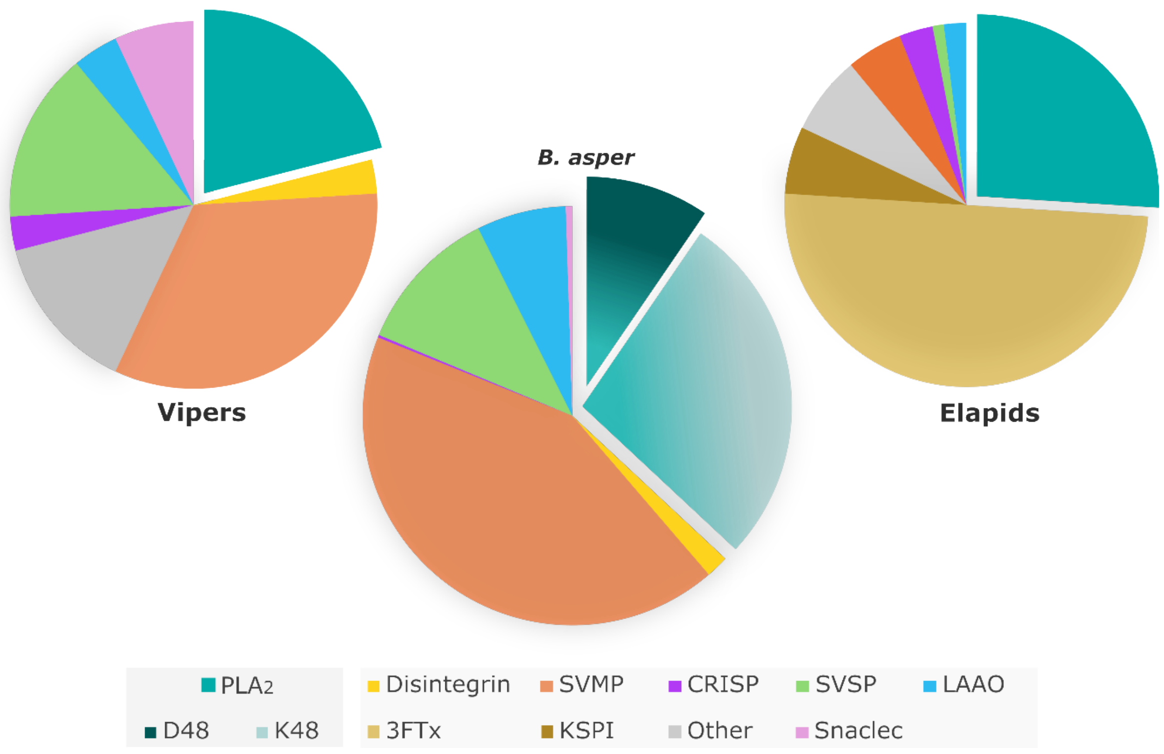

Figure S1: The averaged abundance of the major toxin classes present in the venom proteome of the Viperidae (left) and Elapidae (right) families [1, 2] and in the adult bothropic venom [3]: Phospholipases A_2 (PLA_2) of the D48 or K48 subtypes (PLA_2 D48 or K48); Disintegrin; Zn^{2+}-dependent metalloproteinase (SVMP); Cysteine-rich secretory protein (CRISP); Snake venom serine proteinase (SVSP); ʟ-amino acid oxidase (LAAO); Snake C-type lectin (Snaclec); Three-finger toxins (3FTx); Kunitz-type serine protease inhibitor (KSPI).

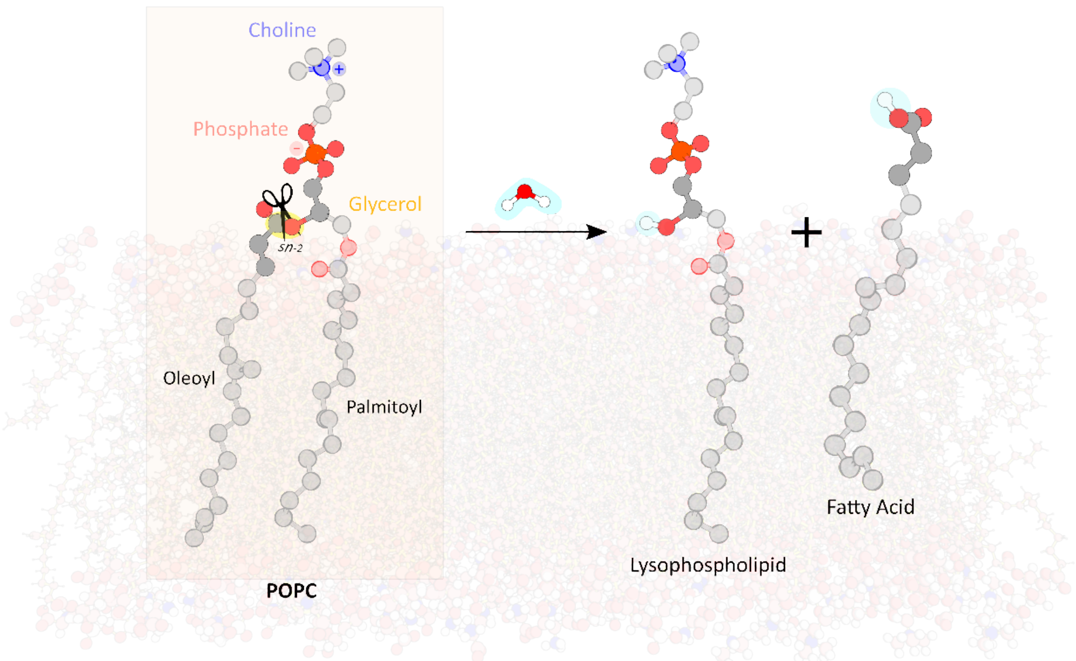

Figure S2: The chemical reaction catalyzed by PLA_2s. These enzymes act at the sn-2 position (yellow shadow) of glycerophospholipids, in this case, a 1-palmitoyl-2-oleoyl-sn-glycero-3-phosphocholine (POPC) phospholipid and hydrolyze the ester bond releasing a lysophospholipid and a free fatty acid. The components constituting the POPC lipid molecule (phosphatidylcholine headgroup, glycerol backbone, and the two hydrophobic tails) are also depicted.

Section II – Materials and Methods

Model Construction

We used the X-ray crystallographic structure of the svPLA2 Mt-I from B.asper obtained from the Protein Data Bank (PDB code: 5TFV) [4] to build the computational model. However, the Ca^{2+}-binding loop in the X-ray structure was distorted, and hence, we used the Modeller 9.11v software (San Francisco, CA, USA) to model this region using the Indian saw-scaled viper (Echis carinatus) acidic PLA2 structure (PDB ID: 1OZ6 [5]) as a template. This structure exhibits a homodimeric conformation and lacks mutations and ligands, including the Ca2+ ion. In the present study, we used the monomeric form for simplicity to gain a fundamental understanding of the enzyme's core catalytic machinery and, subsequently, its potential mechanistic pathways. Its sequence and respective secondary structure are shown in **Figure S3**.

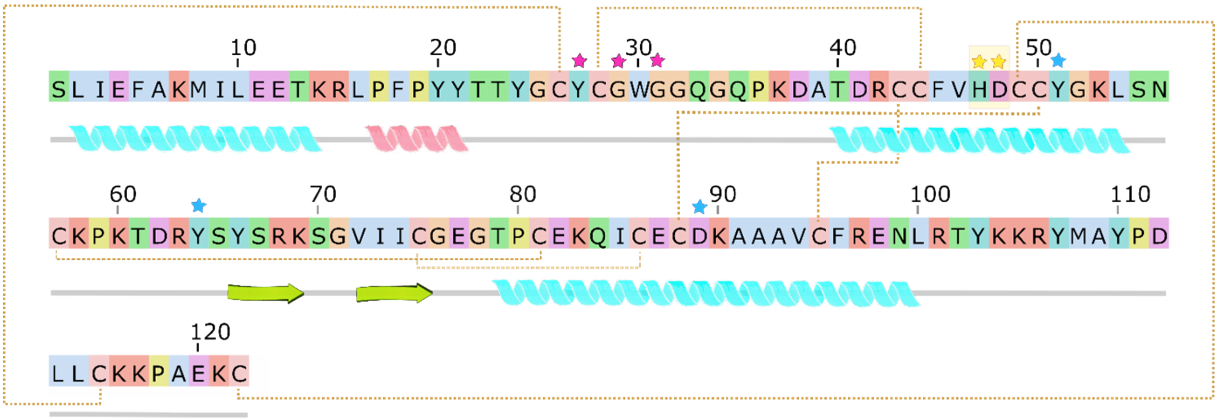

Figure S3: Secondary structure of the svPLA2 MT-I (UniProtKB AC: P20474) amino acid sequence: α-helices (cyan helix); shorter helix (pink helix); β-sheets (green arrows); loops (grey). Active site residues are marked with the symbol star: Ca^{2+} binding loop residues (pink stars), His48 and Asp 49 (yellow stars), and Tyr51, Asp 89, Tyr64 (blue stars). Disulfide bonds are represented by dashed yellow lines. Amino acid residues are colored according to the ClustalX color scheme: hydrophobic (blue), positive charge (red), negative charge (magenta), polar (green), cysteines (pink), glycines (orange), prolines (yellow), aromatic (cyan), and unconserved (white).

We manually incorporated the productively-bound substrate molecule, specifically a 1-palmitoyl-2-oleoyl-sn-glycero-3-phosphocholine (POPC) phospholipid, into the active site of Mt-I. The modeling of the substrate POPC on the active site took into account structural data derived from several svPLA2-inhibitor complexes accessible in the Protein Data Bank (PDB codes: 1POB [6], 1POE [7], 1POC [8], and 5P2P [9]). In addition, essential interactions critical for the enzymatic reaction were incorporated, encompassing the coordination of the Ca^{2+} cofactor by a negative phosphate oxygen and the carbonyl group of the *sn*-2 chain of the phospholipid substrate, as well as the involvement of the two carboxylate oxygens of the Asp48 sidechain and the backbone carbonyl oxygen atoms from the Ca^{2+}-binding loop.

The Avogadro software [10] facilitated this process, followed by successive geometry optimizations using the GAFF2 force field while keeping the protein structure fixed. We used the PROPKA 3.0 package [11, 12] integrated into the PDB2PQR server to compute the pKa values of the titratable residues and assign their protonation states at physiological pH (Supporting **Table S1**). Considering the pKa predictions, the chemical environment, and the reaction mechanism, all residues were predicted to be in their typical protonation states. Glu12, Asp48, and Asp89 had higher than usual sidechain pKa values. However, they established hydrogen bonds and electrostatic interactions with the surrounding environment.

pKa values of the protein titratable residues at pH=7

Table S1: pKa values of the protein titratable residues at pH=7 predicted by PROPKA 3.0 integrated in the PDB2PQR server.

Residue	pKa	Residue	pKa	Residue	pKa
Lys 7	10.89	Asp 38	3.77	Cys 122	99.99
Lys 14	10.51	Asp 41	4.97	Tyr 20	9.96
Lys 37	11.37	Asp 48	5.85	Tyr 21	12.58
Lys 53	10.53	Asp 62	3.27	Tyr 24	13.47
Lys 58	10.49	Asp 89	6.68	Tyr 27	12.59
Lys 60	10.15	Asp 112	4.00	Tyr 51	14.92
Lys 69	10.42	Glu 4	4.18	Tyr 64	15.36
Lys 83	10.51	Glu 11	4.11	Tyr 66	10.64
Lys 90	10.35	Glu 12	6.51	Tyr 103	12.07
Lys 104	10.62	Glu 77	4.65	Tyr 107	9.99
Lys 105	10.32	Glu 82	5.15	Tyr 110	9.48
Lys 116	10.18	Glu 87	4.59		
Lys 117	10.14	Glu 98	4.73		
Lys 121	10.46	Glu 120	4.27		
Arg 15	12.73				
Arg 42	12.33				
Arg 63	12.61				
Arg 68	12.40				
Arg 97	12.54				
Arg 101	12.45				

svPLA$_2$:POPC:membrane complex modelling

The lipid bilayer was prepared using the composition described in the study by [13], for consistency and comparability between the non-toxic human PLA2 and the highly toxic svPLA$_2$. The study explored the human sPLA$_2$ enzyme and tested several membrane compositions, specifically 1:0, 9:1, 4:1, 2:1, 3:2, and 1:1 POPC/POPS. The 1:1 membrane represents a compromise between the greater affinity for pure POPS and the scarcity of POPS in the outer leaflet of myocytes (10-15%), the Mt-I primary target. As POPS is not homogeneously distributed, there are regions rich in POPS. Thus, a 1:1 POPC/POPS membrane was selected to compromise these two factors. In addition, it permits direct comparison with the studies made with the human PLA$_2$. We used the CHARMM-GUI *Membrane builder* module (http://www.charmm-gui.org) to generate the lipid bilayer [14, 15]. The target structure was positioned at the upper leaflet of a bilayer composed of 1-palmitoyl-2-oleoyl-sn-glycero-3-phosphocholine (POPC) and 1-palmitoyl-2-oleoyl-sn-glycero-3-phosphoserine (POPS) lipids in a 1:1 ratio, which resulted in a tilt angle of 79° (≈ experimental value of 76°). The system comprised 160 lipid molecules, symmetrically distributed in each leaflet (80 POPC: 80 POPS per leaflet). We immersed this entire complex within a 72 × 72 × 100 Å^3 rectangular box consisting of 26,946 TIP3P water molecules. We removed those overlapping with the membrane, and total charge neutrality was achieved by randomly substituting water molecules with sodium (96 Na^+) and chloride (25 Cl^-) counter ions to reach the physiological salt concentration (150 mM NaCl) (**Figure S4**). The protein was parameterized using the AMBER99SB-ildn force field, while the lipids were characterized with Slipids-2020 [16, 17] which has been shown to yield ensembles that closely resemble experimental observations [18]. We used the TIP3P [19] water model to describe the water molecules, the Joung–Chetham parameters [20] for Na^+ and Cl^-, and the Li-Merz parameters [21] to characterize the catalytic Ca^{2+} ion. This setup resulted in a final system comprising 49,863 atoms.

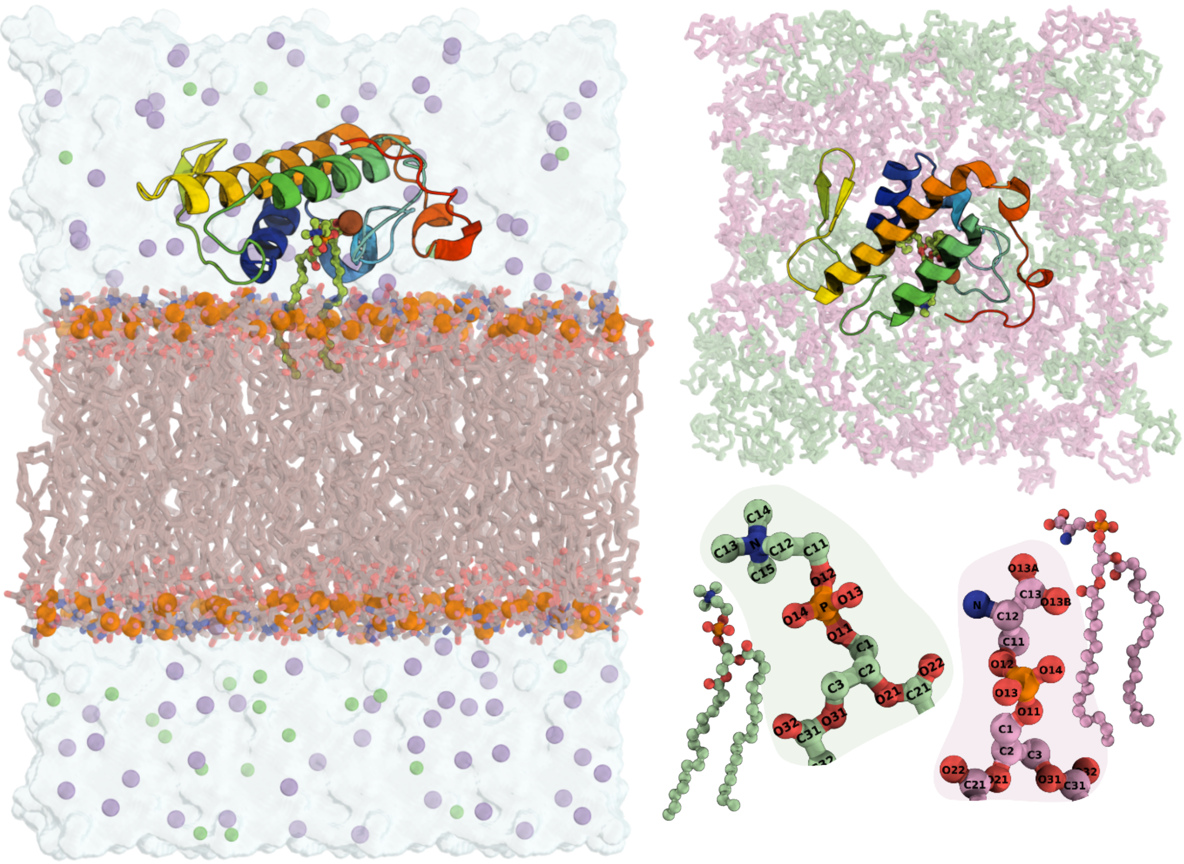

Figure S4: The modeled protein-membrane system; **Left:** the protein structure depicted in rainbow cartoon and the POPC substrate in lime green sticks. A light blue surface represents the solvent environment, while chloride (Cl^-) and sodium (Na^+) ions are denoted by green and purple spheres. The lipid membrane is depicted in brown sticks, emphasizing the phosphate groups as orange spheres. **Top right:** top view of the protein-membrane system; the distribution of the phospholipids is highlighted, with POPC and POPS depicted in green and pink sticks; **Bottom right:** close-up view of the head and glycerol ester groups of POPC and POPS.

Classical Molecular Dynamics (cMD) Simulations

We performed MD simulations using the GROMACS 2021.5 software package (https://manual.gromacs.org/) [22].
We used the leapfrog MD algorithm [23] with an integration timestep of 2 fs. All intramolecular movements associated with hydrogen bonds were constrained using the LINCS algorithm [24]. We chose the Particle-Mesh Ewald (PME) scheme with an order of 4 and a 1.25 Å Fourier-spacing [25] for the calculation of long-range electrostatic interactions beyond the non-bonded interaction cutoff of 10 Å and truncated the Lennard-Jones interactions. For neighbor searching, the Verlet scheme was settled. All simulations ran under periodic boundary conditions (PBC) in all directions. The target temperature was maintained at 310.15 K using the Nosé–Hoover thermostat [26] with a coupling constant of 0.5 ps. Two groups were coupled independently: the first included the holo-protein, while the second included the remaining system. The pressure was kept constant at 1.0 bar using the Parrinello-Rahman barostat [27] with a semi-isotropic scheme (i.e., isotropic in the XY plane) and a coupling constant of 0.5 ps.
The system underwent two energy-minimization steps before going to MD simulations, both of which included freezing the protein, calcium, and the bound substrate with a harmonic restraint of force constant of 1000 $kJ\cdot mol^{-1}\cdot nm^{-2}$: i) in the

first step, 100 steps of the steepest-descent minimization with softcore potentials were applied to avoid bad contacts between the membrane and the complex, thereby avoiding unrealistic oscillations and achieving a stable energy minimization process; ii) in the second step, another 50000 steps of the steepest-descent minimization were carried out using regular Lennard-Jones potentials for standard energy minimization. The MD simulation followed a two-stage process for six replicas (REP1-6). First, the protein, substrate, and calcium ion were frozen while the membrane was gradually equilibrated using a series of canonical (NVT) and isothermal isobaric (NPT) simulations. This allowed the membrane to adapt and stabilize. In the second stage, the protein was structurally relaxed through a 4-round minimization process and progressively reducing the imposed positional harmonic restraints. Harmonic potentials were then used to refine key catalytic distances and angles for 49 ns in the NPT ensemble.

Finally, we carried out a production run of 250 ns with six replicas (REP 1-6) to enhance sampling further, enabling the entire system to evolve freely. The detailed description of the MD protocol can be found in **Supporting Table S2**.

Table S2: Molecular Dynamics detailed protocol integrating rigorous energy minimization, meticulous equilibration, and extensive production phases, ensuring the robust exploration of sPLA$_2$:POPC:membrane complex dynamics.

			**Convergence criteria (emtol)**	**Frozen groups (freezegrps)**	**Positional restraints**	**Simulation time**
**MEMBRANE EQUILIBRATION**	Minimization	**Softcore potentials**	1000.0 kJ.mol^{-1}	Protein<br>Ca^{2+}<br>POPC substrate	-	1 ps
		**Regular Lennard Jones**	1000.0 kJ.mol^{-1}		-	500 ps
	Equilibration	**NVT**<br>V-rescale Thermostat	-		1000 kJ.mol^{-1}<br>On lipids (POPC and POPS)	50 ps
			-		-	150 ps
		**NPT**<br>Berendsen Thermostat and Barostat	-	-	1000 kJ.mol^{-1}<br>On the protein, calcium and POPC substrate	1 ns
		**NPT**<br>Nose-Hoover Thermostat<br>Parrinello-Rahman Barostat	-	-		
	Production	**NVT**<br>Nose-Hoover Thermostat	-	Protein<br>Ca^{2+}<br>POPC substrate	-	200 ns
**SYSTEM EQUILIBRATION**	Minimization for frozen groups	**Regular Lennard Jones**	1000.0 kJ.mol^{-1}	Protein and not hydrogen atoms<br>Ca^{2+}<br>POPC substrate	-	2 ns
				Protein main chain<br>Ca^{2+}<br>POPC substrate	-	
				Protein Backbone<br>Ca^{2+}<br>POPC substrate	-	
				-	-	
	Equilibration	**NPT**<br>Berendsen Thermostat and Barostat	-	.	500 kJ.mol^{-1}<br>On the protein, calcium and POPC substrate	1 ns
		**NPT**<br>Nose-Hoover Thermostat<br>Parrinello-Rahman Barostat	-	-	250 kJ.mol^{-1}<br>On the protein, calcium and POPC substrate	
			-	-	Harmonic potentials on the catalytic residues	49 ns
	Production		-	-	-	250 ns

Clustering Analysis

Upon careful analysis of the six MD replicas, it became clear that, although the fifth replica (REP5) exhibits the highest level of stability and suitability for the reaction mechanism, it was challenging to select a snapshot that preserved the catalytic interatomic distances.

Therefore, we performed two clustering processes to address this issue and to identify near-reactive conformations of the svPLA$_2$:POPC:membrane complex. The first process involved a homemade script designed to assemble structures that followed pre-determined interatomic distance criteria. The trajectory was divided into clusters based on certain

interatomic distances. In this case, Ca^{2+}–Tyr27 ≤ 2.9 Å, Ca^{2+}–Gly29 ≤ 2.9 Å, Ca^{2+}–Gly29≤ 2.9 Å, Ca^{2+}–Asp_{48} $O^{\delta2}$ ≤ 3.0 Å, Ca^{2+}–CO_{popc} ≤ 2.9 Å, His_{47} N^{δ}–$O31_{popc}$ ≤ 5.0 Å and Tyr_{51} OH–Asp_{89} $O^{\delta1}$ ≤ 2.9 Å. These specific distances ensure that the coordination of the labile Ca^{2+} cofactor is complete and that the catalytic interactions are formed. The representative structure of the dominant cluster was used for further studies. This selection also considered quite specific criteria: the position of the water molecule or molecules regarding the His47 N^{δ} atom and the substrate's carbonyl carbon (C_{popc}) in the representative structure, the simulation's stage, and its conformational representativeness. The conformations resulting from this clustering step (≈ 20 % of the full trajectory), were further organized into distinct clusters (Figure S5A) based on the RMSD of residues within 6 Å of the substrate POPC (Figure S5B). To this end, the GROMOS clustering method was used to explore structural heterogeneity in the structures generated in the previous step. This would enable a more thorough exploration of the conformational landscape of the retrieved trajectory. It is noteworthy that the first 60 ns of the simulation were left out of the analysis to analyze a more stable stage of the enzyme's structural dynamics.

To select an adequate $C\alpha$ RMSD cut-off and capture structural diversity among the retrieved frames, its values were modified between 0.6 and 1.2 Å in steps of 0.05 Å and for each one a clustering analysis was performed (Figure S5B). The topology and coordinates files of the selected snapshot were processed by the Parmed Python module from the AmberTools 18 package [28]. The phospholipids were then re-parameterized using the Lipids17 force field. This substitution was made because the short duration of the following simulations, on the order of picoseconds, would not lead to significant conformational changes.

The superimposition of representative structures from each of the five clusters is displayed in Figure S6.

A

	Ca-Tyr28	Ca-Gly30	Ca-Gly32	Ca-Asp49OD2	Ca-Asp49OD1	Ca-His48	His48-O31popc	Asp89-His	Asp89OD2-Tyr64	Ca-PO	Ca-Carbonyl	Asp89-Tyr51
**MAX**	0.361	0.356	0.534	0.413	0.470	0.679	0.629	0.344	0.274	0.282	0.444	1.001
**MIN**	0.243	0.243	0.242	0.238	0.237	0.438	0.311	0.157	0.140	0.234	0.241	0.145
**AVERAGE**	0.273	0.271	0.269	0.265	0.265	0.514	0.458	0.188	0.176	0.253	0.272	0.420
**STDEV**	0.011	0.011	0.012	0.010	0.011	0.026	0.052	0.016	0.014	0.006	0.013	0.188

Coordinates	Distances	Frames	%
➡ Ca-Tyr28	2.9 > Dist > 2.4	5775	0.924
➡ Ca-Gly30	2.9 > Dist > 2.4	5931	0.949
➡ Ca-Gly32	2.9 > Dist > 2.4	6070	0.971
➡ Ca-D49OD2	3.0 > Dist > 2.3	6139	0.982
Ca-D49OD1	3.0 > Dist > 2.3	6211	0.994
Ca-His48	6.0 > Dist > 4.0	6204	0.992
Ca-PO	2.9 > Dist > 2.3	6251	1.000
➡ Ca-CO	2.9 > Dist > 2.4	5800	0.928
➡ His48-O31popc	5.0 > Dist > 3.0	4915	0.786
➡ Asp89-Tyr51	2.9 > Dist > 1.4	1721	0.275
Asp89-Tyr64	2.9 > Dist > 1.4	6251	1.000
Asp89-His	2.9 > Dist > 1.4	6237	0.998

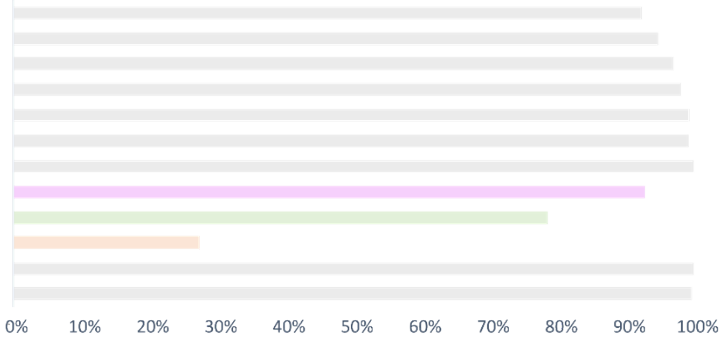

B

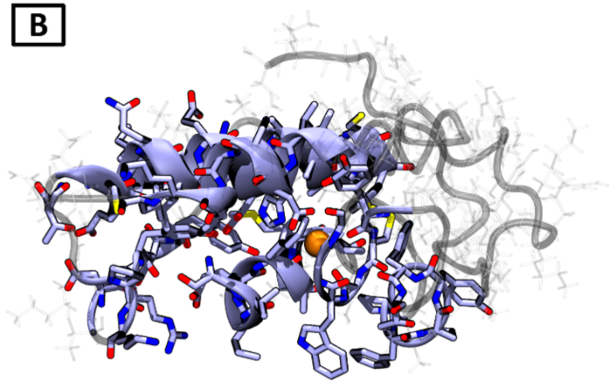

RMSD cutoff	N clusters
0.060	190
0.065	178
0.070	138
0.075	97
0.080	58
0.085	36
0.090	22
0.095	16
0.100	9
0.105	7
0.110	5
0.115	4
0.120	3

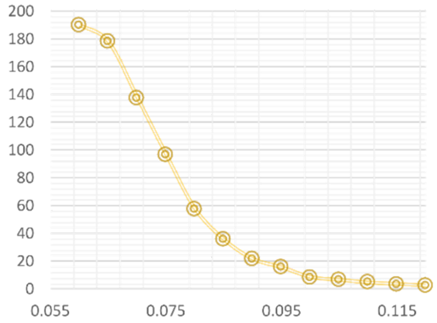

Figure S5: Structure Analysis for the first clustering process of the REP5 trajectory. Preceding the implementation of a custom script for the initial clustering process, an exhaustive evaluation of interatomic distances vital to the reaction mechanism was conducted using the GROMACS tool, gmx mindist. A) The average, standard deviation, maximum and minimum values were calculated. Those distances were then organized between certain thresholds to access how many frames would obey specific distance criteria. Those thresholds were also used for the making of the script; B) Selection for the second clustering process. Cartoon and stick representation of the selection used for the second clustering process, focusing on residues within 6 Å of residue 124 and residue Asp89 (colored in ice-blue color). The associated plot features the impact of different Cα RMSD cutoffs (ranging from 0.060 to 0.120) on the resultant number of clusters. Pale orange highlights the chosen cutoff of 0.110 nm, which yielded five clusters. Notably, the dominant cluster within the chosen cutoff encompasses approximately 75% of structures

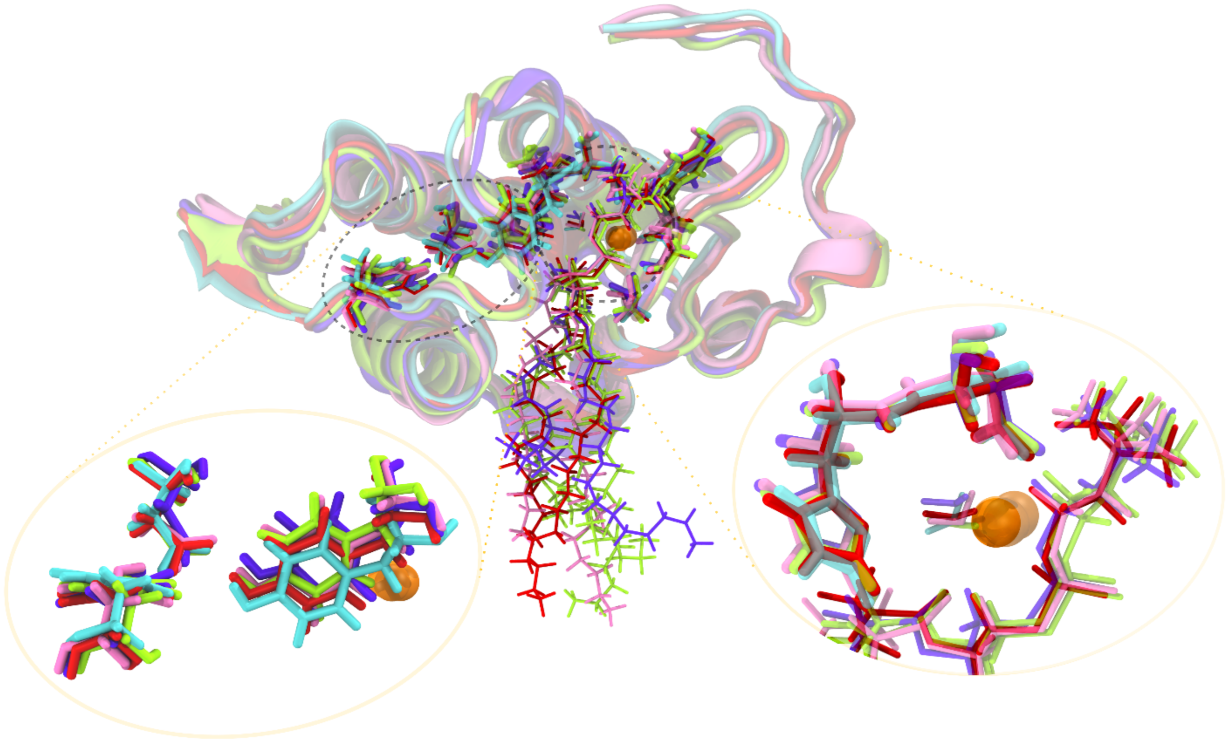

Figure S6 - Superimposition of the representative structures from each of the five clusters: representative structure of the first cluster (red color), second cluster (pink color), third cluster (purple color), fourth cluster (light green color) and fifth cluster (cyan color). (left) Close-up view of the Tyr64, Asp89 and Tyr51 conformations; (Right) Close-up view of the His48, Asp49, POPC substrate, Ca^{2+} cofactor and catalytic water molecules conformations.

QM/MM MD Simulations

We carried out all QM/MM MD simulations with the CP2K software. In this setup, the QUICKSTEP [29] and FIST modules were used to compute the interaction forces within the QM and MM layers, respectively.

We chose the Perdew-Burke-Ernzerhof (PBE) [30] functional using the dual Gaussian plane-wave (GPW) [31] formalism, to treat the QM layer because it results in a good compromise between accuracy and computational time. The electron density was described by a Gaussian double-ζ valence polarized (DZVP) basis set and an auxiliary plane-wave cutoff of 300 Ry, while core electrons were approximated by Geodecker–Teter–Hutter (GTH-PBE) pseudopotentials [32].

The QM unit cell size defined as 33.23 Å × 24.20 Å × 27.43 Å comprised a total of 167 atoms with a neutral charge and a singlet spin multiplicity (**Figure S7**). On the other hand, the MM layer, which represented the remaining portion of the system, was treated with the same force field as that used in classical MD simulations.

Hydrogen atoms, *link atoms*, completed valences at the boundary region implemented in the IMOMM scheme [33]. Long-range Coulomb interactions were approximated by the Gaussian expansion of the electrostatic potential method (GEEP) [34] and we employed the smooth particle mesh Ewald (SPME) method [35] to compute electrostatic interactions. The scaling factors for 1-4 electrostatic and LJ interactions were scaled by a factor of 0.8333 and 0.5, respectively.

We got the LBFGS algorithm to sequentially optimize the system's geometry utilizing the mechanical and electrostatic embedding schemes. The optimized structure was then equilibrated in the NVT ensemble, setting the canonical sampling

through velocity rescaling (CSVR) thermostat [36] at 310.15 K for two ps and with a time-step of 1 fs. We used the CPPTRAJ algorithm of the AMBER 18 software [28] package to analyze the trajectories.

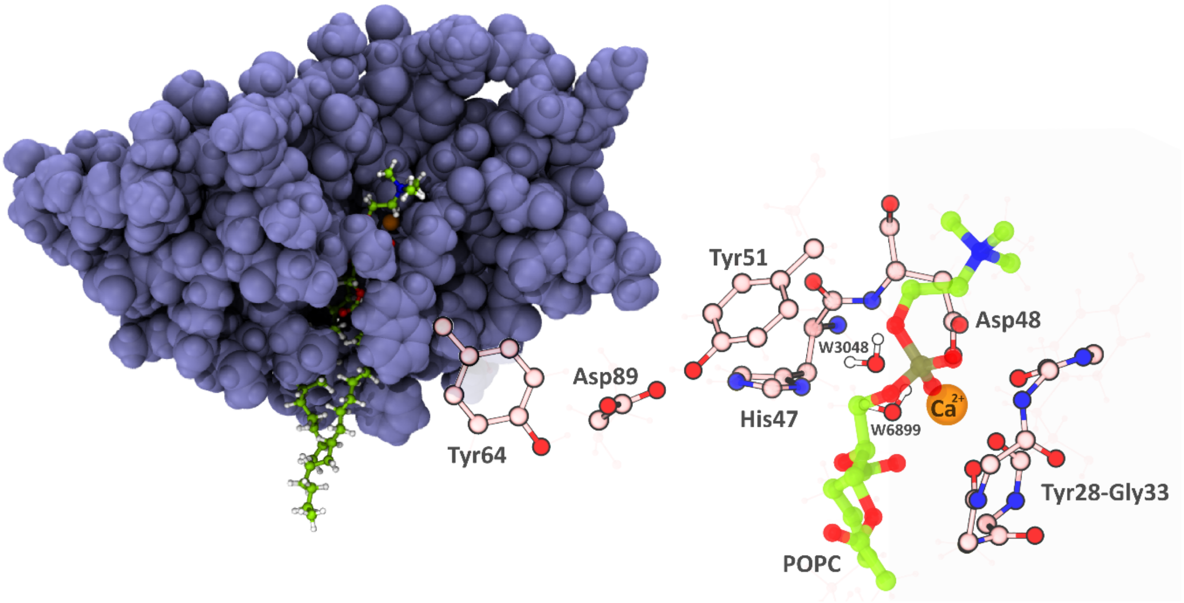

Figure S7: Left: Representation of the BaMt-I:POPC complex; **Right:** close-up view of the QM model used in the calculations. Protein residues are shown in pink sticks, the Ca^{2+} cofactor as an orange sphere, the substrate as green sticks, and the water molecules as CPK. Carbon is shown in pink, nitrogen in blue, oxygen in red, and hydrogen in white.

Steered Molecular Dynamics (SMD)

We conducted two rounds of steered molecular dynamics (SMD) QM/MM MD of 5 ps each, with an integration time-step of 1 fs, for the single-water mechanism. Additionally, we described the reaction coordinates using two collective variables (CVs), CV1 = (d_1 + d_2) and CV2 = (d_3 + d_4), calculated by adding the values of the distances**.** An external-harmonic force constant of 200.0 kcal·mol^{-1} ·Å^{-2} and a target growth of ≈ 0.0007 Å·fs^{-1} was applied. We followed the same procedure for a second replica, in which the presence of a harmonic potential between Ca^{2+} and the carbonyl oxygen of the substrate POPC in the first one, was the sole distinction from the first replica.

CV1 represents the water proton transfer to the catalytic His47 (d_1 = H_{wat} - His_{47} N^{δ}) and the subsequent nucleophilic attack on the carbonyl carbon of the substrate (d_2 = O_{Wat} - C_{popc}). These distances were explored from their initial sum value (~ 5 Å) to the desired final sum value of 2 Å. This marks the first step of the reaction, effectively narrowing the distances. CV2 represents the break of the substrate C-O bond (d_3 = C_{popc}-O_{popc}) and the proton transfer from the His47 to the substrate oxygen by stretching the distance between the His47 proton and the water's oxygen (d_4 = His_{47} $N^{\delta H+}$-O_{wat}). These distances were explored from their initial sum value (~ 3.5 Å) to the desired final sum value of 7.5 Å, increasing the distances.

The same approach was used for the assisted-water mechanism. We employed two CVs, CV1 = d_1 + d_2+ d_3 and CV2 = d_4 - d_5, to describe the reaction coordinates**,** and an external-harmonic force constant of 200.0 kcal·mol^{-1} ·Å^{-2}, with a target growth of -0.00119976 for CV1 and 0.000737852 Å·fs^{-1} for CV2.

CV1 represents simultaneously the deprotonation of the catalytic water (Wnuc) by the bridging water molecule (Wassist) (d_1 = H_{Wnuc} – $O_{Wassist}$), the deprotonation of the latter by the His47 Nδ1 atom (d_2 = $H_{Wassist}$ - N_{His47}) and the nucleophilic attack by Wnuc on the carbonyl carbon of the substrate (d_3 = O_{Wnuc} - C_{popc}). These distances were explored from their initial sum value of 8.5 Å to the desired final sum value of 2.5 Å, decreasing the distances. CV2 represents both the

cleavage of the substrate's C-O bond ($d_4 = C_{popc}$-O_{popc}) by stretching the bond and the transference of the proton to the substrate's oxygen by the His47 ($d_5 = H_{His47}$-O_{popc}) by shortening it. These distances were explored from their initial sum value (~ -0.6 Å) to the desired final sum value of ~ 3 Å.

Umbrella Sampling (US)

We used the geometries produced along the SMD simulations to model the initial structures of the umbrella sampling (US) [37] simulations.

To compute the free energy profile along the reaction coordinates with umbrella sampling (US), CV1 and CV2 were defined as reaction coordinates (ξ_1 and ξ_2). In the single-water mechanism, ξ1 and ξ2 were spaced at 0.1 Å intervals (Δξ = 0.1 Å), resulting in 80 US windows, half for each ξ. In the assisted-water mechanism, ξ1 and ξ2 were spaced at 0.2 Å intervals (Δξ = 0.2 Å), resulting in 31 and 19 US windows for ξ1 and ξ2, respectively. The starting configuration for each US window was derived from the prior SMD simulations considering those reference values. Additionally, we applied harmonic umbrella biasing potentials to the value of the CVs, centered at the reference values, with a force constant of 50, 75, or 100 $kcal{\cdot}mol^{-1}{\cdot}Å^{-2}$. For each umbrella sampling window, the whole system underwent an equilibration step for 4 ps followed by 16 ps of production with an integration time-step of 1 fs, corresponding to a total simulation time of 20 ps.

To ensure proper overlap between successive windows, additional windows were inserted when density gaps occurred at such intervals, and the process was iteratively repeated until there were no gaps.

We derived the free energy profile by merging all the US windows using the weighted histogram analysis method (WHAM) [38].

Free-energy and Potential of Mean Force (PMF) calculations

To calculate the potential of mean force (PMF), i.e., free energy profile, a dump frequency of 5 fs was used to retrieve the CV values of each window, disregarding the first 2.5 ps as equilibration.

To estimate the energy profile error, a Bayesian bootstrapping analysis (100 bootstraps) was carried out. A convergence tolerance of 0.001 $kcal{\cdot}mol^{-1}$ was used employing a number of bins that doubled the number of independent simulated windows.

The convergence of the profiles was assessed by collecting each umbrella-sampling data and computing the PMF over blocks of 2 ps with their overlap indicating consistency. Convergence was confirmed when the recalculated profiles overlapped, indicating that the results had stabilized and became consistent.

PyMOL (http://www.pymol.org) and VMD v1.9.3 [39] were employed to visualize and render the molecular figures, while MatPlotLib 3.7.5 [40] was used to create the analysis plots. Several Python libraries such as NumPy 1.24.4 [41], Scipy 2.0.0 [42], MDTraj 1.9.5 [43], and MDAnalysis 2.4.3 [44] were also used for trajectory analysis

Section III – Results and Discussion

Analysis of the svPLA2-membrane dynamic behavior

We first analyzed the simulation trajectories to gain insight into the catalytic mechanism of svPLA2s. We further analized the system stability and, hence, the dynamics of the enzyme active site and the substrate POPC over the whole MD trajectory. Moreover, we carried out an in-depth analysis of the root-mean-squared deviation (RMSd), catalytic distances, radius of gyration (Rg), secondary structure, root-mean-square fluctuations (RMSF), radial distribution functions (RDFs) and hydrogen bonds, using GROMACS analytical tools (**Figures S8-15**). The overall behavior observed across the different replicas was quite similar as shown in **Figures S8**-**9**, indicating consistency in the simulation results. However, some exceptions were noted regarding specific distances: Tyr_{51} OH – Asp_{89} $O^{\delta 1}$, His_{47} N^{δ}– C_{popc} and Ca^{2+}– CO_{popc}. Given that REP5 demonstrated greater stability and met all relevant distance criteria for subsequent mechanistic studies, it was selected for further analysis and mechanistic studies.

The bilayer's physical properties, i.e., the variations in the area per lipid (ΔAPL) and membrane thickness (ΔT) along the simulation, were calculated using the FATSLiM (Fast Analysis Toolbox for Simulations of Lipid Membranes) [45] tool (**Figure S14**). Analyses demonstrated that the protein-membrane system was fully equilibrated and stable in a catalytic conformation. The calculated structural properties were consistent with experimental measurements, confirming the accuracy and reliability of the modelled system.

Root-mean-squared deviation (RMSd)

The RMSD was calculated for the protein backbone and the phospholipid substrate after aligning with the initial minimized structure. The RMSD profiles for the six replicates (REP1-6) are plotted in **Figure S8**. Overall, the backbone atoms remained stable throughout the 250 ns simulations, with RMSD values below 2.2 Å, within the crystal structure's resolution (2.54 Å). The first replicate was the exception. In the final 75 ns of the simulation, the latter had a nearly 1.0 Å increase of RMSD, while the other replicates promptly reached the equilibrium after 50 ns. The absence of significant shifts in the overall RMSD values helps to confirm the stability of the svPLA$_2$ structure.

On the other hand, the substrate RMSD (**Figure S8**) displayed a substantial increase of ca. 2.5 Å along the trajectory in REP1, 3, 4, and 6. Conversely, REP5 displayed a comparatively steadier RMSD profile, remaining at a constant value of 4 Å. This may also suggest that throughout the 250 ns of the simulation, REP5 has a stronger and more stabilized svPLA$_2$:POPC bond inside the active site cleft. These differences in the RMSD spectra might be due to the interaction between the phospholipid tails and the membrane and between the phospholipid headgroup (phosphate and choline groups) and the surrounding environment, namely the solvent. REP5 retains a more stable interaction with the membrane, whereas REP 1, 3, 4, and 6 RMSDs exhibit significant variations.

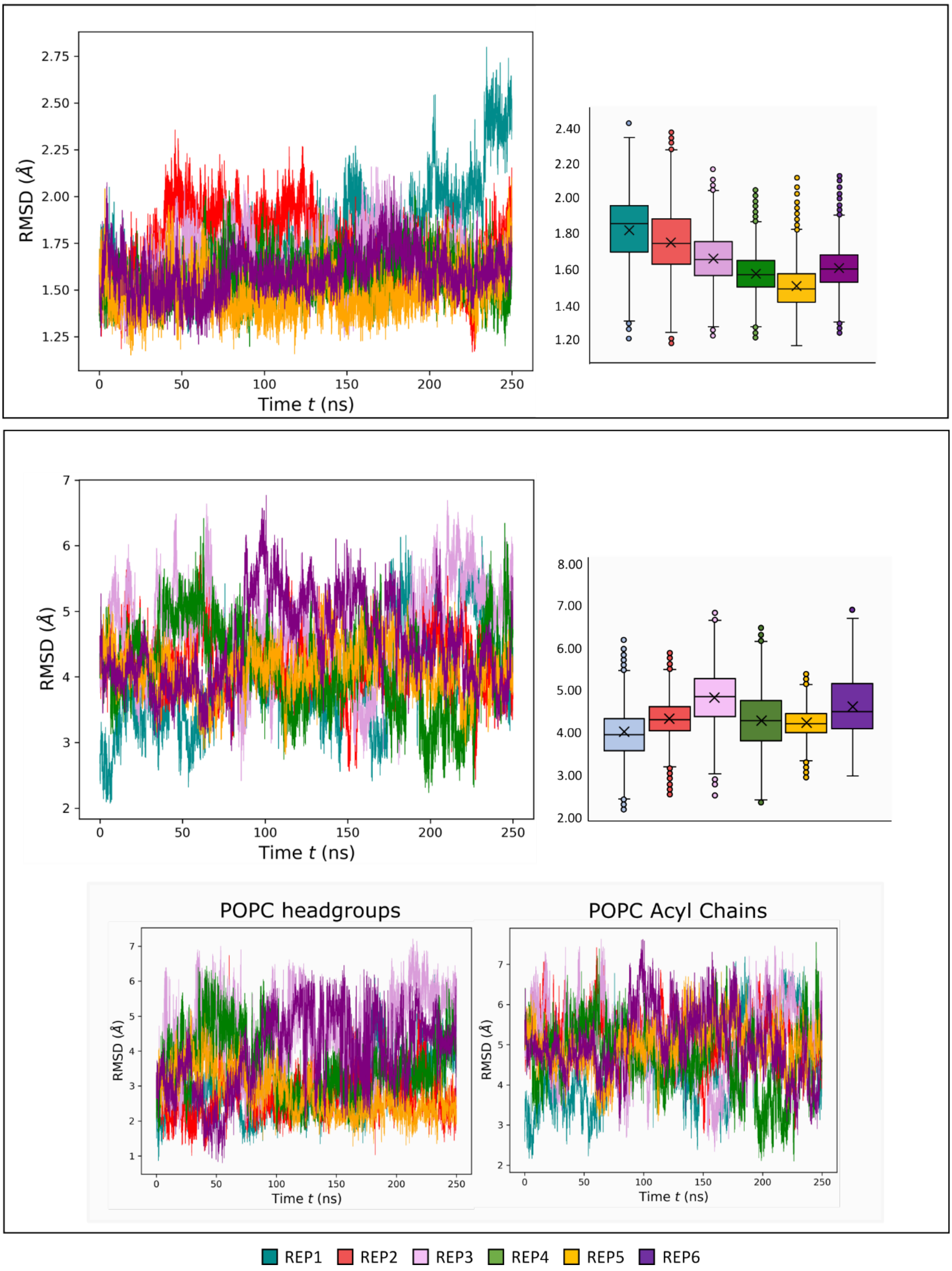

Figure S8: Graphical representation showing the RMSD profiles and mean values of the protein (top) and the substrate (bottom) for REP1 (teal color), REP2 (red color), REP3 (pink color), REP4 (green color), REP5 (yellow color) and REP6 (purple color) during the course of the 250-ns MD simulation. The RMSD values of the substrate's headgroups and acyl chains is also represented. Higher RMSD values imply low stability of the system.

Catalytic Distances

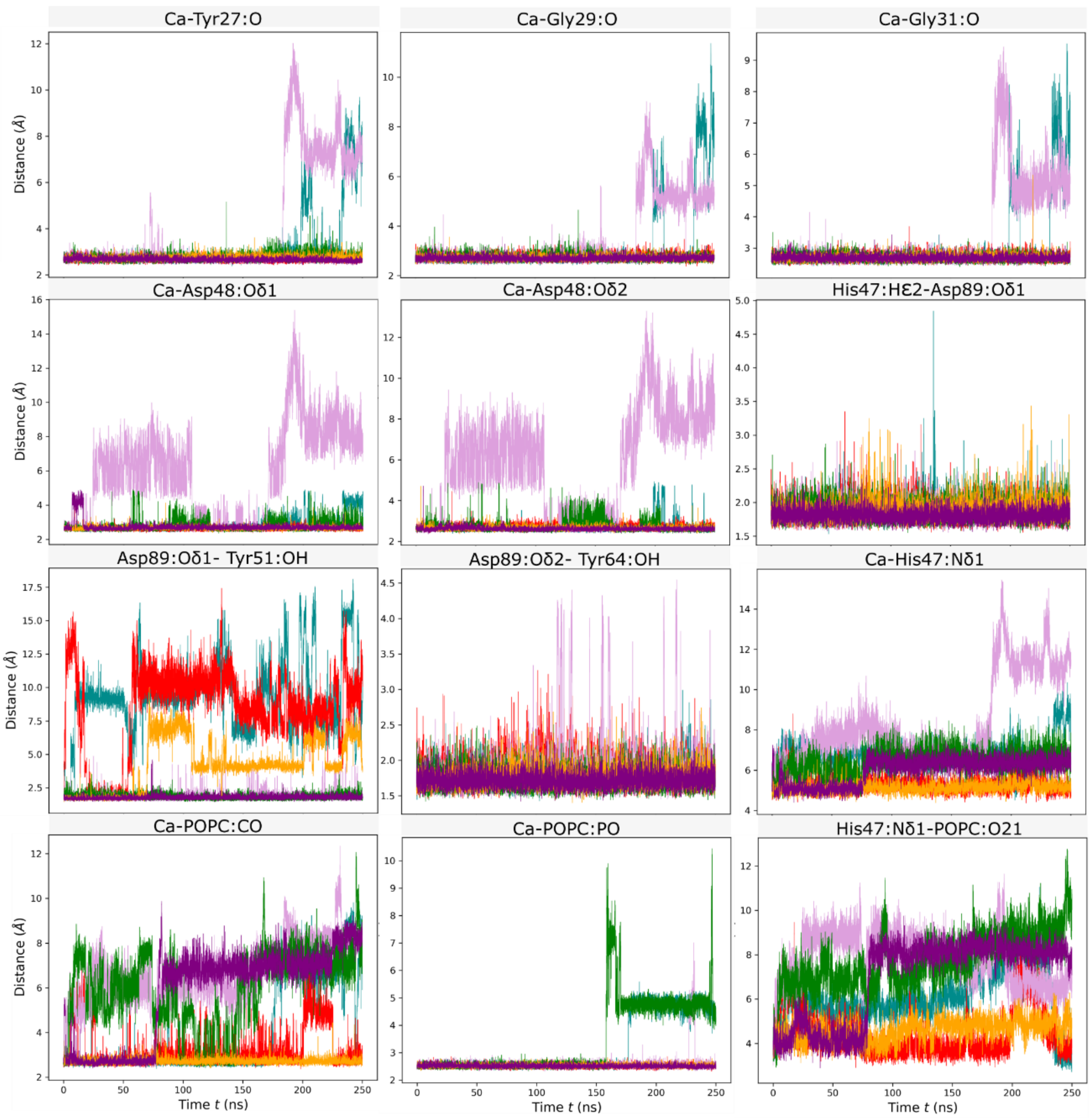

Figure S9: Representation of critical amino acid residues distances of along the simulation time. Each color represents a different replica: REP1 (dark cyan), REP2 (red), REP3 (pink), REP4 (green), REP5 (yellow) and REP6 (purple).

According to the initial literature-described structure, Tyr51 engages in several interactions. Notably, it forms a π-stacking interaction with His47 and takes part in some hydrogen bonding interactions, including one between its nitrogen atom and the oxygen of His47 (N_{Tyr51}-O_{His47}), and another between its oxygen atom and the nitrogen of Lys55 (O_{Tyr51}-N_{Lys55}), with bond distances of 2.8 and 2.9 Å (Figure S9), respectively.

The mobility and dissociation of Tyr51 from its interaction with Asp89 observed both in the distance distribution (**Figure S9**) and in the RMSF (**Figure S11**) results of REP5, can be attributed to some dynamic changes in the local structural environment throughout the production phase. Thus, the secondary structure dynamics were monitored using the VMD timeline tool. This analysis was particularly focused on the region of the protein containing the third α-helix (α3) where Tyr51 is originally located.

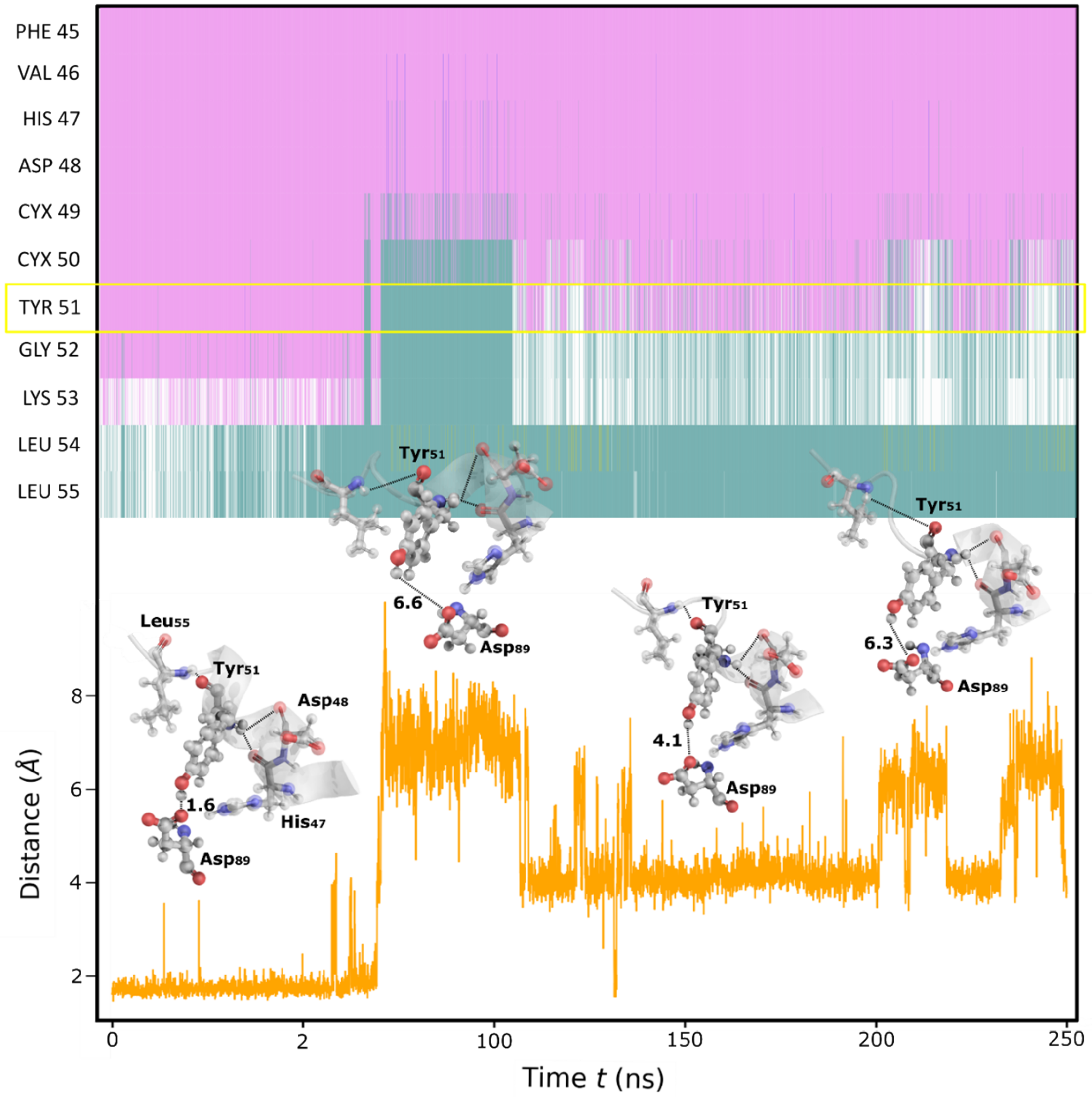

Figure S10: Analysis of the conformational dynamics of Tyr51 on REP5. **(Top)** Secondary structure prediction for residues 45 to 55 along the simulation trajectory. The color code is as follows: Pink - α-Helix, Green - Turn, White - Random Coil. Tyr 51 is highlighted with a yellow box to facilitate observation of its secondary structure evolution. **(Bottom)** Analysis of the distance between the hydrogen atom of Tyr 51 (HTyr51) and the oxygen atom of Asp 89 (OAsp89). The complementary figures provide snapshots illustrating the mobility of Tyr 51 and the presence or absence of the α-helix structure throughout the simulation.

Considering the results obtained in **Figure S10**, the secondary structure of the third α-helix region, underwent a transition into a more flexible state, a loop (represented by the green color) or a random coil (represented by the white color). This transition places Tyr51 between a loop and the α-helix structure (represented by the pink color), resulting in the exposure of Tyr51 to the surrounding environment. The loss of its native secondary structure, i.e., its helical integrity, facilitates the entrance and clustering of water molecules around this region, explaining the disruption of the OH_{Tyr51}-$O\delta1_{Asp89}$ interaction.

Root-mean-squared fluctuations (RMSF)

The root-mean-squared fluctuations (RMSFs) were also computed for each residue (**Figure S11**). It is clear from all the replicates that certain regions of the enzyme, including Phe18, Trp30, Arg42, Cys50-Arg64 (α3), Ser67-Ile75 (β-wing), and the C-terminus, exhibit higher structural fluctuations compared to other regions.

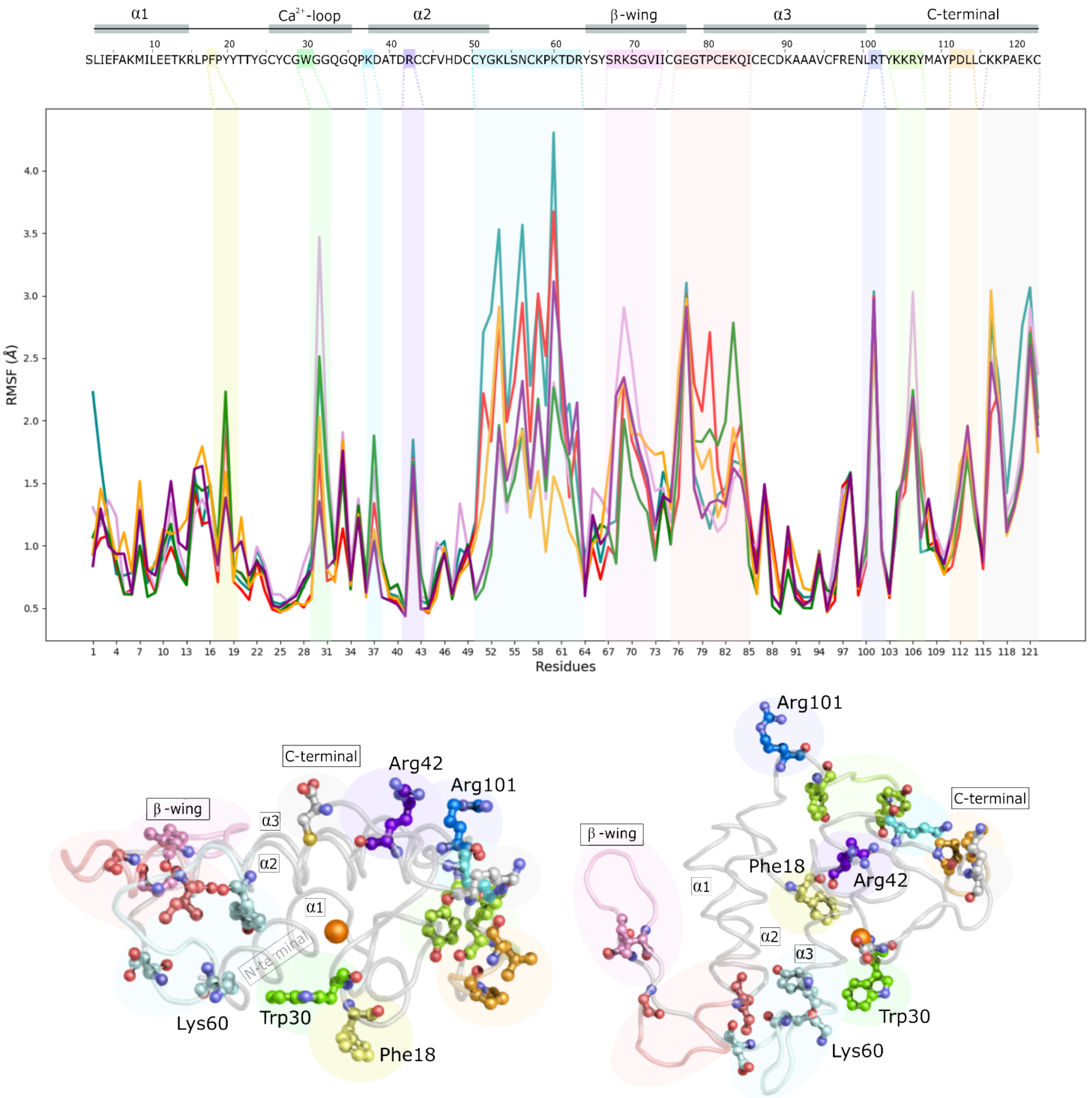

Figure S11: Graphical representation of the RMSF profiles for each replica: REP1 (teal color), REP2 (red color), REP3 (pink color), REP4 (green color), REP5 (yellow color) and REP6 (purple color) during the 250-ns MD simulation. The regions/residues that underwent more fluctuations are respectively colored in both the profiles (top) and in the protein ribbon representation (bottom).

These are functionally relevant regions, and it has been shown that the C-terminal region actively takes part in interactions with the bilayer membrane [46, 47], and thus, its flexibility is not unexpected. Thus, it is important to acknowledge that these fluctuations may, in part, be attributed to the presence of the substrate and interactions with the membrane. In line with the previous analysis, REP1 and REP2 exhibited much higher fluctuation peaks than the other four replicates, with RMSF values reaching 4.3 Å and 3.6 Å at position 60 (Lys60), respectively.

Radial distribution function (RDF)

Radial distribution functions (RDFs) were computed for the Ca^{2+}, His48, Asp89, and substrate POPC residues, along with the determination of the hydration number surrounding the specified atoms, as illustrated in **Figure S12**.

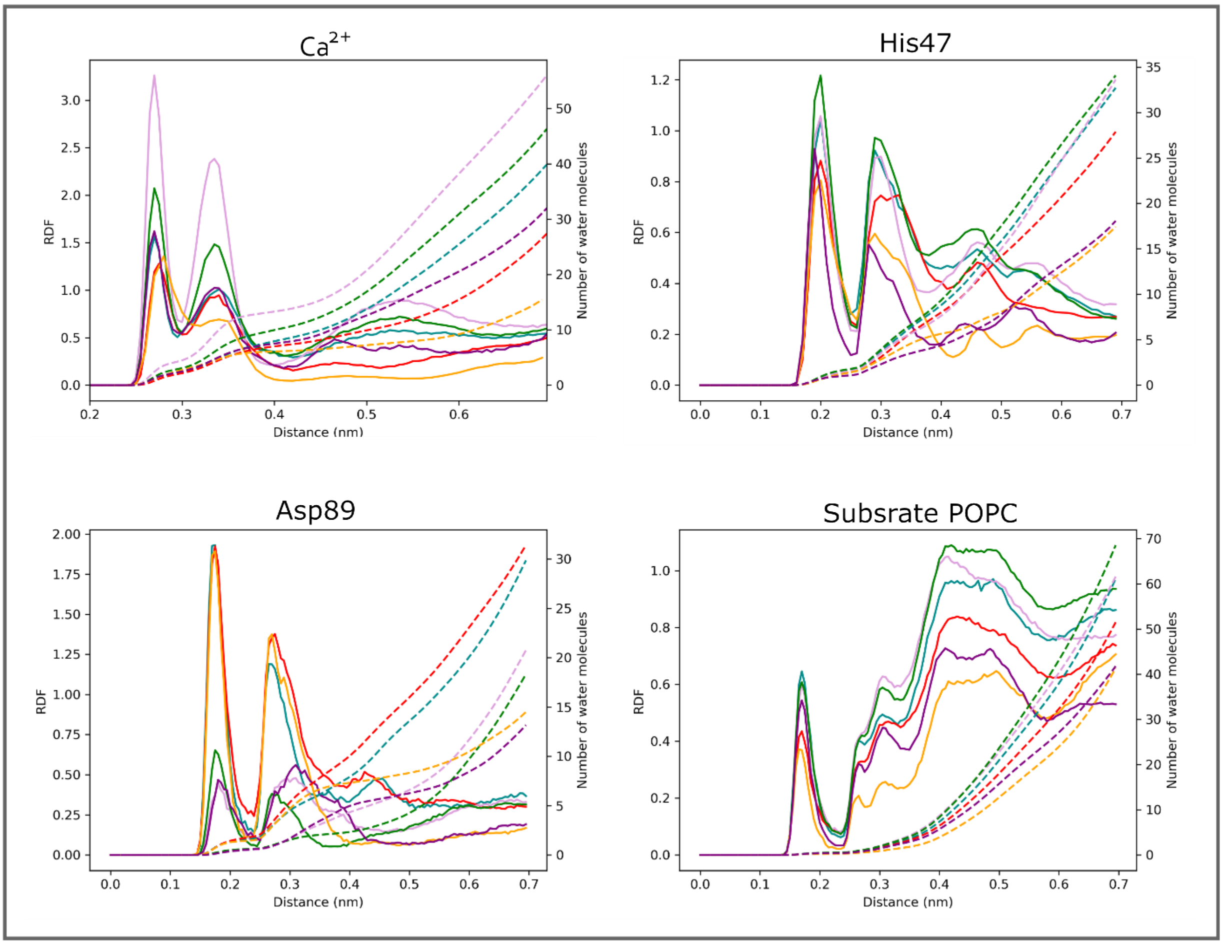

Figure S12: Radial Distribution Functions (RDF) depicting the spatial distribution of water molecules around Ca^{2+}, His47, Asp89 and the substrate POPC, along with the cumulative values (dashed lines) representing hydration area coverage. Each color represents a different replica: REP1 (dark cyan), REP2 (red), REP3 (pink), REP4 (green), REP5 (yellow) and REP6 (purple).

The RDF plots, regarding Ca^{2+}-Wat interactions, show that the g(r) values corresponding to the first peak range from 1.2 (REP2) to the very high value of 3.2 (REP3) and depict the chance of finding two and four water molecules at a separation distance of 2.7 Å, respectively. The hydration numbers were determined by the average number of water molecules within the distance of the first minimum shown in the RDF plots for the established contacts. Additionally, a notable second peak is observed around 3.3 Å, which is especially pronounced in REP3 (pink line). This peak corresponds to a hydration number of 7 and could potentially explain the observed instability in the Ca^{2+}-binding loop region, as illustrated in the analysis of the catalytic distances (**Figure S9**).

In the case of His48, a peak was observed at 2.0 Å featuring g(r) values ranging from 0.8 (REP5) to 1.2 (REP4) showing the probability of detecting one or two water molecules at that distance.

The Asp89-Wat radial distribution exhibits a peak at 1.75 Å with a g(r) value of 1.93 (REP1), 1.92 (REP2) and 1.89 (REP5). This pattern aligns with the results presented in Figure S9 and Figure S11, reinforcing the hypothesis that the loss of secondary structure makes it easier for water molecules to enter and destabilize the Asp89-Tyr51 bond.

Finally, RDF calculations for substrate POPC-Wat interactions reveal a peak at 1.7 Å, with g(r) values ranging from 0.36 (REP5) to 0.60 (REP4). This indicates a low probability of finding a water molecule in proximity to the substrate.

Hydrogen bonds

The analysis of the hydrogen bond interactions between the enzyme and substrate during the trajectory, with a cutoff radius of 0.3 nm, has produced some insightful results (**Figure S13**).

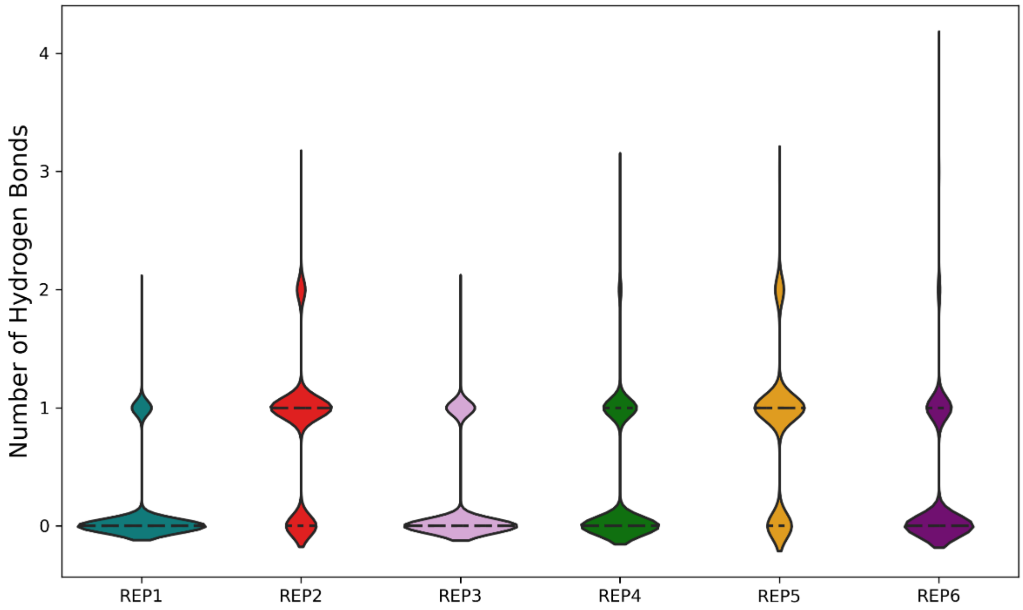

Figure S13: Violin plots summarizing the number of hydrogen bond interactions formed during MD simulation in REP1-6 between BaMt-I and the substrate POPC. Dashed lines represent the mean value of hydrogen bonds. Each color represents a different replica: REP1 (dark cyan), REP2 (red), REP3 (pink), REP4 (green), REP5 (yellow) and REP6 (purple).

The results revealed that one hydrogen bond forms between the protein and the substrate in more than 60% of frames across REP2 and REP5 trajectories. This well-known hydrogen bond, which involves the connection between the substrate POPC phosphate oxygen atom and the Gly29 amine group (H_{Gly29}-$O14_{popc}$), is documented in the literature [48]. In addition to this predominant interaction, the analysis revealed that the number of hydrogen bonds also fluctuated between 2 and 3, occurring over a small fraction of the trajectory. These fluctuations correspond to interactions between the Gly29 amine group and the POPC carbonyl group (H_{Gly29}-$O31_{popc}$) and between the amine group of Lys60 and the substrate POPC phosphate oxygen atom (H_{Lys60}-$O14_{popc}$). Notably, the importance of these interactions in the hydrophobic channel is acknowledged in the literature [48, 49].

Altogether, these interactions might be crucial for the stabilization of the POPC substrate in the active center. This assumption can be supported by the results displayed in **Figure S9**, where the Ca^{2+}-$O31_{popc}$ distance has only remained constant in REP2 and REP5.

In contrast, the remaining four replicas demonstrate a lack of hydrogen bonds along nearly the whole trajectory - more than 89%. This absence of inter-molecular hydrogen bonds might have some repercussions on the stability of the svPLA$_2$:POPC complex, highlighting, once again, the higher stability and suitability of REP5 for the study of the reaction mechanism.

The overall behavior observed across the different replicas was quite similar as shown in **Figures S8** and **S9**, indicating consistency in the simulation results. However, some exceptions were noted regarding specific distances: Tyr51 OH – Asp89 Oδ1, His47 Nδ– Cpopc and Ca2+– COpopc.

Given that REP5 demonstrated greater stability and met all relevant distance criteria for subsequent mechanistic studies, it was selected for further studies.

Membrane Analysis

The impact of svPLA2 penetration on the integrity of the membrane model was analyzed by determining variations in the area per lipid (ΔAPL) and lipid bilayer thickness (ΔT) using the FATSLiM software. Results obtained for both properties were taken from the last 250 ns and are depicted in **Figure S14**.

Before the insertion of the protein, the area per lipid (APL) was around 63.05 ± 0.1 Å^2. However, as the enzyme was progressively inserted into the membrane, APL increased across all replicas (**Figure S14**) when compared to the initial production step during membrane equilibration. The APL of the POPC:POPS membrane was found to be around 64.4 ± 0.1 Å^2, which is consistent with previously reported values ranging from 65 to 70 Å^2 [50-55].

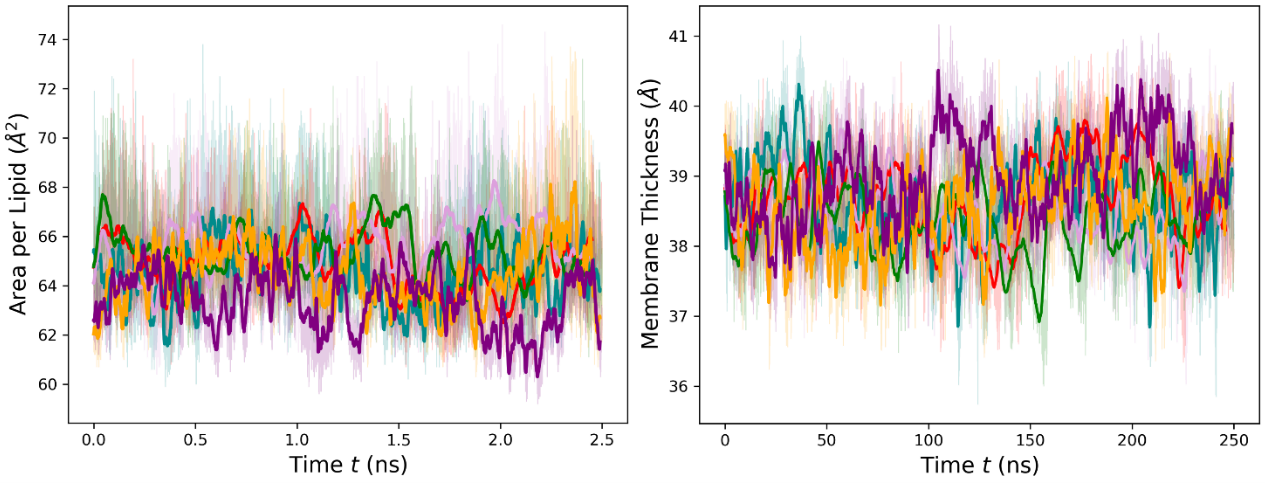

Figure S14 – Membrane properties over the simulation time: Area per lipid (left) and membrane thickness (right); B) Deuterium order parameter profile of *sn*1 and *sn*2 acyl chains of the POPS (left) and POPC (right) lipids in each replica. S_{CD} values around 1.0 indicate a completely ordered state of lipid moieties acyl chains, whereas values near 0 indicate a completely disordered state.

On the other hand, **Figure S14B** illustrates changes in membrane thickness during the MD simulations, showing a decrease in thickness in the presence of svPLA2 when compared to the membrane equilibration stage (39.4 ± 0.1 Å2). This is likely due to the interaction of the svPLA2:POPC complex with the membrane, which induces membrane deformation by insertion, increased APL and subsequently thinning.

Therefore, the increase in APL upon svPLA2 insertion onto the membrane surface allows for the relaxation of lipid acyl chains, displacement of phosphate headgroups and thinning of the membrane. These results are well within the range of values reported in both computational and experimental studies [50-55], demonstrating a remarkable correlation. This indicates that the physical characteristics of the POPC:POPS lipid bilayer were described with a high level of accuracy.

To unravel the potential influence of membrane interactions on the structural dynamics of BaMt-I, the distance between the c.o.m of the Lys7, Arg63 His47, and Tyr51 atoms and the c.o.m of the bilayer normal along the z-axis was also

measured (**Figure S15**). This analysis sought to elucidate whether interactions with the membrane could contribute to the observed destabilization of the enzyme's secondary structure around Tyr51. As such, the other residues were used for control. The side chain of the Tyr 51 is positioned at an average distance of 9 Å from the bilayer center, which corresponds to a location close to the headgroup/tail interface. The side chain of the catalytic His47 residue is located approximately 12 Å from the center, corresponding to an area above the bilayer surface. However, it is buried on the floor of the active site cleft, away from the protein's surface.

Consequently, there was no significant deviation from its side chain's initial position (**Figure S15**). This aligns with the PLA2's X-ray structures, which show a deep active site slot of around 15 Å that can accommodate around half of a phospholipid molecule's length [56]. Through a comparative analysis of these features and further analysis of Tyr51's surroundings, it was found that Tyr51 establishes some contacts with the oxygen atoms (O13 and O14) in the phosphate groups of POPC125 and POPC126. This further explains the loss of secondary structure in this area (**Figure S10**), as well as the loss of the interaction between the $Tyr51_{OH}$ and the $Asp89_{O\delta1}$ atoms, aligning with both the distance (**Figure S9**) and the RMSF (**Figure S11**) analysis.

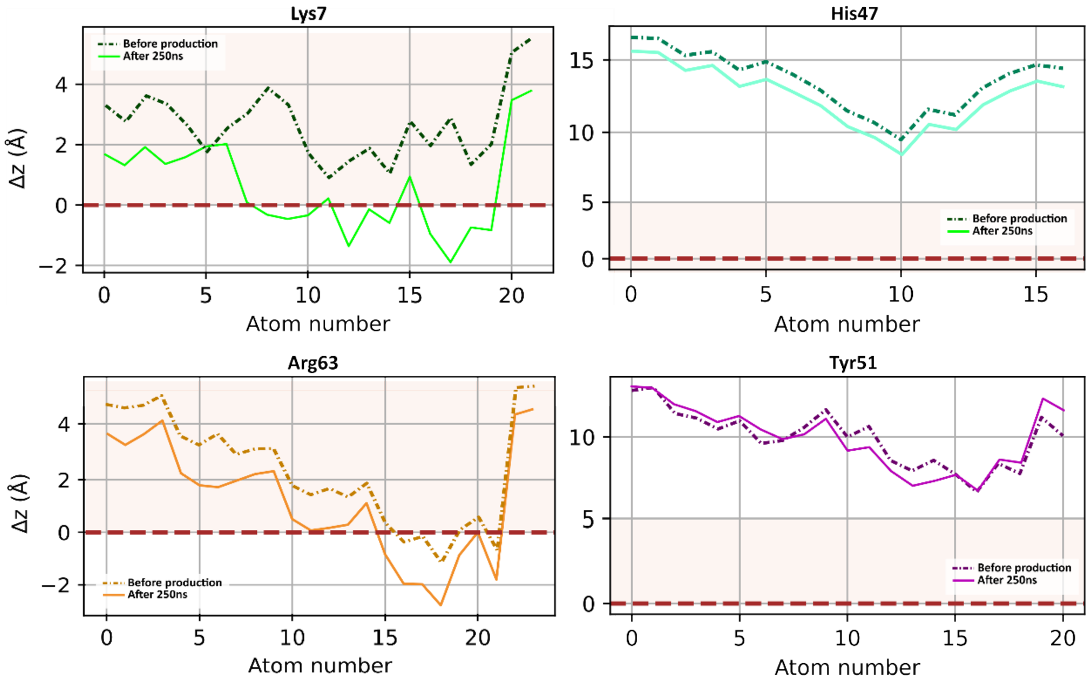

Figure S15: Average z-distance between Lys7, Arg63, His47 and Tyr51 each atom's center of mass (COM) and the lipid bilayer's normal. The membrane plane was defined by averaging the z-coordinates of the lipid's phosphates within approximately 15 Å of each residue. Solid lines show the z-distance at the end of the production time (250ns), whereas dashed lines show the initial z-distance of each atom COM. to the membrane before this production step.

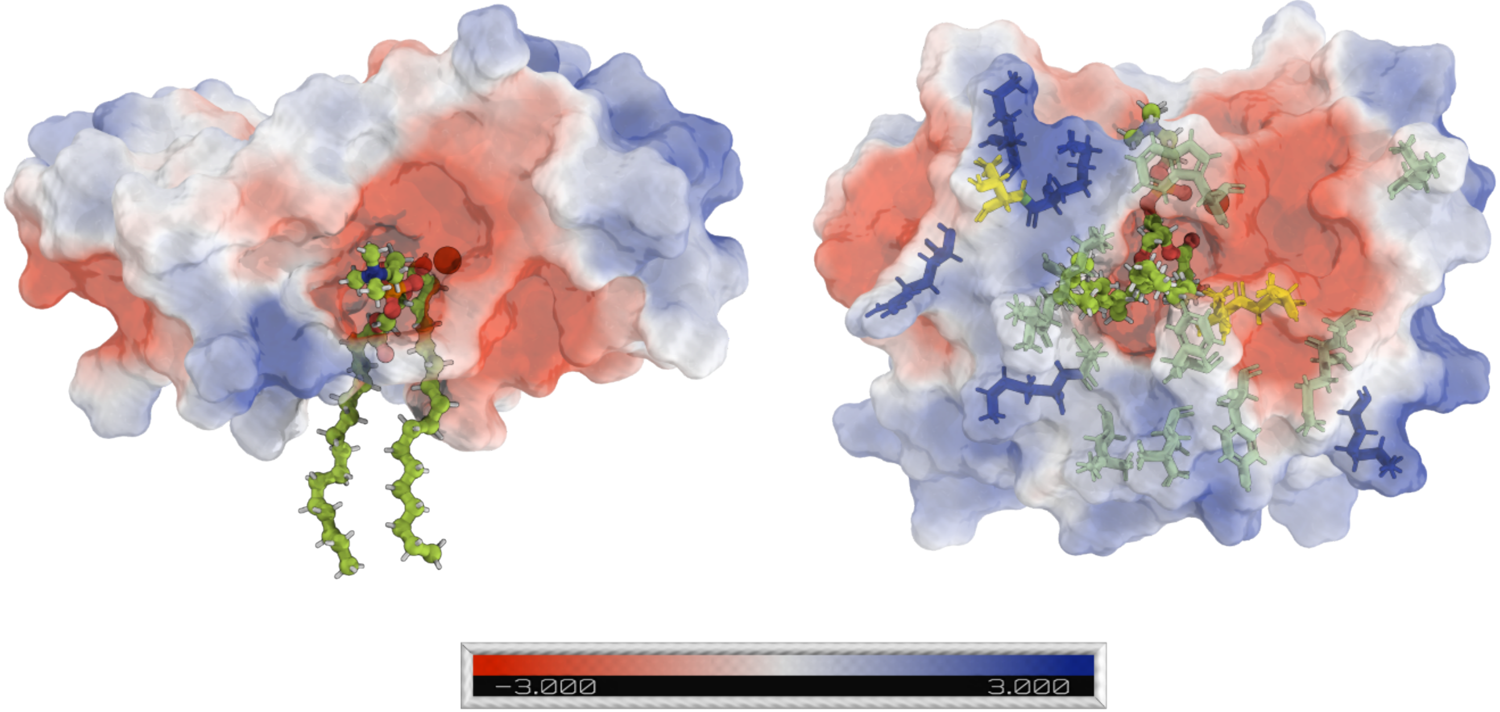

Figure S16: Surface charge distribution of the binding surface of BaMt-I. The electrostatic potential surface was calculated using PyMOL 2.0 APBS plugin. Negative, positive, non-polar, and polar residues are colored in red, blue, light green and yellow. In the right panel, positive residues are shown in blue, hydrophobic in green, and neutral hydrophilic in yellow. The positive patch at the left is composed of the Lys58 and Lys60. The ∊-ammonium group of the former establishes hydrogen bonds with the sn-3 phosphate of the productively-bound POPC (shown in green sticks) in the hydrophobic channel. The red and blue colors indicate regions of negative and positive electrostatic potential on the surface, respectively, with potential values varying between ±3 kT/e. The color white corresponds to neutral potential.

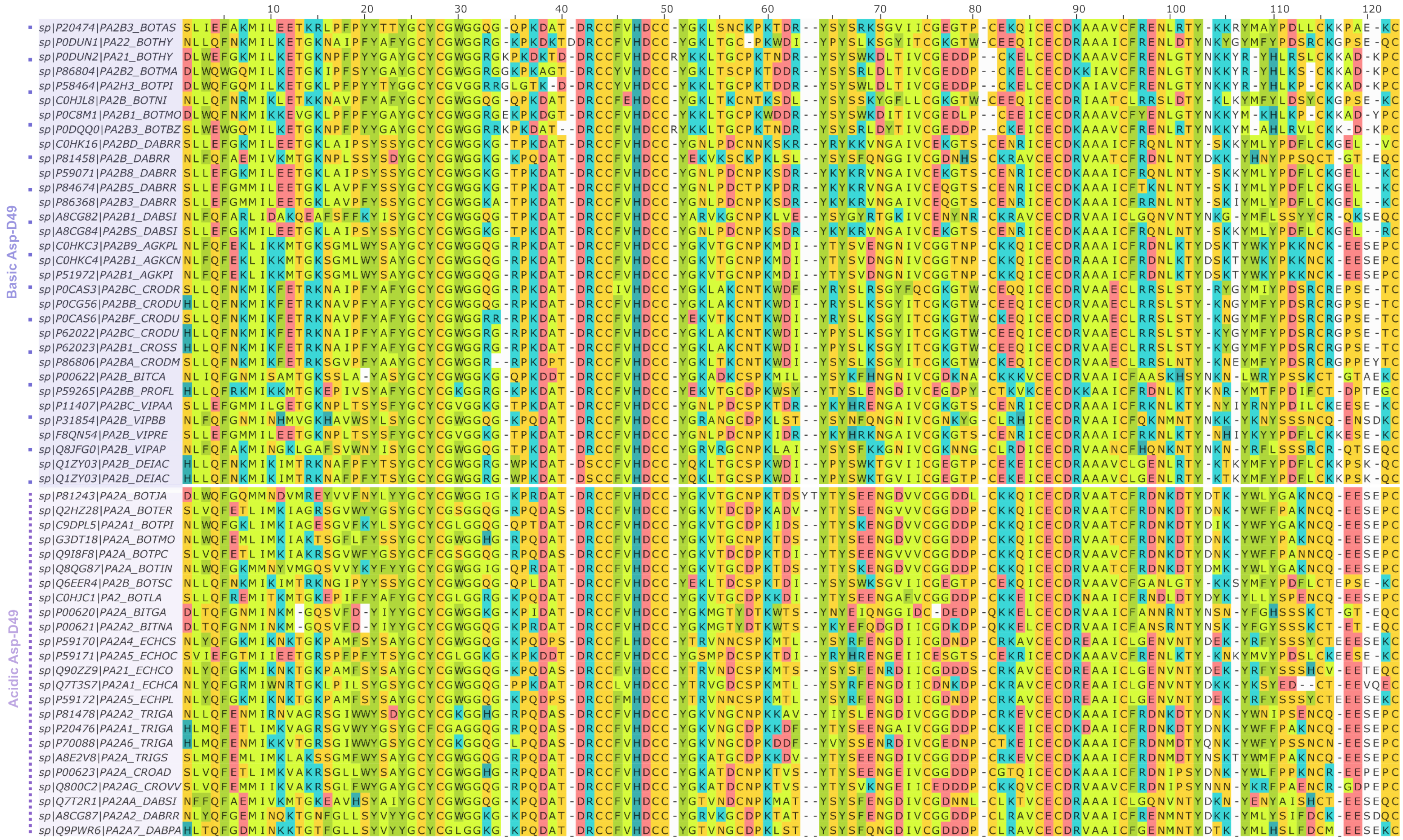
Basic Asp-D49
Acidic Asp-D49

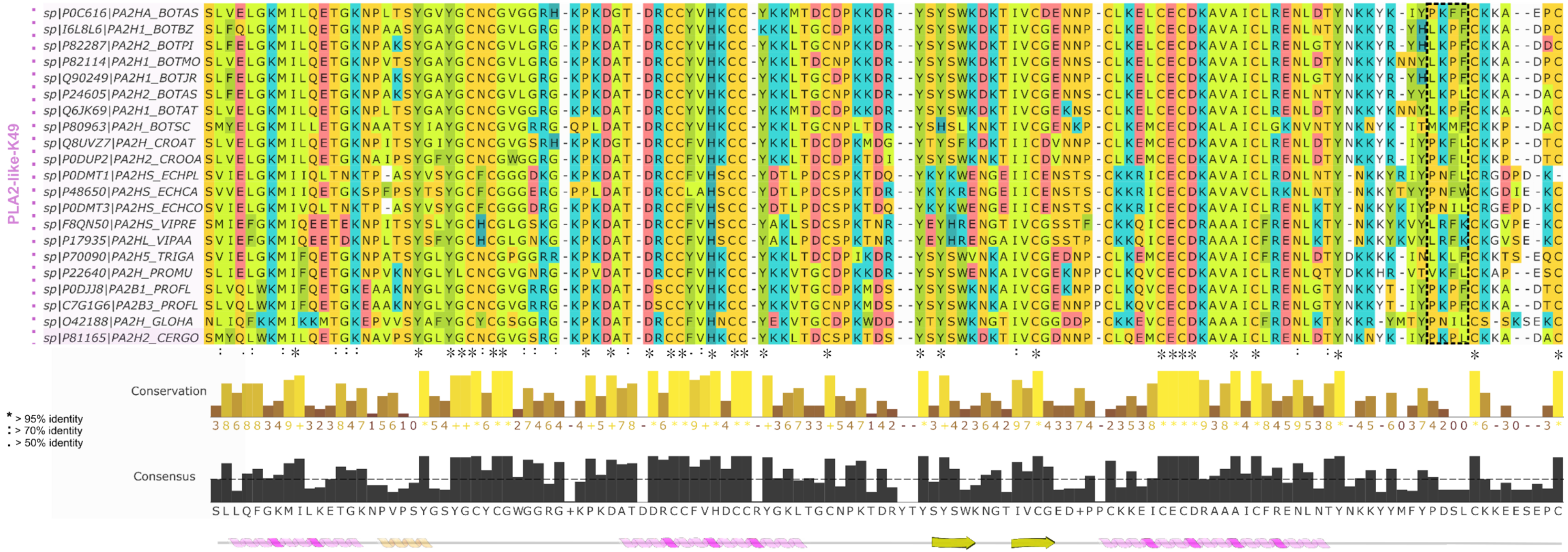

Figure S17: A multiple sequence alignment of both basic and acidic svPLA2 enzymes, as well as their homologue counterparts (PLA2-like-K49), was conducted for 70 sequences. Residues were colored according to their physicochemical properties. Hydrophobic residues were colored green, positively charged residues blue, and negatively charged residues red, aromatics in dark green and hydrophilic in orange. Additionally, the consensus sequence (most frequent residues) and the residues occupancy (presence or absence of gaps) are shown. A black dashed box shows the residues that belong to the hydrophobic knuckle present in the PLA_2-like-K49 enzymes.

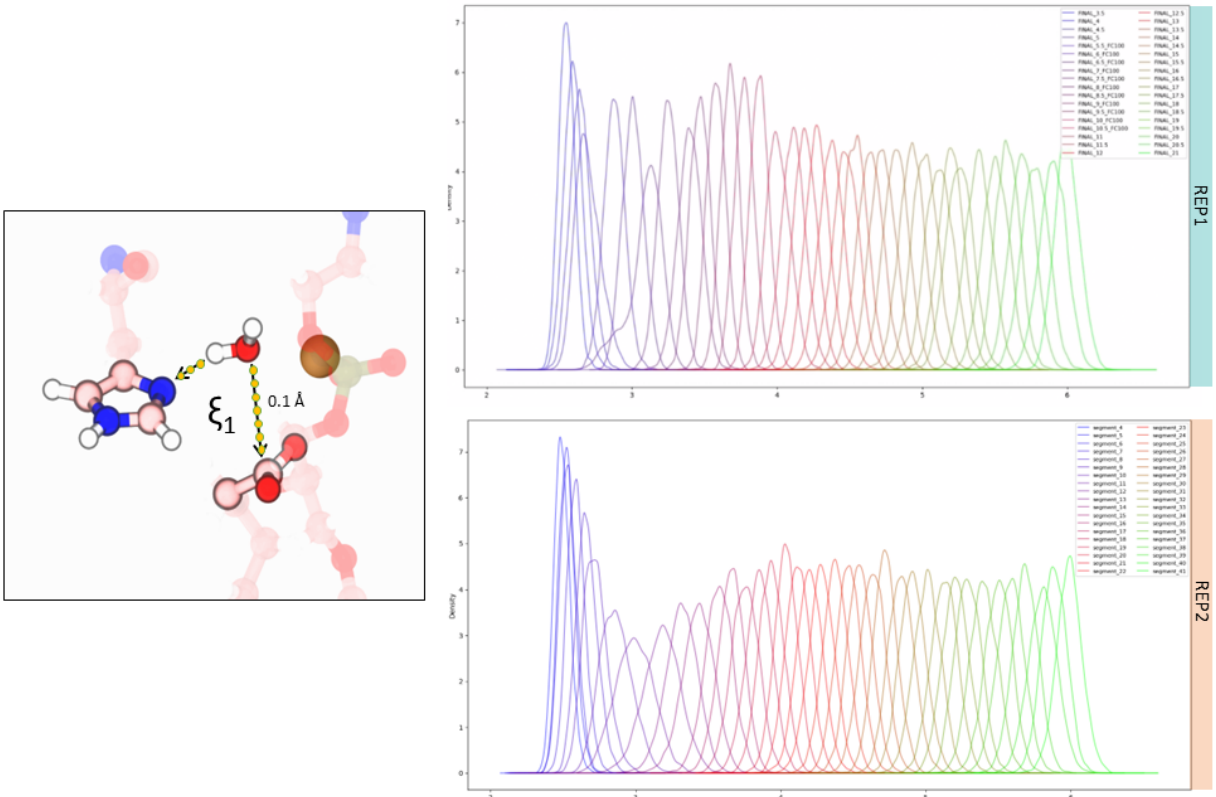

Figure S18: Histograms depicting the distributions of configurations sampled within the US windows for the CV1 of the single-water mechanism in REP1 and REP2.

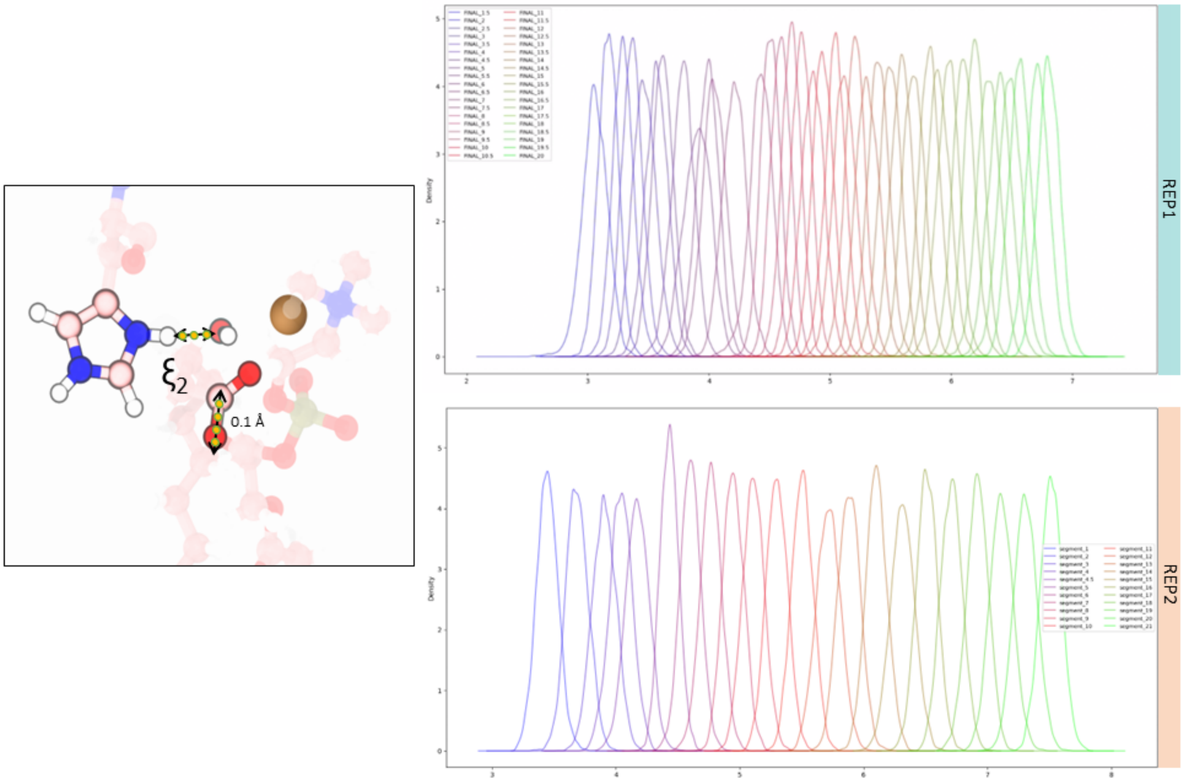

Figure S19: Histograms depicting the distributions of configurations sampled within the US windows for the CV2 of the single-water mechanism in REP1 and REP2.

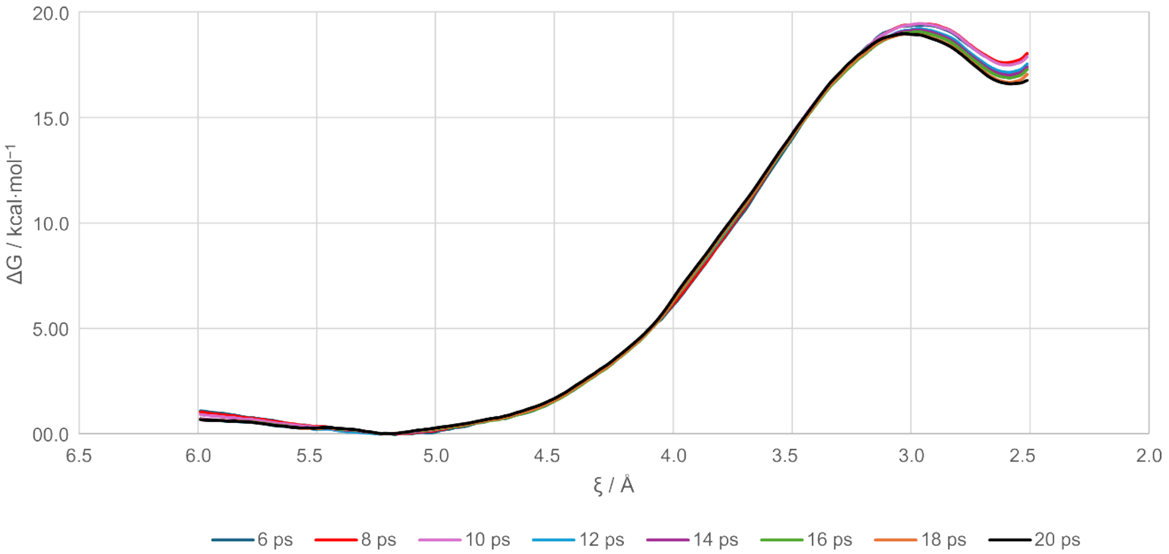

Figure S20: Cumulative free energy profiles across different time blocks of 2 ps along ξ1 reaction coordinate within the single-water pathway over time in both replicas.

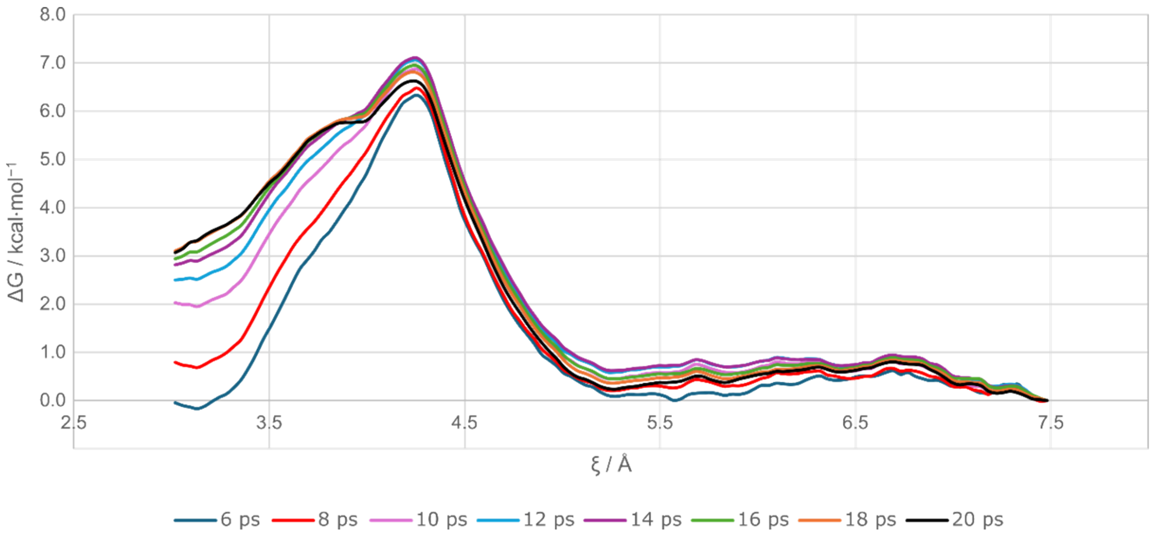

Figure S21: Cumulative free energy profiles across different time blocks of 2 ps along ξ2 reaction coordinate within the single-water pathway over time in both replicas.

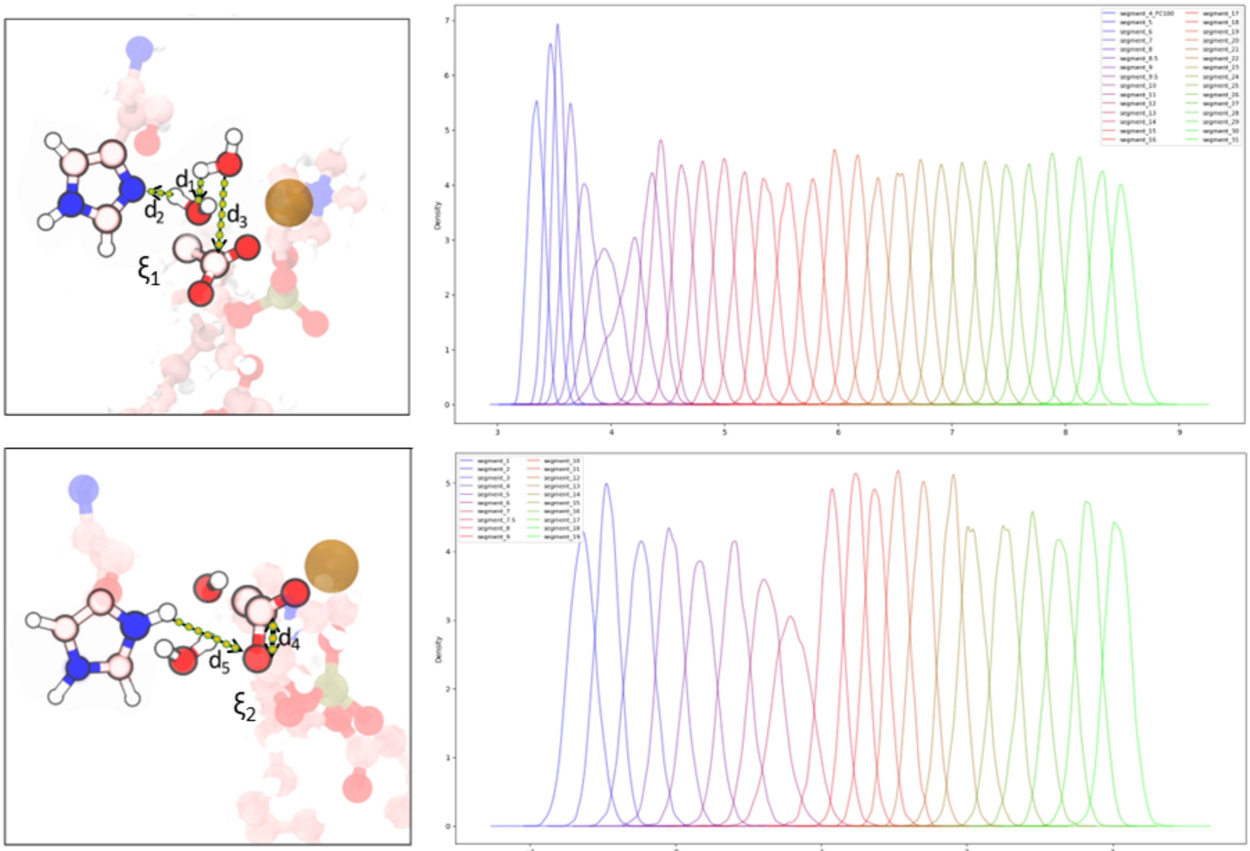

Figure S22: Histograms depicting the distributions of configurations sampled within the US windows for both CV1 and CV2 of the assisted-water mechanism.

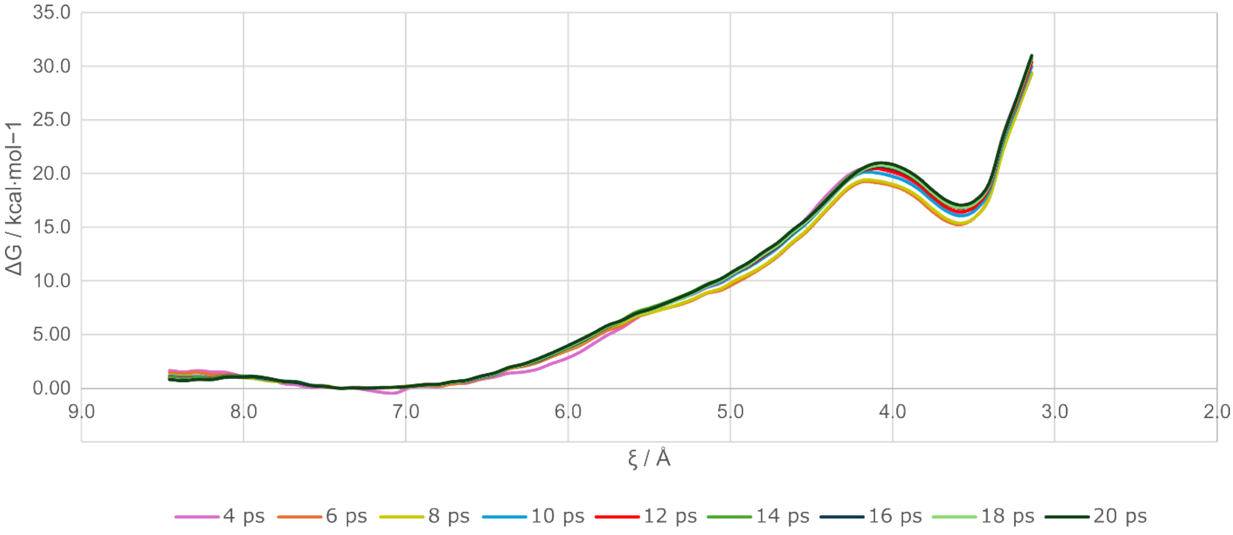

Figure S23: Cumulative free energy profiles across different time blocks of 2 ps along the ξ1 reaction coordinate within the assisted-water pathway over time.

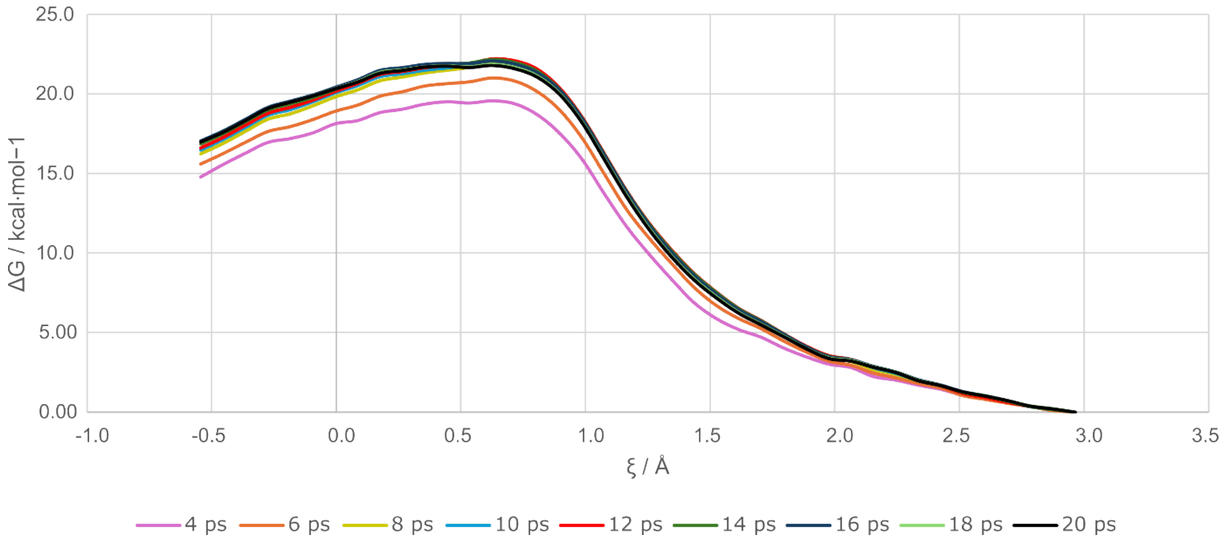

Figure S24: Cumulative free energy profiles across different time blocks of 2 ps along the ξ2 reaction coordinate within the assisted-water pathway over time.

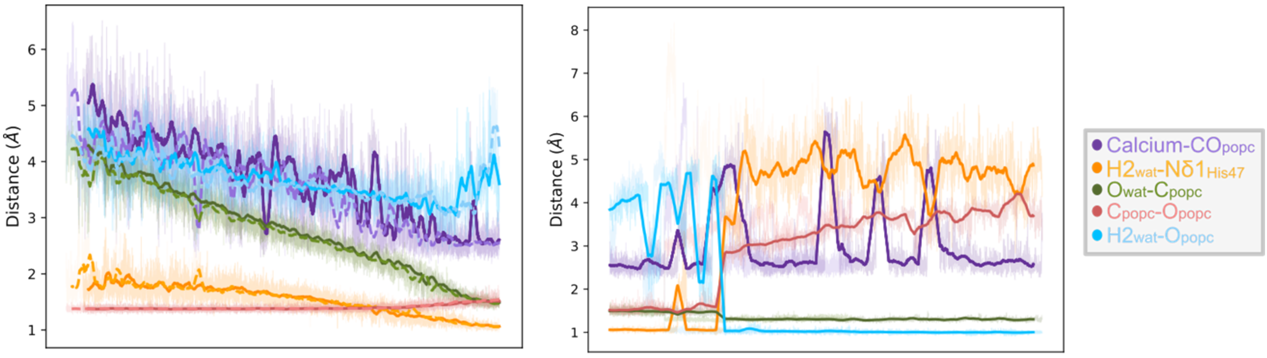

	REACT		TS1		INT		TS2		PROD	
**Distances**	**Mean**	**Stdev**	**Mean**	**Stdev**	**Mean**	**Stdev**	**Mean**	**Stdev**	**Mean**	**Stdev**
Calcium-COpopc	4.7650	0.6574	2.6492	0.2587	2.5612	0.1412	3.4515	0.8954	2.5837	0.1801
H2wat-Nδ1His47	1.9116	0.3223	1.1768	0.0862	1.0680	0.0398	1.0633	0.0515	4.8385	0.4884
OWat-Cpopc	4.0305	0.3452	1.7362	0.1464	1.4995	0.0506	1.4779	0.0523	1.3160	0.0399
H1Wat-OD1Asp	2.6562	0.8907	1.9820	0.2872	2.0504	0.3455	4.7889	2.4060	1.3398	0.2930
Cpopc-Opopc	1.3755	0.0340	1.4661	0.0584	1.5131	0.0589	1.5727	0.0805	3.7994	0.2629
H2wat-Opopc	4.2885	0.4131	3.1823	0.2906	3.8589	0.3981	4.6741	0.2482	1.0020	0.0323

Figure S25: Average atomic distances of key catalytic residues along the ξ1 reaction coordinate (**top, left**) and the ξ2 reaction coordinate (**top, right**) of the umbrella sampling simulations for the single-water mechanism; Table with the mean and standard deviation values for each stationary point (**bottom**).

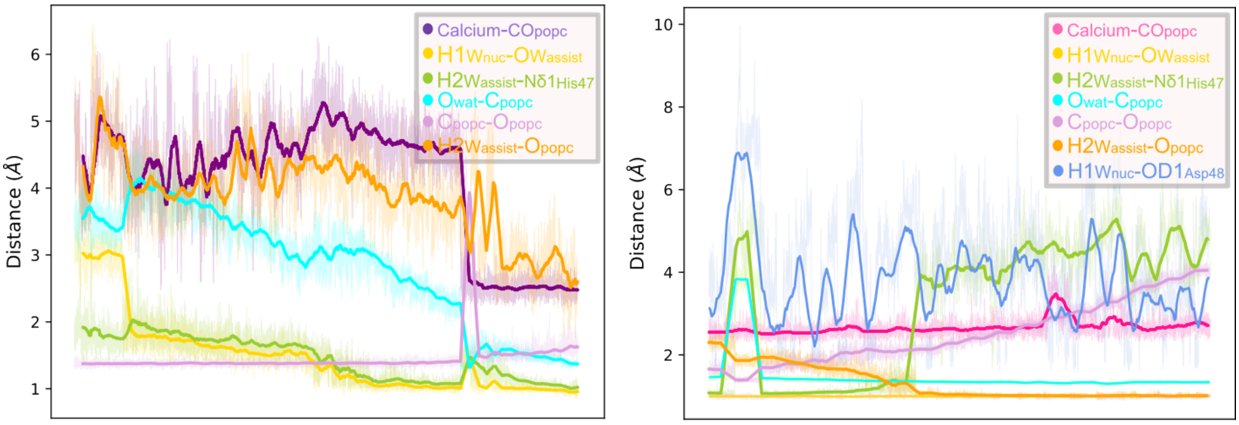

	REACT		TS1		INT		TS2		PROD	
**Distances**	**Mean**	**Stdev**	**Mean**	**Stdev**	**Mean**	**Stdev**	**Mean**	**Stdev**	**Mean**	**Stdev**
Ca-CO	4.3749	0.5499	2.5384	0.1404	2.5213	0.1176	2.6237	0.1679	2.6799	0.1762
HWnuc-OWassist	2.5874	0.5838	1.1209	0.1797	0.9972	0.0330	0.9958	0.0302	1.0036	0.0378
H2Wassist-Nδ1His47	1.8785	0.2722	1.3017	0.1534	1.0848	0.0483	1.2067	0.1560	4.5707	0.6439
OWnuc-Cpopc	3.7309	0.3966	1.5356	0.1362	1.4830	0.0591	1.3727	0.0372	1.3369	0.0327
H1W-OD1Asp	2.1417	0.4245	2.5333	0.9796	1.8424	0.1873	1.6925	0.1433	1.5482	0.1530
Cpopc-Opopc	1.3750	0.0346	1.5653	0.9701	1.5842	0.1048	2.0815	0.1461	3.8493	0.1799
H2Wassist-Opopc	4.2589	0.5879	3.3477	0.7148	2.5895	0.4505	1.4674	0.2073	1.0113	0.0334

Figure S26: Average atomic distances of key catalytic residues along the ξ1 reaction coordinate (**top, left**) and the ξ2 reaction coordinate (**top, right**) of the umbrella sampling simulations for the assisted-water mechanism; Table with the mean and standard deviation values for each stationary point (**bottom**).

References

1. A. L. Oliveira, M. F. Viegas, S. L. da Silva, A. M. Soares, M. J. Ramos and P. A. Fernandes, *Nature Reviews Chemistry*, 2022, **6**, 451-469.
2. T. Tasoulis and G. K. Isbister, A Review and Database of Snake Venom Proteomes.*Journal*, 2017, **9**.
3. A. Alape-Giron, L. Sanz, J. Escolano, M. Flores-Diaz, M. Madrigal, M. Sasa and J. J. Calvete, *J Proteome Res*, 2008, **7**, 3556-3571.
4. G. H. Salvador, J. I. Dos Santos, B. Lomonte and M. R. Fontes, *Biochimie*, 2017, **133**, 95-102.
5. J. Jasti, M. Paramasivam, A. Srinivasan and T. Singh, *Acta Crystallographica Section D: Biological Crystallography*, 2004, **60**, 66-72.
6. S. P. White, D. L. Scott, Z. Otwinowski, M. H. Gelb and P. B. Sigler, *Science*, 1990, **250**, 1560-1563.
7. D. L. Scott, S. P. White, J. L. Browning, J. J. Rosa, M. H. Gelb and P. B. Sigler, *Science*, 1991, **254**, 1007-1010.
8. D. L. Scott, Z. Otwinowski, M. H. Gelb and P. B. Sigler, *Science*, 1990, **250**, 1563-1566.
9. M. M. Thunnissen, E. Ab, K. H. Kalk, J. Drenth, B. W. Dijkstra, O. P. Kuipers, R. Dijkman, G. H. de Haas and H. M. Verheij, *Nature*, 1990, **347**, 689-691.
10. M. D. Hanwell, D. E. Curtis, D. C. Lonie, T. Vandermeersch, E. Zurek and G. R. Hutchison, *J Cheminform*, 2012, **4**, 17.
11. T. J. Dolinsky, J. E. Nielsen, J. A. McCammon and N. A. Baker, *Nucleic acids research*, 2004, **32**, W665-W667.
12. M. H. Olsson, C. R. Søndergaard, M. Rostkowski and J. H. Jensen, *Journal of chemical theory and computation*, 2011, **7**, 525-537.
13. A. V. Pinto, P. Ferreira, A. V. Cunha, R. W. A. Havenith, A. L. Magalhaes, M. J. Ramos and P. A. Fernandes, *Chem Sci*, 2024, **15**, 9793-9805.
14. S. Jo, T. Kim and W. Im, *PLoS One*, 2007, **2**, e880.
15. S. Jo, T. Kim, V. G. Iyer and W. Im, *Journal of Computational Chemistry*, 2008, **29**, 1859-1865.
16. J. P. Jambeck and A. P. Lyubartsev, *Journal of chemical theory and computation*, 2013, **9**, 774-784.
17. J. P. Jambeck and A. P. Lyubartsev, *J Phys Chem B*, 2012, **116**, 3164-3179.
18. H. Antila, P. Buslaev, F. Favela-Rosales, T. M. Ferreira, I. Gushchin, M. Javanainen, B. Kav, J. J. Madsen, J. Melcr, M. S. Miettinen, J. Maatta, R. Nencini, O. H. S. Ollila and T. J. Piggot, *J Phys Chem B*, 2019, **123**, 9066-9079.
19. W. L. Jorgensen, J. Chandrasekhar, J. D. Madura, R. W. Impey and M. L. Klein, *The Journal of chemical physics*, 1983, **79**, 926-935.
20. I. S. Joung and T. E. Cheatham III, *The journal of physical chemistry B*, 2008, **112**, 9020-9041.
21. P. Li, L. F. Song and K. M. Merz Jr, *The Journal of Physical Chemistry B*, 2015, **119**, 883-895.
22. M. J. Abraham, T. Murtola, R. Schulz, S. Páll, J. C. Smith, B. Hess and E. Lindahl, *SoftwareX*, 2015, **1**, 19-25.
23. W. Van Gunsteren and H. J. Berendsen, *Molecular Physics*, 1977, **34**, 1311-1327.
24. B. Hess, H. Bekker, H. J. Berendsen and J. G. Fraaije, *Journal of computational chemistry*, 1997, **18**, 1463-1472.
25. H. G. Petersen, *The Journal of chemical physics*, 1995, **103**, 3668-3679.
26. S. Nosé, *Molecular Physics*, 2006, **52**, 255-268.
27. M. Parrinello and A. Rahman, *Journal of Applied physics*, 1981, **52**, 7182-7190.
28. D. Case, I. Ben-Shalom, S. Brozell, D. Cerutti, T. Cheatham III, V. Cruzeiro, T. Darden, R. Duke, D. Ghoreishi and M. Gilson, *Ta [Google Scholar]*, 2018.
29. J. VandeVondele, M. Krack, F. Mohamed, M. Parrinello, T. Chassaing and J. Hutter, *Computer Physics Communications*, 2005, **167**, 103-128.
30. J. P. Perdew, K. Burke and M. Ernzerhof, *Physical review letters*, 1996, **77**, 3865.
31. B. G. Lippert, J. H. PARRINELLO and MICHELE, *Molecular Physics*, 1997, **92**, 477-488.
32. M. Krack, *Theoretical Chemistry Accounts*, 2005, **114**, 145-152.
33. F. Maseras and K. Morokuma, *Journal of Computational Chemistry*, 2004, **16**, 1170-1179.
34. T. Laino, F. Mohamed, A. Laio and M. Parrinello, *J Chem Theory Comput*, 2005, **1**, 1176-1184.
35. U. Essmann, L. Perera, M. L. Berkowitz, T. Darden, H. Lee and L. G. Pedersen, *The Journal of chemical physics*, 1995, **103**, 8577-8593.
36. G. Bussi, D. Donadio and M. Parrinello, *The Journal of chemical physics*, 2007, **126**.
37. G. M. Torrie and J. P. Valleau, *Journal of computational physics*, 1977, **23**, 187-199.
38. M. Andrec, The weighted histogram analysis method (WHAM).*Journal*, 2010.
39. W. Humphrey, A. Dalke and K. Schulten, *J Mol Graph*, 1996, **14**, 33-38, 27-38.
40. J. D. Hunter, *Computing in Science & Engineering*, 2007, **9**, 90-95.
41. C. R. Harris, K. J. Millman, S. J. van der Walt, R. Gommers, P. Virtanen, D. Cournapeau, E. Wieser, J. Taylor, S. Berg, N. J. Smith, R. Kern, M. Picus, S. Hoyer, M. H. van Kerkwijk, M. Brett, A. Haldane, J. F. Del Rio, M. Wiebe, P. Peterson, P. Gerard-Marchant, K. Sheppard, T. Reddy, W. Weckesser, H. Abbasi, C. Gohlke and T. E. Oliphant, *Nature*, 2020, **585**, 357-362.
42. W. McKinney, 2010.
43. R. T. McGibbon, K. A. Beauchamp, M. P. Harrigan, C. Klein, J. M. Swails, C. X. Hernandez, C. R. Schwantes, L. P. Wang, T. J. Lane and V. S. Pande, *Biophys J*, 2015, **109**, 1528-1532.
44. N. Michaud-Agrawal, E. J. Denning, T. B. Woolf and O. Beckstein, *J Comput Chem*, 2011, **32**, 2319-2327.
45. S. Buchoux, *Bioinformatics*, 2017, **33**, 133-134.
46. A. L. B. Ambrosio, M. C. Nonato, H. S. S. de Araújo, R. Arni, R. J. Ward, C. L. Ownby, D. H. F. de Souza and R. C. Garratt, *Journal of Biological Chemistry*, 2005, **280**, 7326-7335.
47. L. Bitar, D. Jundi, M. Rima, J. Al Alam, J.-M. Sabatier and Z. Fajloun, *Venoms Toxins*, 2021, **1**, 1-12.

48. C. Betzel, T. P. Singh, D. Georgieva and N. Genov, in *Handbook of Metalloproteins*, 2004, DOI: 10.1002/0470028637.met055.
49. V. D. Mouchlis, T. M. Mavromoustakos and G. Kokotos, *J Chem Inf Model*, 2010, **50**, 1589-1601.
50. W. Kopec, A. Żak, D. Jamróz, R. Nakahata, S.-i. Yusa, V. Gapsys and M. Kepczynski, *Langmuir*, 2020, **36**, 12435-12450.
51. M. Kurki, A. Poso, P. Bartos and M. S. Miettinen, *Journal of Chemical Information and Modeling*, 2022, **62**, 6462-6474.
52. N. Kučerka, S. Tristram-Nagle and J. F. Nagle, *The Journal of Membrane Biology*, 2006, **208**, 193-202.
53. P. Jurkiewicz, L. Cwiklik, A. Vojtíšková, P. Jungwirth and M. Hof, *Biochimica et Biophysica Acta (BBA) - Biomembranes*, 2012, **1818**, 609-616.
54. A. Melcrova, S. Pokorna, S. Pullanchery, M. Kohagen, P. Jurkiewicz, M. Hof, P. Jungwirth, P. Cremer and L. Cwiklik, *Scientific Reports*, 2016, **6**, 38035.
55. A. Melcrova, S. Pokorna, S. Pullanchery, M. Kohagen, P. Jurkiewicz, M. Hof, P. Jungwirth, P. S. Cremer and L. Cwiklik, *Sci Rep*, 2016, **6**, 38035.
56. G. Lambeau and M. H. Gelb, *Annu Rev Biochem*, 2008, **77**, 495-520.
